# Probability of vertical HIV transmission: a systematic review and meta-regression

**DOI:** 10.1016/S2352-3018(25)00132-8

**Published:** 2025-07-31

**Authors:** Magdalene K Walters, Michelle A Bulterys, Michael Barry, Sarah Hicks, Ann Richey, Margalit Sabin, Diana Louden, Mary Mahy, John Stover, Robert Glaubius, Hmwe H Kyu, Marie-Claude Boily, Lynne Mofenson, Kathleen Powis, Jeffrey W Imai-Eaton

**Affiliations:** MRC Centre for Global Infectious Disease Analysis, School of Public Health, Imperial College London, London, UK; Department of Epidemiology, School of Public Health, University of Washington, Seattle, WA, USA; Herbert Wertheim School of Public Health, University of California San Diego, La Jolla, CA, USA; Department of Epidemiology, School of Public Health, University of Washington, Seattle, WA, USA; Department of Epidemiology, School of Public Health, University of Washington, Seattle, WA, USA; Department of Epidemiology, School of Public Health, University of Washington, Seattle, WA, USA; Simmons University, Boston, MA, USA; Department of Epidemiology, School of Public Health, University Libraries, University of Washington, Seattle, WA, USA; Data for Impact Department, Joint United Nations Programme on HIV/AIDS, Geneva, Switzerland; Center for Modeling, Planning, and Policy Analysis, Avenir Health, Glastonbury, CT, USA; Center for Modeling, Planning, and Policy Analysis, Avenir Health, Glastonbury, CT, USA; Department of Health Metrics Sciences, School of Medicine, and Institute for Health Metrics and Evaluation, University of Washington, Seattle, WA, USA; MRC Centre for Global Infectious Disease Analysis, School of Public Health, Imperial College London, London, UK; Research Program, Elizabeth Glaser Pediatric AIDS Foundation, Washington, DC, USA; Department of Internal Medicine and Department of Paediatrics, Massachusetts General Hospital, Boston, MA, USA; Department of Immunology and Infectious Diseases, Harvard T H Chan School of Public Health, Boston, MA, USA; MRC Centre for Global Infectious Disease Analysis, School of Public Health, Imperial College London, London, UK; Department of Immunology and Infectious Diseases, Center for Communicable Disease Dynamics, Department of Epidemiology, Harvard T H Chan School of Public Health, Boston, MA, USA

## Abstract

**Background:**

Eliminating HIV vertical transmission is a global priority and monitored by estimating paediatric HIV infections with the UNAIDS-supported Spectrum AIDS Impact Module (Spectrum-AIM). Recent innovations in antiretroviral therapy (ART) service-delivery models and first-line regimens aimed to reduce vertical transmission probabilities. We did a systematic review and meta-analysis to estimate vertical transmission probabilities by maternal immunological and treatment status.

**Methods:**

In this systematic review and meta-regression, we combined an updated systematic review with previous data in meta-regression models to estimate vertical transmission probabilities and determinants. We searched PubMed, Embase, the Global Health Database, WHO Global Index Medicus, CINAHL Complete, and Cochrane CENTRAL for peer-reviewed English-language studies from all regions published between Jan 1, 2018 and Feb 8, 2024, with search term domains mentioning “HIV”, “transmission”, “perinatal”, and “breastfeeding periods”, and “infants born to women living with HIV” or related terms from randomised trials, cohort studies, or observational studies. Four meta-regression models estimated vertical transmission probabilities. We assessed model sensitivity and compared estimates to Spectrum-AIM’s previous results. Finally, we fit a meta-regression model to assess the association of ART class and initiation timing on viral load suppression (VLS) at delivery.

**Findings:**

Of 12 588 potential studies, we identified 24 new studies, which along with the 86 from previous reviews yielded 110 total studies included in meta-regression analysis. For women not receiving ART, higher CD4 count was associated with lower odds of perinatal vertical transmission (odds ratio [OR] 0·80, 95% CI 0·75–0·84, per 100 cells per μL increase). For pregnant women on ART, each additional week on ART before delivery reduced odds of vertical transmission by 5·6% (95% CI 4·2–7·0). The OR of perinatal vertical transmission among pregnant women initiating integrase inhibitor-based ART 20 weeks before delivery was 0·36 (0·14–0·94) compared with those initiating non-nucleoside reverse transcriptase inhibitor (NNRTI)-based ART. This association was confounded by study region. Odds of VLS were lower when ART was initiated late in pregnancy (OR 0·37, 0·21–0·68) for the reference regimen [NNRTI]), without significant difference by ART regimen.

**Interpretation:**

Vertical transmission probability varies by maternal immunological stage, treatment regimen, and timing of treatment initiation. These estimates have been incorporated into Spectrum-AIM for UNAIDS 2025 HIV estimates. Earlier ART initiation is associated with higher odds of VLS at delivery. Further evidence is needed on the effects of recent ART innovations on vertical transmission outcomes.

**Funding:**

National Institutes of Health, UNAIDS, and UK Research and Innovation.

## Introduction

Eliminating HIV vertical transmission and reaching children living with HIV with effective lifelong antiretroviral therapy (ART) are global priorities.^1^ These efforts require accurate data on paediatric HIV infection levels and effectiveness of strategies for prevention of vertical transmission (PVT). Because of incomplete HIV testing among pregnant or breastfeeding women, infants, and children, most countries rely on mathematical models to create estimates, namely the Spectrum AIDS Impact Module (Spectrum-AIM) supported by UNAIDS.^2^ Modelled estimates indicate that vertical transmission has substantially declined over the past two decades, coinciding with successful PVT programme scale-up.^3^

PVT programmes provide pregnant and breastfeeding women living with HIV, and potentially their infants, with antiretrovirals to suppress viral replication.^4–6^ Strategies recommended by WHO have evolved with therapeutic advancements and scientific evidence.^7–9^ Initial 2003 guidelines^10^ recommended short-course antiretrovirals during pregnancy. In 2013 and 2015, WHO recommended immediate lifelong ART initiation first for all pregnant women living with HIV (referred to as option B+) and then for all people living with HIV.^5,6^ To reflect evolving PVT strategies and their efficacies, Spectrum-AIM predicts final vertical transmission rates over time using national data on the number of pregnant women living with HIV,^2^ maternal immunological status, transmission timing (perinatal or breastfeeding), breastfeeding duration among women living with HIV,^11^ and vertical transmission probability according to PVT strategy.^12^ Vertical transmission probabilities are stratified by the following groups: women living with HIV who did not receive any antiretrovirals, women who seroconverted during pregnancy or breastfeeding and did not receive antiretrovirals, women who received short-course antiretrovirals for PVT (maternal single-dose nevirapine [sdNVP],^13^ WHO 2006 dual antiretroviral prophylaxis,^14^ option A,^4^ and option B),^4^ and women on lifelong ART (definitions in the appendix pp 3–4).

Vertical transmission probabilities for Spectrum-AIM were initially estimated in a 2012 systematic review^15^ and subsequently updated with new systematic reviews in 2015^16^ and 2018,^17^ as empirical data on newer PVT strategies accumulated. Although PVT guidelines have not changed since the 2015 immediate lifelong ART recommendation,^6^ innovations in ART regimens and service delivery models have aimed to improve HIV treatment coverage and effectiveness, including PVT. In 2019, dolutegravir was recommended as first-line ART for all people living with HIV, including pregnant women living with HIV.^18^ People living with HIV on dolutegravir achieve rapid viral load suppression (VLS), which can reduce vertical transmission when initiated late in pregnancy compared with previous first-line regimens.^19,20^ Since the 2018 review, universal test and treat and differentiated service delivery have aimed to increase early ART initiation and retention among pregnant and breastfeeding women living with HIV.^21^

Our study updated previous systematic reviews of vertical transmission probabilities with new data since 2018, applied meta-regression to synthesise vertical transmission probabilities from pooled data identified in 2012, 2015, 2018, and 2024 systematic reviews, assessed ART regimen class and weeks on ART before delivery as VLS determinants at delivery, and quantified the effect of updated vertical transmission probabilities on modelled estimated paediatric infections using Spectrum-AIM.

## Methods

### Search strategy and selection criteria

This systematic review and meta-regression analysis was preregistered on PROSPERO (CRD 42024511011) and reported according to PRISMA guidelines (appendix pp 5–8). Ethical approval was obtained from the Imperial College Research Ethics Committee (6300528).

To update previously conducted systematic reviews,^15–17^ we searched PubMed, Embase, Global Health Database, WHO Global Index Medicus, CINAHL Complete, and Cochrane CENTRAL with search term domains mentioning “HIV”, “transmission”, “perinatal” and “breastfeeding periods”, and “infants born to women living with HIV” or related terms. Complete search strategies^22^ are in the appendix (pp 9–13).

Database searches included literature published between Jan 1, 2018 and Feb 8, 2024. The systematic review was managed using Covidence software. Title and abstracts were screened for eligibility and full-text articles reviewed for inclusion by two independent reviewers (MKW, MAB, MB, SH, AR, or MS), with conflicts resolved through consensus. Inclusion criteria were: English-language articles from all geographical regions that reported vertical transmission data from randomised trials, cohort studies, or observational studies. Vertical transmission data needed to be stratified by maternal PVT regimen or maternal CD4 category for women not receiving PVT regimen; if women living with HIV received ART during pregnancy, ART initiation timing (preconception or during pregnancy) was required. Cross-sectional, case–control, case series, case reports, commentaries, letters to editors, study protocols, grey literature, conference abstracts, and non-human animal studies study designs were excluded.

The primary outcome was vertical transmission probability, either explicitly reported in studies or calculated using the proportion of infants diagnosed with HIV among exposed infants during pregnancy or breastfeeding. Extracted data included vertical transmission timing, infant feeding (breast *vs* formula), study characteristics (authors, study title, publication year, geography, and study years), PVT strategy, maternal ART initiation timing, and clinical data (eg, viral load or VLS, and CD4 cell count for women not receiving antiretrovirals; full list in the appendix p 14). Data were independently extracted by two reviewers (MKW and MAB, MB, SH, AR, or MS), resolving discrepancies through consensus.

### Data analysis

We fit four meta-regression models to pooled data from the 2024 and previous systematic reviews to estimate vertical transmission probabilities stratified by the vertical transmission categories in Spectrum-AIM.^16^ The four models estimated vertical transmission probability on the logit scale among different groups: women living with HIV not receiving antiretrovirals by CD4 cell count; women living with HIV who seroconverted during pregnancy or breastfeeding or received short-course antiretrovirals for PVT; women living with HIV exposed to perinatal transmission while receiving lifelong ART by initiation timing; and women living with HIV exposed to breastfeeding transmission receiving lifelong ART. We also assessed ART regimen class as a predictor for vertical transmission probability and whether VLS at delivery (<50 copies per mL) was associated with ART regimen or initiation timing.

Perinatal and breastfeeding vertical transmission definitions remained the same as previous reviews.^15^ Perinatal vertical transmission was defined as HIV acquisition occurring before 6 weeks postpartum. For studies reporting transmission during breastfeeding, we converted cumulative acquisition probabilities to monthly probability for the period starting at the end of the perinatal period (6 weeks) and ending at the time of the last HIV test closest to breastfeeding cessation. Most often this period spanned 6 weeks to 6 months (appendix p 15).

Model 1 estimated vertical transmission among women living with HIV not receiving antiretrovirals as a function of the study population’s baseline CD4 cell count midpoint:

$$logit\left(VT_{BF,CD4,S,Obs}\right)=\beta_{0,BF=0}+\beta_{1,BF=1}+\beta_{2}\;*\;CD4_{\mathrm{Midpoint}}+\beta_{3,BF=1}\;*\;CD4_{\mathrm{Midpoint}}+\mu_{s}+\mu_{\mathrm{Obs}}$$

Model 1 included fixed effects for perinatal ($\beta_{0,BF=0}$) and monthly breastfeeding vertical transmission ($\beta_{1,BF=1}$) intercepts, study population CD4 cell count midpoint ($\beta_{2}$, per 100 cells per μL, centred at 500 cells per μL), and an interaction between CD4 midpoint and breastfeeding timing $\left(\beta_{3,BF=1}\right)$. CD4 midpoint was extracted as the median CD4 count per μL of women living with HIV not receiving antiretrovirals or the midpoint of relevant CD4 range if studies reported vertical transmission by CD4 categories. More information on CD4 midpoint determination from each study is in the appendix (pp 27–30). Random effects were included for study ($\mu_{s}$) and observation ($\mu_{Obs}$).

Model 2 estimated vertical transmission probability among women living with HIV who acquired HIV infection during pregnancy or breastfeeding (maternal seroconversions) or received short-course antiretrovirals for PVT:

$$logit\left(VT_{\mathrm{Categor,TT,S,Obs}}\right)=\beta_{0,\mathrm{Category,TT}}+\mu_{S}+\mu_{\mathrm{Obs}}$$

Model 2 included fixed effects for categories ($\beta_{0,\mathrm{Categor},\mathrm{TT}}$) used in Spectrum-AIM, all stratified by transmission timing: maternal seroconversion, to women living with HIV receiving WHO 2006 dual antiretroviral regimen, sdNVP, option A, and option B. For breastfeeding women receiving sdNVP, transmission rates were stratified by CD4 count lower than 350 cells per μL and CD4 equal to or higher than 350 cells per μL. Random effects were included for study $\left(\mu_{s}\right)$ and observation $\left(\mu_{\mathrm{Obs}}\right)$. Model 2 categories are defined by the common feature that they represented categorical exposures for vertical transmission, but did not involve adjustment for continuous covariates, such as median CD4 count (model 1) or lifelong ART initiation timing (models 3 and 4; appendix pp 3–4 for definition details).

Model 3 estimated perinatal transmission probabilities among women on lifelong ART by maternal ART initiation timing:

$$logit\left(VT_{BF=0,\mathrm{Weeks,late},\mathrm{S,obs}}\right)=\beta_{0}+\beta_{1}\;*\;T_{\mathrm{Week}}+\beta_{2}\;*\;\mathrm{late}\;\mathrm{initiation}\;+\mu_{s}+\mu_{\mathrm{Obs}}$$

Model 3 included a fixed intercept ($\beta_{0}$) representing vertical transmission when ART is initiated 20 weeks before delivery, slope $\left(\beta_{1}\right)$ for weeks on ART before delivery ($T_{\mathrm{Week}}$, centred on ART initiated 20 weeks before delivery; specified as 40 weeks for women already on ART at conception), and a fixed effect for ART initiation within 4 weeks of delivery ($\beta_{2}$, late initiation). The late-initiation effect accounted for increased risk of unsuppressed viral load at delivery when ART is initiated late in pregnancy. $T_{\mathrm{Week}}$ was preferentially extracted as the reported median weeks on ART before delivery, or, otherwise, taken as the range midpoint for studies that reported ART initiation as a range of gestational weeks. We contacted corresponding authors of 26 studies that reported ART initiation timing as a range to request the median ART initiation gestational week. Assumptions to estimate weeks on ART are described in the appendix (pp 31–36). Random effects were included for study $\mu_{s}$ and observation ($\mu_{Obs}$).

Model 4 estimated monthly breastfeeding transmission probabilities by ART initiation timing (preconception or during pregnancy):

$$logit\left(VT_{BF=1,ART,S}\right)=\beta_{0}+\beta_{1}\;*\;ART\;started\;during\;pregnancy\;+\mu_{s}$$

Model 4 included two fixed effects for ART started preconception ($\beta_{0}$) and for ART started during pregnancy $\left(\beta_{1}\right)$. For breastfeeding transmission, ART initiation timing was classified as a binary covariate rather than continuous weeks before delivery (as in model 3 for perinatal transmission) because time on ART during pregnancy is less directly related to VLS during breastfeeding than VLS at delivery. This model included random effects by observation ($\mu_{s}$). Study level random effects were not included because only two studies had multiple observations.

To assess associations between ART regimen class on perinatal transmission probability, we modified model 3 to include fixed effects for ART regimen class (non-nucleoside reverse transcriptase inhibitors [NNRTIs; reference], integrase inhibitors, protease inhibitors, and others). We evaluated geographical region as a confounder of this association (sensitivity analysis, appendix p 36).

Regarding determinants of VLS at delivery, we analysed studies that reported the proportion of women living with HIV with VLS (<50 copies per mL) at delivery, time on ART, and ART regimen class:

$$logit\left(VLS_{BF=0,\mathrm{Class},\mathrm{Time},\mathrm{Obs}}\right)=\beta_{0}+\beta_{1}*\;\mathrm{class}\;+\beta_{2}*\;\mathrm{time}\;+\beta_{3}*\;\mathrm{class}\;*\;\;\mathrm{time}\;+\mu_{s}+\mu_{\mathrm{Obs}}$$

This model included a fixed effect intercept ($\beta_{0}$) for transmission among women initiating NNRTIs early (before second trimester start), fixed effects for ART regimen class relative to NNRTIs for early initiation $\left(\beta_{1}\right)$, timing of ART initiation (late, after the first trimester and before delivery, relative to early), and an interaction between ART class and timing. Study ($\mu_{s}$) and observation ($\mu_{Obs}$) random effects were included.

To assess implications of estimated vertical transmission probabilities for Spectrum-AIM estimates of paediatric HIV infection, we used predicted values from models 1 to 4 to produce vertical transmission probability estimates compatible with stratifications in Spectrum-AIM. For vertical transmission probabilities among women living with HIV not receiving PVT stratified by CD4 categories <200, 200–350, and >350 or higher cells per μL, we used model 1 to predict vertical transmission probabilities corresponding to CD4 midpoint values 100 cells per μL, 275 cells per μL, and 500 cells per μL, respectively. Model 2 was used to predict vertical transmission probabilities for maternal seroconversion and short-course antiretrovirals for PVT. For perinatal vertical transmission probabilities among women on ART for less than or equal to 4 weeks, 5–39 weeks, and preconception, model 3 was used to predict probabilities corresponding to 2 weeks, 20 weeks, and 40 weeks on ART, respectively. Model 4 was used to predict breastfeeding vertical transmission probabilities for women initiating ART before conception or during pregnancy.

Simulations of the effects on paediatric infections were done in an R implementation of the Spectrum-AIM paediatric model that matched Spectrum-AIM version 6.37^23^ in four countries (Burkina Faso, the Democratic Republic of the Congo, Malawi, and Rwanda) in 2000, 2010, 2015, and 2023. Spectrum-AIM files were used for UNAIDS 2024 Global HIV Estimates. Results were compared with the 2024 UNAIDS published paediatric HIV infections, using vertical transmission probabilities used in Spectrum-AIM for UNAIDS estimates published in 2019–24 (appendix pp 3–4; henceforth referred to as the former vertical transmission probabilities).^3^

Analyses were done in R 4.3.1. Mixed effect meta-regression models were fit using the R package glmmTMB version 1.1.9.^24^ CIs were calculated as the 2·5th and 97·5th percentiles of 3000 Monte Carlo samples from each model’s joint posterior distribution. Data and code for model analyses are publicly available.^25^

### Role of the funding source

The funders of the study had no role in study design, data collection, data analysis, data interpretation, or writing of the report.

## Results

**Figure 1:**
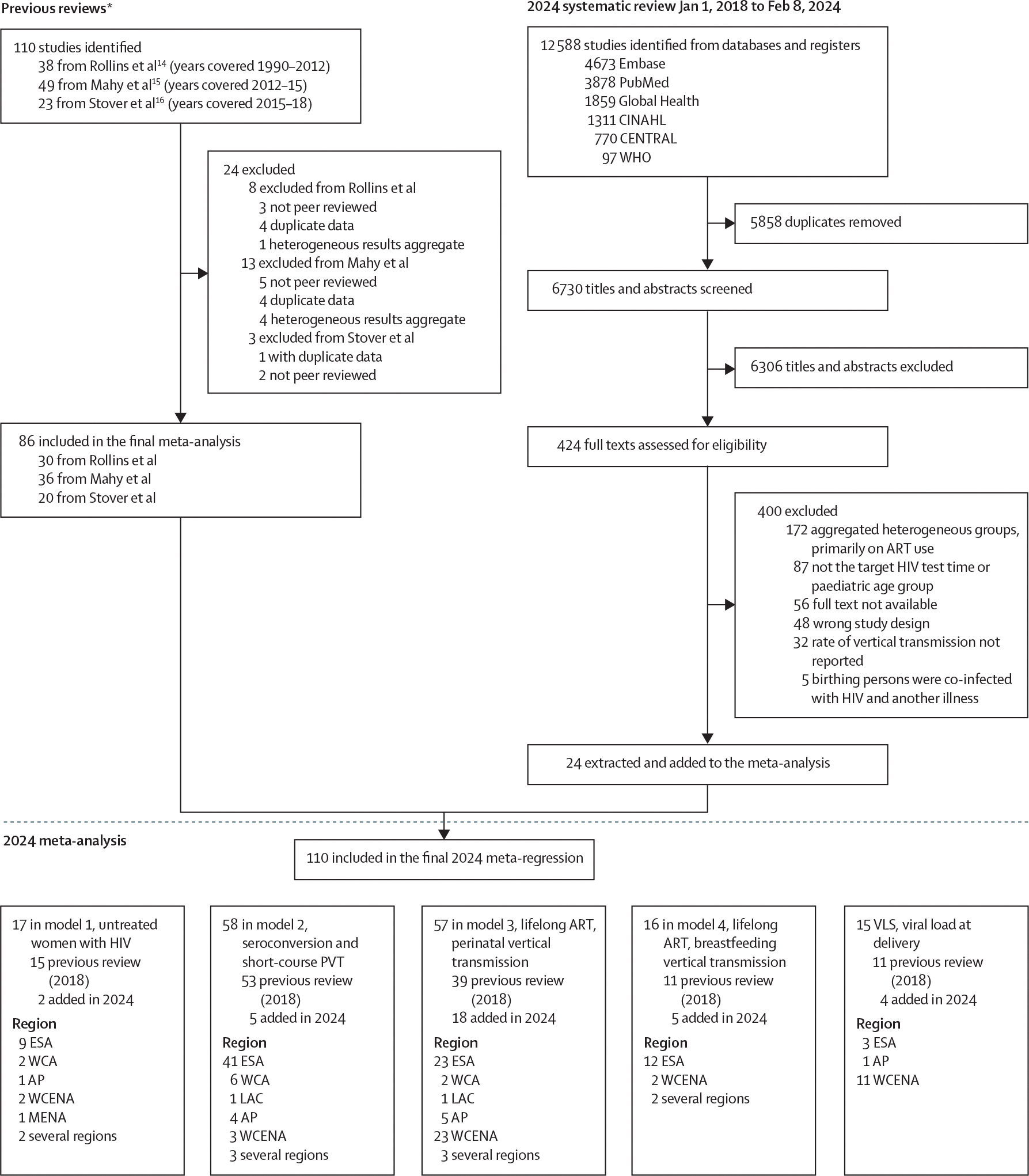
PRISMA flow diagram for the 2018–24 review AP=Asia and Pacific. ART=antiretroviral therapy. ESA=eastern and southern Africa. LAC=Latin America and Caribbean. MENA=Middle East and north Africa. PVT=prevention of vertical transmission. VLS=viral load suppression. WCA=western and central Africa. WCENA=western and central Europe and north America. *Previous reviews informed the 2024 review approach.

Our updated search of 12 588 reviews identified 6730 unique records (figure 1). Data were included from 24 studies. The primary reason for exclusion was reporting vertical transmission aggregated across distinct PVT groups. The data extracted from the 24 new studies published since Jan 1, 2018, were combined with data from 30 studies from Rollin and colleagues^15^ from 2012, 36 studies from Mahy and colleagues^16^ from 2015, and 20 studies from Stover and colleagues^17^ from 2018 (figure 1), yielding 110 studies included in meta-regression analyses. Studies were published between 1988 and 2023 and data were collected between 1982 and 2022. All global regions were represented, but most studies were done in eastern and southern Africa (appendix pp 48–56).

Model 1 included data from 17 studies on vertical transmission among women not receiving PVT by CD4 category, two of which were identified in the 2024 systematic review (figure 1; appendix pp 19–20). 9642 (80·2%) of 12 021 observations were from studies that stratified vertical transmission by CD4 range. Each additional 100 cells per μL in median CD4 was associated with lower vertical transmission odds (odds ratio [OR] 0·8, 95% CI 0·75–0·84; appendix p 16). The interaction between CD4 midpoint and breastfeeding transmission was not statistically significant (appendix p 16). Predicted perinatal transmission probabilities increased with lower maternal CD4 category from around one in six among women with CD4 higher than 350 cells per μL to around one in three among women with CD4 lower than 200 cells per μL (table 1). Monthly transmission probabilities among breastfeeding women increased from around 0·8% per month among untreated women with CD4 of 350 cells per μL or higher to 1% per month among women with CD4 lower than 200 cells per μL (table 2). In sensitivity analyses, fitting the model to only studies that reported study population median CD4, rather than the CD4 range, changed the estimated relationship between CD4 and vertical transmission (appendix p 29). The restricted criteria made the model disproportionately sensitive to observations from three studies published between 1991 and 1998 reporting high transmission, because 80·2% of all observations were omitted (appendix pp 27–30).

Model 2 included data from 58 studies on vertical transmission among women who seroconverted or received short-course antiretrovirals for PVT, four of 58 identified in the 2024 review (figure 1; appendix pp 21–24). Around one in five women who seroconverted during pregnancy transmitted HIV perinatally (table 1) and nearly one in three who seroconverted during breastfeeding (table 2). Perinatal transmission probabilities were lower among women who received short-course antiretrovirals for PVT. Short-course antiretroviral strategies recommended in earlier guidelines (ie, sdNVP, dual antiretroviral, and option A) were less effective at averting vertical transmission and had higher vertical transmission probabilities than those used more recently (option B) for both perinatal (table 1) and breastfeeding transmission (table 2).

**Figure 2:**
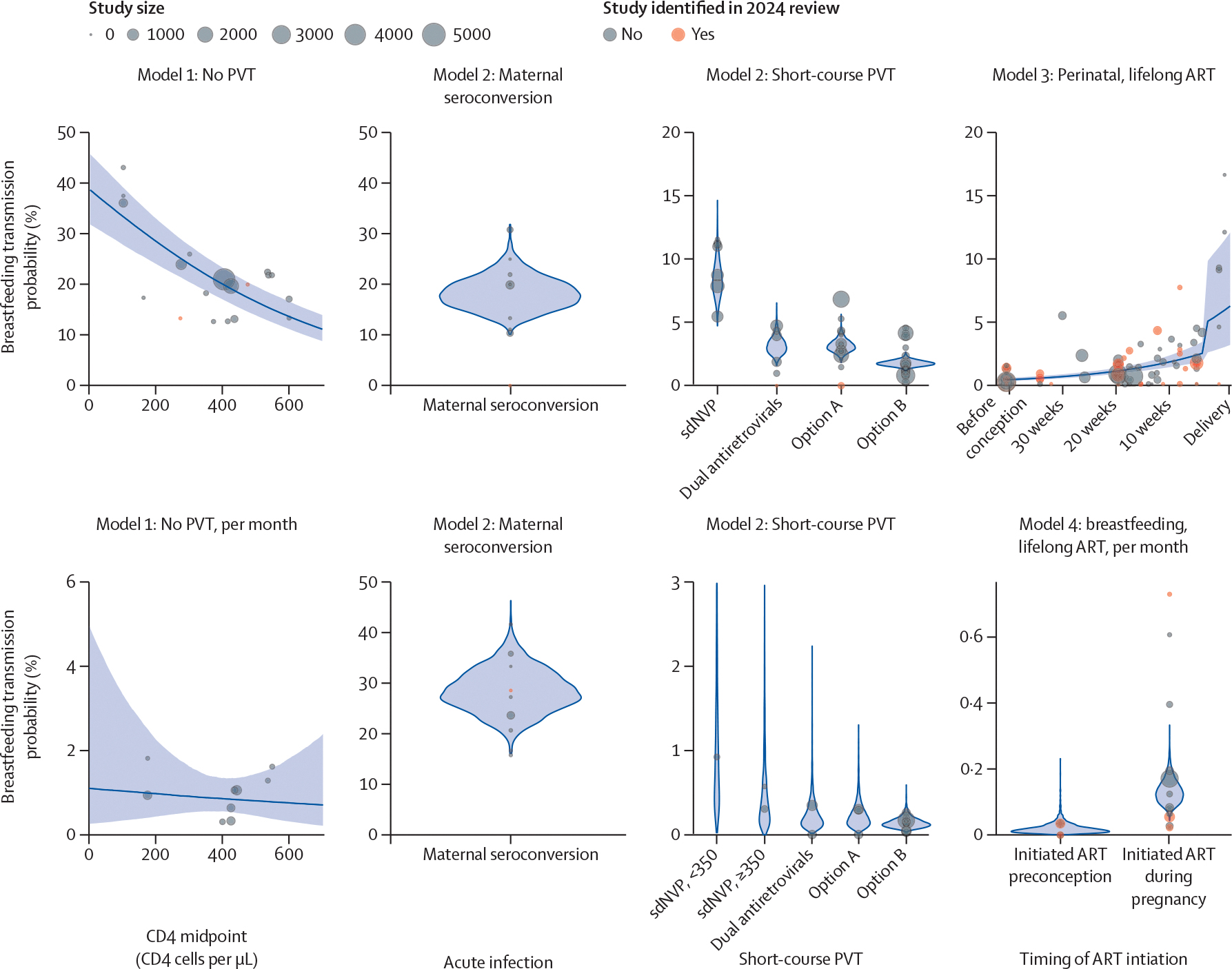
Data used in models 1–4 and model estimates of vertical transmission probability Point size reflects study size and colour indicates whether the study was identified in the 2024 systematic review. Blue violin plots are used for categorical models (models 2 and 4) and blue lines with ribbon are used for continuous models (models 1 and 3). Panels represent the different models used to produce estimates of vertical transmission probability compatible with Spectrum-AIDS Impact Module, with the top row displaying perinatal vertical transmission probabilities and the bottom row displaying breastfeeding vertical transmission probabilities. ART=antiretroviral therapy. PVT=prevention of vertical transmission. sdNVP=single-dose nevirapine.

Model 3 included data from 57 studies on perinatal transmission among women on lifelong ART, 15 of 57 identified in the 2024 systematic review (figures 1, 2; appendix p 25). Five of 26 studies provided additional unpublished data on median gestational week at ART initiation. Each additional week on ART before delivery reduced vertical transmission odds by 5·6% (95% CI 4·2–7·0; appendix p 16). Perinatal vertical transmission probability was lowest among women who initiated ART preconception and highest among women who initiated in the last 4 weeks of pregnancy (table 1). When model 3 was restricted to only studies that reported the median weeks on ART (rather than a range of weeks when ART was initiated) the odds ratio for less than 4 weeks compared with 20 weeks was 10·1 (6·6–16·1; appendix pp 31–36).

Model 4 included data from 16 studies on breastfeeding transmission among women on lifelong ART, 5 of 16 identified in the 2024 systematic review (figure 1; appendix p 26). The monthly breastfeeding transmission probability was 0·13% (0·08–0·22) for women who initiated ART during pregnancy and 0·02% (0–0·06) for women who initiated ART preconception (table 2).

When refitting model 3 with fixed effects for ART regimen class, women living with HIV who initiated integrase inhibitors versus an NNRTI-based regimen had significantly lower vertical transmission odds (OR: 0·36 (0·14–0·94), appendix p 17). After adding geographical region to the model, associations with ART regimen were no longer statistically significant (appendix p 36).

15 studies reported data on the proportion of women living with HIV with viral load of less than 50 copies per mL at delivery; three were identified in the 2024 systematic review (figure 1). Most studies were from western and central Europe and north America. VLS probability at delivery was highest among women living with HIV who initiated integrase inhibitor-based regimens before the second trimester (appendix p 17); however, the difference between regimen classes was not statistically significant. VLS probability among women who started ART before the second trimester was 90·6% (95% CI 80·4–95·4) for NNRTIs, 79·2% (42·3–95·0) for protease inhibitors, and 90·0% (62·9–97·8) for other regimens. Odds of VLS were lower when ART was initiated after the first trimester (OR 0·37, 0·21–0·68) for the reference regimen [NNRTI]), without significant difference by ART regimen. VLS probability among women who initiated ART after the first trimester was 40·4% (3·3–90·2) for integrase inhibitors, 82·8% (70·2–90·4) for NNRTIs, 65·6% (51·7–76·5) for protease inhibitors, and 74·3% (2·8–99·6) for other regimens.

We compared the updated vertical transmission probabilities with the former default vertical transmission probabilities in Spectrum-AIM (tables 1, 2). The mean percent difference was 2%. The percent difference was largest for monthly breastfeeding transmission probability among women with CD4 higher than 350 cells per μL (table 2). Updated perinatal transmission probabilities were on average lower, whereas updated breastfeeding transmission probabilities were on average higher.

**Figure 3:**
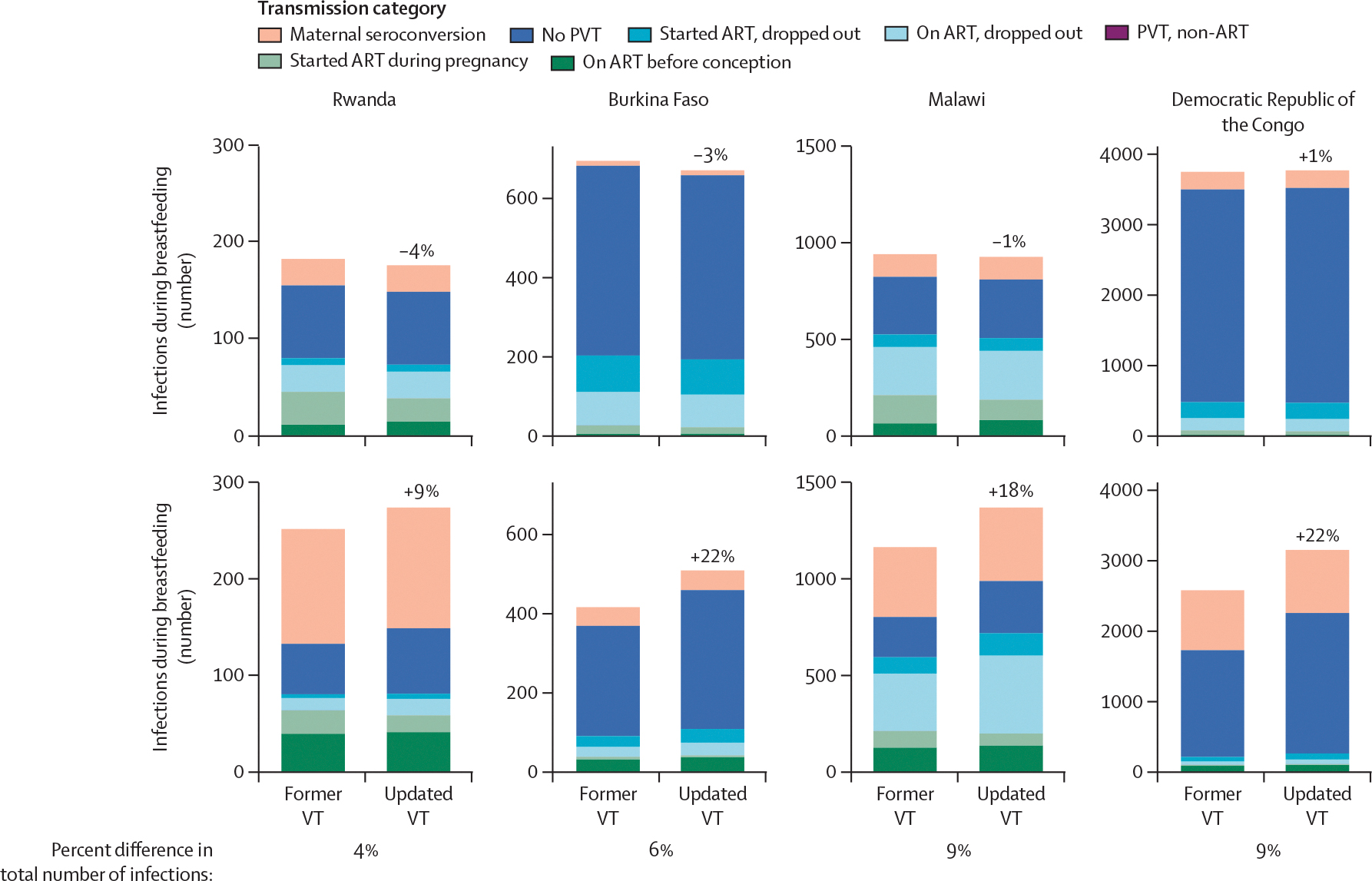
Differences in the 2023 number of paediatric infections using the Spectrum-AIDS Impact Module former versus updated VT probabilities Coloured percentage points represent the percent difference between the infections using the former versus updated VT probabilities. ART=antiretroviral therapy. PVT=prevention of vertical transmission. VT=vertical transmission.

We incorporated the updated vertical transmission probabilities (tables 1, 2) into Spectrum-AIM to estimate the number of paediatric infections in Burkina Faso, the Democratic Republic of the Congo, Malawi, and Rwanda (appendix pp 38–46). In 2023, total paediatric infections (perinatal and breastfeeding) from the updated vertical transmission probabilities were 8·8% higher than those estimated from the former vertical transmission probabilities (figure 3). Perinatal infections were slightly lower except in the Democratic Republic of the Congo (average 2·5% lower) and breastfeeding infections were higher in all countries (average 15·6% higher; figure 3). Increases in estimated vertical transmission infections during breastfeeding were largest among women who did not receive short-course antiretrovirals for PVT or experienced treatment interruptions (table 2).

## Discussion

We systematically updated evidence about HIV vertical transmission determinants and probabilities, including the effectiveness of antiretroviral-based strategies to prevent transmission. This information is crucial to produce modelled estimates of paediatric HIV infections and guide strategies to eliminate vertical transmission. Since the 2018 review, 24 studies were published with data relevant to inform parameters in Spectrum-AIM. Compared with previous assessments, we applied a more systematic meta-regression approach that strengthened pooling of information and quantified statistical uncertainty for the first time.

Patterns of vertical transmission probabilities were consistent with previous reviews.^15–17^ Vertical transmission was highest among women not receiving PVT, lower among women receiving short-course antiretrovirals for PVT, and lowest among women initiating ART during and before pregnancy. Changes from former default values^2^ are attributable to both new data since the previous review and the meta-regression analytical approach. Perinatal vertical transmission probabilities are slightly lower than reported in previous reviews.^17^ Of perinatal vertical transmission probabilities, vertical transmission probability among women initiating ART within 4 weeks was the largest relative decrease (8·2% to 5·6%); only 1% of pregnant women living with HIV globally initiate ART within 4 weeks of delivery, so this change will not substantially change estimates of perinatal infections. Updated breastfeeding vertical transmission probabilities were higher than previous values. The largest change was among women who did not receive any antiretrovirals for PVT for which assumed probabilities had not been updated since the 2012 review.

The vertical transmission probabilities derived here have been adopted as default values recommended for countries using Spectrum-AIM to update national HIV estimates in 2025. These new values are unlikely to substantially change the number of perinatal infections but will increase estimated infections during breastfeeding. The shift in infection timing to the breastfeeding period has implications for HIV testing strategies. Retaining mother–infant pairs in PVT services until breastfeeding cessation is important to prevent transmission and monitor HIV outcomes. Additionally, universal final HIV outcome ascertainment could be used to compare programmatic data to estimates of new infections from Spectrum-AIM.

We evaluated emerging evidence on scale-up of universal test and treat, differentiated service delivery models, and dolutegravir-based first-line regimens at increasing VLS and reducing vertical transmission among pregnant and breastfeeding women living with HIV.^26^ We found scarce data to assess these. ART initiation before the second trimester was associated with higher VLS at delivery, consistent with observed lower transmission probability among women who initiated ART earlier. However, data on VLS at delivery were predominantly limited to studies conducted in high-income countries and where women initiated integrase inhibitor-based regimens early in pregnancy.

Regarding differences by ART regimen, women who initiated integrase inhibitor-based regimens (including dolutegravir) had the lowest perinatal vertical transmission probability. Data were insufficient to assess the effect of antiretroviral drug class on vertical transmission probability during breastfeeding. These results should be interpreted cautiously as studies with integrase inhibitor-based regimens were predominately in high-income countries; only one study reporting vertical transmission probability among women receiving integrase inhibitor-based regimens was done in Africa. The difference in vertical transmission probability by regimen was attenuated and not statistically significant when adjusting for geographical region. Moreover, data indicating different vertical transmission rates associated with integrase inhibitor-based regimens were predominately among women initiating ART before or early in pregnancy, whereas cohort studies comparing regimens among the same study populations have found no regimen differences in vertical transmission and VLS among women when ART is initiated early,^27^ but with potential benefits when ART is initiated late because of faster VLS associated with integrase inhibitor-based regimens. As such, we believe additional data among women initiating integrase inhibitors late in pregnancy are needed before recommending modelling vertical transmission probability by ART regimen.

Our systematic review has limitations. Restricting to studies that disaggregated vertical transmission by PVT strategy and ART initiation timing excluded 43% of full texts that reported unstratified vertical transmission. Individual level information about maternal CD4 cell counts or ART initiation timing and vertical transmission outcomes might have quantified these relationships more precisely than the broad categories from our systematic review. The association between CD4 cell count and vertical transmission probability was significantly different in studies that reported CD4 range versus CD4 midpoint, driven by three studies done in the 1990s that reported CD4 range and very high vertical transmission that accounted for only 6% of observations from model 1. Regarding ART initiation timing, the relationship between weeks on ART and vertical transmission did not differ between reporting median weeks versus range of weeks. Only including studies that reported median weeks would have excluded 76% of all studies that reported ART initiation occurring during the third trimester. We did not estimate vertical transmission probability among women who initiated ART after seroconverting during pregnancy or postpartum, an area for future work as identifying infections during these periods is an increasing focus of PVT programmes through retesting at late antenatal care visits, labour and delivery, and postnatally.

In conclusion, vertical HIV transmission probability varies substantially according to maternal clinical characteristics and ART initiation timing. Our analysis incorporated more data through an updated systematic literature search and refined formal statistical synthesis. We found somewhat higher transmission rates during breastfeeding than previous estimates. Findings reinforce the importance of initiating women living with HIV on ART early in pregnancy to progress towards HIV vertical transmission elimination. However, data were limited to quantify the effects of recent innovations in ART regimens and service delivery models on vertical transmission rates; this should continue to be assessed as new data emerge.

## Supplementary Material

1

## Figures and Tables

**Table 1:** Perinatal vertical transmission probabilities by PVT regimen

	Former vertical transmission probability (studies published before 2018)	Updated vertical transmission probability (studies published before Feb 8, 2024)	Percent change*	Percent of pregnant women living with HIV in each stratum		
				2010†	2015†	2023†

**Model 1: vertical transmission probability for women not receiving PVT**						
0–199 cells/μL	37·0%	33·4% (27·8-39·1)	−10%	8%	3%	2%
200–350 cells/μL	27·0%	25·1% (21·4-28·9)	−7%	12%	5%	4%
>350 cells/μL	15·0%	16·6% (13·9-19·7)	+11%	21%	12%	12%
**Model 2: vertical transmission probability from maternal seroconversion or short-course PVT**						
Infection	18·1%	18·1% (12·8-25·2)	0%			
sdNVP	7·5%	8·3% (5·9-11·3)	+11%	11%	1%	0%
Dual antiretroviral	2·2%	3·1% (2·1-4·7)	+41%	15%	0%	0%
Option A	4·1%	3·1% (2·3-4·2)	−24%	11%	2%	0%
Option B	1·9%	1·8% (1·4-2·3)	−5%	3%	4%	0%
**Model 3: perinatal transmission probability from women receiving ART by timing of initiation**						
Option B+, on ART ≤4 weeks	8·2%	5·6% (2·8-10·9)	−32%	1%	3%	1%
Option B+, on ART 5–39 weeks	1·4%	1·0% (0·8-1·3)	−29%	9%	36%	25%
Option B+, on ART before conception	0·26%	0·33% (0·23-0·48)	+27%	9%	34%	56%

ART=antiretroviral therapy. PVT=prevention of vertical transmission. sdNVP=single-dose nevirapine.

* Negative percentages represent a percent decrease from the former value, whereas positive values represent a percent increase from the former value.

† The proportion of women living with HIV who fall into each category each year globally.

**Table 2:** Breastfeeding vertical transmission probabilities by PVT regimen

	Former vertical transmission probability (studies published before 2018)	Updated vertical transmission probability (studies published before Feb 8, 2024)	Percent change*	Percent of pregnant women living with HIV in each stratum		
				2010†	2015†	2023†

**Model 1: vertical transmission probability for women not receiving PVT**						
0–199 cells/μL	0·89%	1·02% (0·32-3·32)	+15%	8%	3%	2%
200–350 cells/μL	0·81%	0·90% (0·49-1·73)	+11%	12%	5%	4%
>350 cells/μL	0·51%	0·79% (0·43-1·43)	+55%	21%	12%	12%
**Model 2: vertical transmission probability from maternal seroconversion or short-course PVT**						
Infection (one time, not monthly)	26·9%	28·29% (20·82-37·20)	+5%			
sdNVP <350 cells/μL	0·99%	0·89% (0·16-4·45)	−10%	11%‡	1%‡	0%‡
sdNVP ≥350 cells/μL	0·40%	0·35% (0·06-1·94)	−13%	11%‡	1%‡	0%‡
Dual antiretroviral	0·18%	0·20% (0·06-0·74)	+11%	15%	0%	0%
Option A	0·20%	0·21% (0·06-0·63)	+5%	11%	2%	0%
Option B	0·13%	0·14% (0·07-0·30)	+8%	3%	4%	0%
**Model 4: monthly breastfeeding transmission for women receiving lifelong ART**						
Option B+, on ART ≤4 weeks	0·20%	0·13%§ (0·08-0·22)	−35%	1%	3%	1%
Option B+, on ART 5–39 weeks	0·11%	0·13%§ (0·08-0·22)	+18%	9%	36%	25%
Option B+, on ART before conception	0·02%	0·02% (0·00-0·06)	0%	9%	34%	56%

All breastfeeding vertical transmission probabilities are per month, except for maternal seroconversions, which is a one-time probability for breastfeeding women living with HIV. ART=antiretroviral therapy. PVT=prevention of vertical transmission. sdNVP=single-dose nevirapine.

* Negative percentages represent a percent decrease from the former value, whereas positive values represent a percent increase from the former value.

† The proportion of women living with HIV who fall into each category each year globally.

‡ These proportions represent women with any CD4 count who received sdNVP.

§ These values were estimated as monthly breastfeeding transmission probability among women who initiated ART at any time during pregnancy.

## Data Availability

Data used in this analysis are publicly available from: https://doi.org/10.5281/zenodo.15166513.^26^

## Research in context

### Evidence before this study

Three previous systematic reviews have assessed vertical transmission probability to inform assumptions for modelled estimates of perinatal and breastfeeding vertical HIV transmission with the Spectrum AIDS Impact Module (Spectrum-AIM). The most recent systematic review, done in 2018, calculated vertical transmission probabilities as the weighted average of literature published before 2018. Since 2018, dolutegravir has been widely adopted as a first-line antiretroviral for all people living with HIV, differentiated service-delivery models for pregnant and breastfeeding women have aimed to improve care engagement, and immediate lifelong antiretroviral therapy initiation following HIV diagnosis has become standard for all people living with HIV. These biomedical and service delivery interventions aim to increase the proportion of pregnant and breastfeeding women who are virally suppressed before delivery, but evidence is scarce about their effect on vertical transmission outcomes. To identify any previous systematic review and meta-regression studies addressing vertical transmission rates and effects of these innovations therein, we searched PubMed from database inception to Jan 4, 2025, for published articles using the search terms (“vertical transmission” OR “mother-to-child transmission” OR “MTCT” AND “HIV” AND “systematic review” AND “meta-regression” OR “meta-analysis”), resulting in 27 relevant articles. No articles estimated vertical transmission probability stratified by prevention of vertical transmission intervention. Articles that estimated cumulative vertical transmission probabilities were restricted to a country or a region.

### Added value of this study

This study systematically reviewed literature published before Feb 8, 2024, on HIV vertical transmission probabilities stratified by maternal clinical or treatment status. To our knowledge, this is the first analysis using meta-regression to estimate vertical transmission probabilities that are compatible with Spectrum-AIM. Compared with previous analyses, the meta-regression approach improves accounting for between-study heterogeneity and quantifies statistical uncertainty associated with the vertical transmission probabilities used in Spectrum-AIM. Overall patterns of relative vertical transmission probabilities were similar to previous reviews, but we estimated marginally higher transmission probabilities among breastfeeding women. Initiation of antiretrovirals before the second trimester of pregnancy was associated with higher odds of maternal viral load suppression at delivery. The systematic review identified limited new data about effects of new first-line drug regimens or service-delivery models outside of high-income settings.

### Implications of all the available evidence

When implemented in Spectrum-AIM, the vertical transmission probabilities estimated here result in more infections during breastfeeding than previous estimates. This finding underscores the importance of testing infants exposed to HIV for final HIV status outcome after breastfeeding has ended. Additionally, our results show the importance of initiating antiretrovirals as early as possible during or before pregnancy to preserve the woman’s health and achieve viral suppression by delivery to reduce the probability of vertical transmission. Further evidence is needed on the effect of integrase inhibitor-based regimen on vertical transmission in diverse global regions.
