# Supplementary material for: Probability of vertical HIV transmission: a systematic review and meta-regression
Source: Lancet HIV. Author manuscript; Available in PMC 2025 Dec 22. (PMC12720210; doi:10.1016/S2352-3018(25)00132-8)
Supplement: 1 [file NIHMS2122997-supplement-1.pdf]

THE LANCET
HIV

Supplementary appendix

This appendix formed part of the original submission and has been peer reviewed. We post it as supplied by the authors.

Supplement to: Walters MK, Bulterys MA, Barry M, et al. Probability of vertical HIV transmission: a systematic review and meta-regression. *Lancet Diabetes Endocrinol* 2025; published online July 31. https://doi.org/10.1016/S2352-3018(25)00132-8.

Appendix

Probability of vertical HIV transmission: A systematic review and meta-regression

Magdalene K. Walters MPH,[1]* Michelle A. Bulterys PhD,[2,3]* Michael Barry PhD,[2] Sarah Hicks MPH,[2] Ann Richey MPH,[2] Margalit Sabin BA,[5] Diana Louden MLib,[4] Mary Mahy ScD,[6] John Stover MA,[7] Robert Glaubius PhD,[7] Hmwe Kyu PhD,[8] Prof Marie-Claude Boily PhD,[1] Lynne Mofenson MD,[9] Kathleen Powis MD,[10,11] Jeffrey W. Imai-Eaton PhD[1,12]

*Authors contributed equally

[1] MRC Centre for Global Infectious Disease Analysis, School of Public Health, Imperial College London, London, United Kingdom (Magdalene K. Walters MPH, Prof Marie-Claude Boily PhD, Jeffrey W. Imai-Eaton PhD)

[2] Department of Epidemiology, School of Public Health, University of Washington, Seattle, WA, USA (Michelle A. Bulterys PhD, Michael Barry PhD, Sarah Hicks MPH, Ann Richey MPH)

[3] Herbert Wertheim School of Public Health, University of California San Diego, La Jolla, CA, USA (Michelle A. Bulterys PhD)

[4] University Libraries, University of Washington, Seattle, WA, USA (Diana Louden MLib)

[5] Simmons University, Boston, MA, USA (Margalit Sabin BA)

[6] Data for Impact Department, Joint United Nations Programme on HIV/AIDS, Geneva, Switzerland (Mary Mahy ScD)

[7] Center for Modeling, Planning and Policy Analysis, Avenir Health, Glastonbury, CT, USA (John Stover MA, Robert Glaubius PhD)

[8] Department of Health Metrics Sciences, School of Medicine, and Institute for Health Metrics and Evaluation, University of Washington, Seattle, WA, USA (Hmwe Kyu PhD)

[9] Research Program, Elizabeth Glaser Pediatric AIDS Foundation, Washington, DC, USA (Lynne Mofenson MD)

[10] Departments of Internal Medicine and Paediatrics, Massachusetts General Hospital, Boston, MA, USA (Kathleen Powis MD)

[11] Department of Immunology and Infectious Diseases, Harvard T.H. Chan School of Public Health, Boston, MA, USA (Kathleen Powis MD)

[12] Center for Communicable Disease Dynamics, Department of Epidemiology, Harvard T.H. Chan School of Public Health, Boston, MA, USA (Jeffrey W. Imai-Eaton PhD)

Contents

1. Definitions, VT probabilities used from 2019-2014, and source of vertical transmission probabilities used in Spectrum-AIM 3
2. 2024 systematic review 5
2.2. PRISMA checklist 5
2.3. Search strategy by data source 9
2.4. Variables extracted in systematic review 14
2.5. Perinatal transmission probability definition 15
2.6. Monthly breastfeeding transmission probability definition 15
3. Meta-regression model estimates 16
3.1 Regression tables for all models 16
3.2 Study-level and pooled estimates of vertical transmission probability 18
4. Sensitivity analyses on meta-regression model assumptions 27
4.1. Model one: VT probability from women not receiving PVT 27
4.2. Model three: perinatal transmission probability from women receiving ART by timing of initiation 31
4.3 Geographic region as a confounder of ART class's effect on VT probability 36
5. Implications of estimated VT probabilities for Spectrum-AIM's estimates of paediatric HIV infections 37
6. Data included in meta-regression analysis 47
6.1 Study references 57
7. References 67

1. Definitions, VT probabilities used from 2019-2014, and source of vertical transmission probabilities used in Spectrum-AIM

Table 1.1 describes the definition of vertical transmission categories in the Spectrum-AIM model[1] and reports the VT probabilities used in model versions for UNAIDS global HIV estimates published 2019 through 2024 and their sources. Most VT probabilities used between 2019-2024 values are weighted averages of studies identified in the 2018 review by Mofenson.[2] Estimates of perinatal transmission probability for women who did not receive PVT have not been updated since the 2012 Rollins *et al.* systematic review.[3]

Table 1.1. Default perinatal vertical HIV transmission probabilities in Spectrum-AIM model estimates published by UNAIDS from 2019-2024

Transmission category	Definition	VT probability	Source of VT probability
**CD4 < 200**	Existing infection, mother did not use PVT and had a CD4 < 200.	0.37	Median of studies from Rollins *et al.* 2012 systematic review
**CD4 200-350**	Existing infection, mother did not use PVT and had a CD4 200-350.	0.27	Median of studies from Rollins *et al.* 2012 systematic review
**CD4 ≥ 350**	Existing infection, mother did not use PVT and had a CD4 > 350.	0.15	Median of studies from Rollins *et al.* 2012 systematic review
**Maternal seroconversion**	Mother seroconverted during pregnancy and did not receive ARVs.	0.181	Weighted average from Mofenson 2018 systematic review update
**Maternal single dose nevirapine**	Mother received only single dose nevirapine as part of PVT.	0.075	Weighted average from Mofenson 2018 systematic review update
**WHO 2006 Dual ARV regimen**	Mother utilized two ARV regimens for PVT.	0.022	Weighted average of studies with women with CD4 > 350 from Mofenson 2018 systematic review update
**Option A**	Mothers with CD4 > 350 used AZT from week 14 of gestation and single dose nevirapine at the onset of labor. Daily AZT/ 3TC used through 7 days postpartum.	0.041	Weighted average from Mofenson 2015 systematic review update
**Option B**	Mothers with CD4 > 350 used triple ARVs starting at week 14 of gestation and continued through breastfeeding cessation.	0.019	Weighted average of studies with breastfeeding populations from Mofenson 2018 systematic review update
**Mother on ART <4 weeks before delivery**	Triple ARVs were initiated <4 weeks before delivery and continued for life.	0.082	Weighted average from Mofenson 2018 systematic review update
**Mother on ART >4 weeks before delivery**	Triple ARVs were initiated >4 weeks before delivery (but after conception) and continued for life.	0.014	Weighted average from Mofenson 2018 systematic review update
**Mother on ART preconception**	Mother was on triple ARVs at conception and continued for life.	0.0026	Weighted average from Mofenson 2018 systematic review update

Table 1.2. Default vertical transmission probabilities among breastfeeding women in Spectrum-AIM model estimates published by UNAIDS from 2019-2024

Transmission category	Definition	VT probability	Source of VT probability
**CD4 < 200 (monthly)**	Existing infection, mother did not use PVT and had a CD4 < 200.	0.0089	Weighted average from Mofenson 2018 systematic review update of studies without a CD4 restriction
**CD4 200-350 (monthly)**	Existing infection, mother did not use PVT and had a CD4 200-350.	0.0081	It is unclear where this VT probability came from, this was not explicitly estimated in the 2012, 2015, or 2018 reviews.
**CD4 ≥ 350 (monthly)**	Existing infection, mother did not use PVT and had a CD4 > 350.	0.0051	Rollins *et al.* 2012 systematic review
**Maternal seroconversion**	Mother seroconverted during pregnancy and did not receive ARVs.	0.269	Weighted average from Mofenson 2018 systematic review update
**Maternal single dose nevirapine CD4 < 350 (monthly)**	Mother received only single dose nevirapine as part of PVT.	0.0099	Weighted average from Mofenson 2018 systematic review update
**Maternal single dose nevirapine CD4 ≥ 350 (monthly)**	Mother received only single dose nevirapine as part of PVT.	0.004	Weighted average from Mofenson 2018 systematic review update
**WHO 2006 Dual ARV regimen (monthly)**	Mother utilized two ARV regimens for PVT.	0.0018	Weighted average of studies that reported extended infant prophylaxis from Mofenson 2018 systematic review update
**Option A (monthly)**	Mothers with CD4 > 350 used AZT from week 14 of gestation and single dose nevirapine at the onset of labor. Daily AZT/ 3TC used through 7 days postpartum.	0.002	Median of studies in the Rollins *et al.* 2012 systematic review.
**Option B (monthly)**	Mothers with CD4 > 350 used triple ARVs starting at week 14 and continued through breastfeeding cessation.	0.0013	It is unclear where this VT probability came from, the Mofenson 2018 systematic review had a weighted average of 0.0011.
**On ART <4 weeks before delivery (monthly)**	Triple ARVs were initiated <4 weeks before delivery and continued for life.	0.002	Expert opinion from Rollins *et al.* 2012 systematic review
**On ART >4 weeks before delivery (monthly)**	Triple ARVs were initiated >4 weeks before delivery (but after conception) and continued for life.	0.0011	Weighted average of studies where mother started ART at any time during pregnancy from Mofenson 2018 systematic review update
**On ART preconception (monthly)**	Mother was on triple ARVs at conception and continued for life.	0.0002	Weighted average of studies from Mofenson 2018 systematic review update

2. 2024 systematic review

2.2. PRISMA checklist

Table 2.2.1. PRISMA checklist

Topic	No.	Summary	Location
**Title**	1	Identify the report as a systematic review.	Title
**Abstract**	2	See the PRISMA 2020 for Abstracts checklist	Included in Table 2.2.2
**INTRODUCTION**			
**Rationale**	3	Describe the rationale for the review in the context of existing knowledge.	Introduction
**Objectives**	4	Provide an explicit statement of the objective(s) or question(s) the review addresses.	Introduction
**METHODS**			
**Eligibility criteria**	5	Specify the inclusion and exclusion criteria for the review and how studies were grouped for the syntheses.	Methods
**Information sources**	6	Specify all databases, registers, websites, organisations, reference lists and other sources searched or consulted to identify studies. Specify the date when each source was last searched or consulted.	Methods
**Search strategy**	7	Present the full search strategies for all databases, registers and websites, including any filters and limits used.	Methods and Appendix 2.3
**Selection process**	8	Specify the methods used to decide whether a study met the inclusion criteria of the review, including how many reviewers screened each record and each report retrieved, whether they worked independently, and if applicable, details of automation tools used in the process.	Methods and Figure 1
**Data collection process**	9	Specify the methods used to collect data from reports, including how many reviewers collected data from each report, whether they worked independently, any processes for obtaining or confirming data from study investigators, and if applicable, details of automation tools used in the process.	Methods
**Data items**	10a	List and define all outcomes for which data were sought. Specify whether all results that were compatible with each outcome domain in each study were sought (e.g. for all measures, time points, analyses), and if not, the methods used to decide which results to collect.	Methods and Appendix 2.4
	10b	List and define all other variables for which data were sought (e.g. participant and intervention characteristics, funding sources). Describe any assumptions made about any missing or unclear information.	Methods and Appendix 2.4
**Study risk of bias assessment**	11	Specify the methods used to assess risk of bias in the included studies, including details of the tool(s) used, how many reviewers assessed each study and whether they worked independently, and if applicable, details of automation tools used in the process.	Not applicable

**Effect measures**	12	Specify for each outcome the effect measure(s) (e.g. risk ratio, mean difference) used in the synthesis or presentation of results.	Methods and Appendix 2.5 and 2.6
**Synthesis methods**	13a	Describe the processes used to decide which studies were eligible for each synthesis (e.g. tabulating the study intervention characteristics and comparing against the planned groups for each synthesis (item 5)).	Methods
	13b	Describe any methods required to prepare the data for presentation or synthesis, such as handling of missing summary statistics, or data conversions.	Methods and Appendix 4
	13c	Describe any methods used to tabulate or visually display results of individual studies and syntheses.	Methods and Appendix 4.2
	13d	Describe any methods used to synthesize results and provide a rationale for the choice(s). If meta-analysis was performed, describe the model(s), method(s) to identify the presence and extent of statistical heterogeneity, and software package(s) used.	Methods
	13e	Describe any methods used to explore possible causes of heterogeneity among study results (e.g. subgroup analysis, meta-regression).	Methods and Appendix 5
	13f	Describe any sensitivity analyses conducted to assess robustness of the synthesized results.	Methods and Appendix 5
**Reporting bias assessment**	14	Describe any methods used to assess risk of bias due to missing results in a synthesis (arising from reporting biases).	Not applicable
**Certainty assessment**	15	Describe any methods used to assess certainty (or confidence) in the body of evidence for an outcome.	Methods
**RESULTS**			
**Study selection**	16a	Describe the results of the search and selection process, from the number of records identified in the search to the number of studies included in the review, ideally using a flow diagram.	Results and Figure 1
	16b	Cite studies that might appear to meet the inclusion criteria, but which were excluded, and explain why they were excluded.	Results and Figure 1
**Study characteristics**	17	Cite each included study and present its characteristics.	Appendix 3
**Risk of bias in studies**	18	Present assessments of risk of bias for each included study.	Not applicable
**Results of individual studies**	19	For all outcomes, present, for each study: (a) summary statistics for each group (where appropriate) and (b) an effect estimate and its precision (e.g. confidence/credible interval), ideally using structured tables or plots.	Appendix 4.2
**Results of syntheses**	20a	For each synthesis, briefly summarise the characteristics and risk of bias among contributing studies.	Not applicable
	20b	Present results of all statistical syntheses conducted. If meta-analysis was done, present for each the summary estimate and its precision (e.g. confidence/credible	Results and Appendix 4

		interval) and measures of statistical heterogeneity. If comparing groups, describe the direction of the effect.	
	20c	Present results of all investigations of possible causes of heterogeneity among study results.	Results and Appendix 4 and 5
	20d	Present results of all sensitivity analyses conducted to assess the robustness of the synthesized results.	Results and Appendix 5
**Reporting biases**	21	Present assessments of risk of bias due to missing results (arising from reporting biases) for each synthesis assessed.	Not applicable
**Certainty of evidence**	22	Present assessments of certainty (or confidence) in the body of evidence for each outcome assessed.	Results
**DISCUSSION**			
**Discussion**	23a	Provide a general interpretation of the results in the context of other evidence.	Discussion
	23b	Discuss any limitations of the evidence included in the review.	Discussion
	23c	Discuss any limitations of the review processes used.	Discussion
	23d	Discuss implications of the results for practice, policy, and future research.	Discussion
**OTHER INFORMATION**			
**Registration and protocol**	24a	Provide registration information for the review, including register name and registration number, or state that the review was not registered.	Methods
	24b	Indicate where the review protocol can be accessed, or state that a protocol was not prepared.	Methods
	24c	Describe and explain any amendments to information provided at registration or in the protocol.	Not applicable
**Support**	25	Describe sources of financial or non-financial support for the review, and the role of the funders or sponsors in the review.	Abstract
**Competing interests**	26	Declare any competing interests of review authors.	Abstract
**Availability of data, code and other materials**	27	Report which of the following are publicly available and where they can be found: template data collection forms; data extracted from included studies; data used for all analyses; analytic code; any other materials used in the review.	Methods

Table 2.2.2. PRISMA abstract checklist

Topic	No.	Summary	Reported?
**Title**	1	Identify the report as a systematic review.	Yes
**BACKGROUND**			
**Objectives**	2	Provide an explicit statement of the main objective(s) or question(s) the review addresses.	Yes
**METHODS**			

**Eligibility criteria**	3	Specify the inclusion and exclusion criteria for the review.	Yes
**Information sources**	4	Specify the information sources (e.g. databases, registers) used to identify studies and the date when each was last searched.	Yes
**Risk of bias**	5	Specify the methods used to assess risk of bias in the included studies.	Yes
**Synthesis of results**	6	Specify the methods used to present and synthesize results.	Yes
**RESULTS**			
**Included studies**	7	Give the total number of included studies and participants and summarise relevant characteristics of studies.	Yes
**Synthesis of results**	8	Present results for main outcomes, preferably indicating the number of included studies and participants for each. If meta-analysis was done, report the summary estimate and confidence/credible interval. If comparing groups, indicate the direction of the effect (i.e. which group is favoured).	Yes
**DISCUSSION**			
**Limitations of evidence**	9	Provide a brief summary of the limitations of the evidence included in the review (e.g. study risk of bias, inconsistency and imprecision).	Yes
**Interpretation**	10	Provide a general interpretation of the results and important implications.	Yes
**OTHER**			
**Funding**	11	Specify the primary source of funding for the review.	Yes
**Registration**	12	Provide the register name and registration number.	Yes

2.3. Search strategy by data source

Table 2.3. Search strategy by data source used in the 2024 updated systematic review

Data source	Search strategy
**PubMed (National Center for Biotechnology Information)**	(({"HIV Infections"[Mesh] OR "HIV"[Mesh] OR "Acquired Immunodeficiency Syndrome"[Mesh] OR "Antiretroviral Therapy, Highly Active"[Mesh] OR "Anti-HIV Agents"[Mesh] OR "Anti-Retroviral Agents"[Mesh] OR "human immunodeficiency virus"[ti] OR "human immunedeficiency virus"[ti] OR "human immuno deficiency virus"[ti] OR "human immune deficiency virus"[ti] OR HIV[ti] OR HIVl[ti] OR HIV2[ti] OR "acquired immunodeficiency syndrome"[ti] OR "acquired immunedeficiency syndrome"[ti] OR "acquired immunodeficiency syndrome"[ti] OR "acquired immune deficiency syndrome"[ti] OR antiretroviral*[ti] OR "anti retroviral"[ti]) **AND** {"Pregnancy"[Mesh] OR "Pregnancy Complications, Infectious"[Mesh] OR "Pregnant Women"[Mesh] OR "Delivery, Obstetric"[Mesh] OR "Peripartum Period"[Mesh] OR "Postpartum Period"[Mesh] OR "Breast Feeding"[Mesh] OR "Infectious Disease Transmission, Vertical"[Mesh] OR PMTCT[tiab] OR MTCT[tiab] OR "mother to child"[tiab] OR "parent to child"[tiab] OR vertical*[tiab] OR intrauterine[tiab] OR "intra uterine"[tiab] OR intrapartum[tiab] OR "intra partum"[tiab] OR pregnant[tiab] OR pregnancy[tiab] OR prenatal*(tiab] OR "pre natal*"(tiab] OR antenatal*(tiab] OR "ante natal*"(tiab] OR perinatal*(tiab] OR "peri natal*"(tiab] OR puerperium[tiab] OR postnatal*[tiab] OR "post natal*"[tiab] OR postpartum[tiab] OR "post partum"[tiab] OR peripartum[tiab] OR "peri partum"[tiab] OR "in utero"[tiab] OR fetomaternal*[tiab] OR "feto maternal*"[tiab] OR "maternal fetal"[tiab] OR fetus*[tiab] OR foetus*[tiab] OR fetal*[tiab] OR foetal*[tiab] OR neonat*[tiab] OR breastfeed*[tiab] OR "breast feeding"[tiab] OR "breast fed"[tiab] OR breastmilk[tiab] OR "breast milk"[tiab] OR delivery[tiab] OR birth[tiab]) **AND** {"Infant"[Mesh] OR "Infant, Newborn"[Mesh] OR "Child"[Mesh] OR infant[tiab] OR infants[tiab] OR infancy[tiab] OR newborn*(tiab] OR "new born"[tiab] OR neonat*(tiab] OR child*(tiab] OR baby[tiab] OR babies[tiab]) **AND** {"Infectious Disease Transmission, Vertical"[Mesh] OR transmit*[tiab] OR transmission*[tiab] OR infection* OR infected)) **OR** (("HIV Infections"[Mesh] OR "HIV infection*"[tiab] OR HIV[ti]) **AND** {"Pregnancy Complications, Infectious"[Mesh] OR "Infectious Disease Transmission, Vertical"[Mesh] OR "vertical transmission"[tiab:~3] OR "vertical infection"[tiab:~3] OR "vertical infections"[tiab:~3] OR "mother to child" OR "parent to child" OR MTCT OR PMTCT OR "perinatal transmission"[tiab:~3] OR "perinatal infection"[tiab:~3] OR "perinatally acquired"[tiab:~3]))) **AND** {"2018"[Date - Publication] : "3000"[Date - Publication]) **AND** "English"[la] **NOT** (("Animals"[Mesh] OR macaque*[tiab]) NOT "Humans"[Mesh]) **NOT** (("Cross-Sectional Studies"[Mesh] OR "editorial"[Publication Type] OR "letter"[Publication Type] OR "comment"[Publication Type] OR "news"[Publication Type] OR "Case Reports" [Publication Type] OR "Case Reports as Topic"[Mesh]) NOT ("Systematic Review" [Publication Type] OR "Meta-Analysis" [Publication Type]))
**Embase (Elsevier)**	((('Human immunodeficiency virus infection'/de OR 'acquired immune deficiency syndrome'/de OR 'AIDS related complex'/de OR 'acute HIV infection'/de OR 'Human immunodeficiency virus 1 infection'/de OR 'Human immunodeficiency virus 2 infection'/de OR 'AIDS related complex'/de OR 'human immunodeficiency virus'/exp OR 'highly active antiretroviral therapy'/exp OR 'anti human immunodeficiency virus agent'/de OR 'antiretrovirus agent'/de OR 'human immunodeficiency virus':ti OR 'human immunedeficiency virus':ti OR 'human

	immunodeficiency virus':ti OR 'human immune deficiency virus':ti OR 'hiv':ti OR 'hivl':ti OR 'hiv2':ti OR 'acquired immunodeficiency syndrome':ti OR 'acquired immunedeficiency syndrome':ti OR 'acquired immuno deficiency syndrome':ti OR 'acquired immune deficiency syndrome':ti OR 'antiretroviral*':ti OR 'anti retroviral':ti) **AND** ('pregnancy'/exp OR 'infectious pregnancy complications'/exp OR 'pregnant woman'/exp OR 'obstetric delivery'/exp OR 'perinatal period'/exp OR 'puerperium'/exp OR 'breast feeding'/exp OR 'vertical transmission'/exp OR 'pmtct':ti,ab,kw OR 'mtct':ti,ab,kw OR 'mother to child':ti,ab,kw OR 'parent to child':ti,ab,kw OR 'vertical*':ti,ab,kw OR 'intrauterine':ti,ab,kw OR 'intra uterine':ti,ab,kw OR 'intrapartum':ti,ab,kw OR 'intra partum':ti,ab,kw OR 'pregnant':ti,ab,kw OR 'pregnancy':ti,ab,kw OR 'prenatal*':ti,ab,kw OR 'pre natal*':ti,ab,kw OR 'antenatal*':ti,ab,kw OR 'ante natal*':ti,ab,kw OR 'perinatal*':ti,ab,kw OR 'peri natal*':ti,ab,kw OR 'puerperium':ti,ab,kw OR 'postnatal*':ti,ab,kw OR 'post natal*':ti,ab,kw OR 'postpartum':ti,ab,kw OR 'post partum':ti,ab,kw OR 'peripartum':ti,ab,kw OR 'peri partum':ti,ab,kw OR 'in utero':ti,ab,kw OR 'fetomaternal*':ti,ab,kw OR 'feto maternal*':ti,ab,kw OR 'maternal fetal':ti,ab,kw OR 'fetus*':ti,ab,kw OR 'foetus*':ti,ab,kw OR 'fetal*':ti,ab,kw OR 'foetal*':ti,ab,kw OR 'neonat*':ti,ab,kw OR 'breastfeed*':ti,ab,kw OR 'breast feeding':ti,ab,kw OR 'breast fed':ti,ab,kw OR 'breastmilk':ti,ab,kw OR 'breast milk':ti,ab,kw OR 'delivery':ti,ab,kw OR 'birth':ti,ab,kw) **AND** ('infant'/exp OR 'newborn'/exp OR 'child'/exp OR 'infant':ti,ab,kw OR 'infants':ti,ab,kw OR 'infancy':ti,ab,kw OR 'newborn*':ti,ab,kw OR 'new born':ti,ab,kw OR 'neonat*':ti,ab,kw OR 'child*':ti,ab,kw OR 'baby':ti,ab,kw OR 'babies':ti,ab,kw) **AND** ('vertical transmission'/exp OR 'transmit*':ti,ab,kw OR 'transmission*':ti,ab,kw OR 'infection*' OR 'infected')) **OR** (('human immunodeficiency virus infection'/exp OR 'HIV infection*':ti,ab OR hiv:ti) **AND** ('infectious pregnancy complications'/exp OR 'vertical transmission'/exp OR (vertical NEAR/3 (transmission OR infection*)):ti,ab,kw OR 'mother to child':ti,ab,kw OR 'parent to child':ti,ab,kw OR MTCT OR PMTCT OR (perinatal* NEAR/3 (transmission OR infection OR acquired)):ti,ab,kw))) **AND** [english]/lim **AND** (2018-2024]/py **NOT** (('animal'/exp OR 'macaque*':ti,ab,kw) NOT 'human'/exp) **NOT** (('cross-sectional study'/exp OR 'editorial'/exp OR 'letter'/exp OR 'note'/exp OR 'case study'/exp OR 'case study':ti OR 'case report*':ti OR 'cross sectional':ti,ab) NOT ('systematic review'/exp OR 'systematic review (topic)'/exp OR 'meta analysis'/exp OR 'meta analysis (topic)'/exp)) **NOT** 'conference abstract'/exp
**CINAHL Complete (EBSCO)**	(((MH "HIV Infections" OR MH "HIV Seropositivity" OR MH "Acquired Immunodeficiency Syndrome" OR MH "Human Immunodeficiency Virus+" OR MH "HIV-Positive Persons+" OR MH "Antiretroviral Therapy, Highly Active" OR MH "Anti-HIV Agents+" OR MH "Anti-Retroviral Agents+" OR TI ("human immunodeficiency virus" OR "human immunedeficiency virus" OR "human immunodeficiency virus" OR "human immune deficiency virus" OR HIV OR HIVI OR HIV2 OR "acquired immunodeficiency syndrome" OR "acquired immunedeficiency syndrome" OR "acquired immunodeficiency syndrome" OR "acquired immune deficiency syndrome" OR antiretroviral* OR "anti retroviral")) **AND** (MH "Pregnancy+" OR MH "Childbirth+" OR MH "Pregnancy Complications, Infectious+" OR MH "Expectant Mothers" OR MH "Perinatal Period" OR MH "Postnatal Period+" OR MH "Breast Feeding+" OR MH "Disease Transmission, Vertical" OR TI (PMTCT OR MTCT OR "mother to child" OR "parent to child" OR vertical* OR intrauterine OR "intra uterine" OR

	intrapartum OR "intra partum" OR pregnant OR pregnancy OR prenatal* OR "pre natal*" OR antenatal* OR "ante natal*" OR perinatal* OR "peri natal*" OR puerperium OR postnatal* OR "post natal*" OR postpartum OR "post partum" OR peripartum OR "peri partum" OR "in utero" OR fetomaternal* OR "feto maternal*" OR "maternal fetal" OR fetus* OR foetus* OR fetal* OR foetal* OR neonat* OR breastfeed* OR "breast feeding" OR "breast fed" OR breastmilk OR "breast milk" OR delivery OR birth) OR AB (PMTCT OR MTCT OR "mother to child" OR "parent to child" OR vertical* OR intrauterine OR "intra uterine" OR intrapartum OR "intra partum" OR pregnant OR pregnancy OR prenatal* OR "pre natal*" OR antenatal* OR "ante natal*" OR perinatal* OR "peri natal*" OR puerperium OR postnatal* OR "post natal*" OR postpartum OR "postpartum" OR peripartum OR "peri partum" OR "in utero" OR fetomaternal* OR "feto maternal*" OR "maternal fetal" OR fetus* OR foetus* OR fetal* OR foetal* OR neonat* OR breastfeed* OR "breast feeding" OR "breast fed" OR breastmilk OR "breast milk" OR delivery OR birth)) **AND** (MH "Infant+" OR MH "Infant, Newborn+" OR MH "Child+" OR TI (infant OR infants OR infancy OR newborn* OR "new born" OR neonat* OR child* OR baby OR babies) OR AB (infant OR infants OR infancy OR newborn* OR "new born" OR neonat* OR child* OR baby OR babies)) **AND** (MH "Disease Transmission, Vertical" OR TI (transmit* OR transmission* OR infection* OR infected) OR AB (transmit* OR transmission*))) **OR** ((MH "HIV Infections" OR TI("HIV infection*" OR HIV) OR AB("HIV infection*")) **AND** (MH "Pregnancy Complications, Infectious+" OR MH "Disease Transmission, Vertical" OR (Vertical N3 (transmission OR infection*)) OR "mother to child" OR "parent to child" OR (perinatal N3 (transmission OR infection*)) OR (perinatally N3 acquired) OR MTCT OR PMTCT))) **AND** PY 2018-2024 **AND** LA "English" **NOT** ((MH "Animals") NOT (MH "Human")) **NOT** ((MH "Cross Sectional Studies" OR MH "Case Studies" OR TI ("case report" OR "case reports" OR "case series" OR "cross sectional") OR PT (commentary OR editorial OR letter)) NOT PT ("systematic review" OR "meta analysis")) NOT PT abstract
**Global Health (EBSCO)**	Limit: Publication Year 2018-2024<br>((((DE "HIV infections" OR DE "HIV-1 infections" OR DE "HIV-2 infections" OR DE "human immunodeficiency viruses" OR DE "Human immunodeficiency virus 1" OR DE "Human immunodeficiency virus 2" OR DE "acquired immune deficiency syndrome" OR DE "antiretroviral agents" OR DE "reverse transcriptase inhibitors" OR TI ("human immunodeficiency virus" OR "human immunedeficiency virus" OR "human immunodeficiency virus" OR "human immune deficiency virus" OR HIV OR HIVI OR HIV2 OR "acquired immunodeficiency syndrome" OR "acquired immunedeficiency syndrome" OR "acquired immunodeficiency syndrome" OR "acquired immune deficiency syndrome" OR antiretroviral* OR "anti retroviral")) **AND** (DE "pregnancy" OR DE "birth" OR DE "childbirth" OR DE "postpartum period" OR DE "pregnancy complications" OR DE "parturition" OR DE "prenatal period" OR DE "prepartum period" OR DE "puerperium" OR DE "fetus" OR DE "breast feeding" OR DE "human milk" OR DE "vertical transmission" OR DE "maternal transmission" OR TI (PMTCT OR MTCT OR "mother to child" OR "parent to child" OR vertical* OR intrauterine OR "intra uterine" OR intrapartum OR "intra partum" OR pregnant OR pregnancy OR prenatal* OR "pre natal*" OR antenatal*

	OR "ante natal*" OR perinatal* OR "perinatal*" OR puerperium OR postnatal* OR "post natal*" OR postpartum OR "postpartum" OR peripartum OR "peri partum" OR "in utero" OR fetomaternal* OR "feto maternal*" OR "maternal fetal" OR fetus* OR foetus* OR fetal* OR foetal* OR neonat* OR breastfeed* OR "breast feeding" OR "breast fed" OR breastmilk OR "breast milk" OR delivery OR birth) OR AB (PMTCT OR MTCT OR "mother to child" OR "parent to child" OR vertical* OR intrauterine OR "intra uterine" OR intrapartum OR "intra partum" OR pregnant OR pregnancy OR prenatal* OR "pre natal*" OR antenatal* OR "ante natal*" OR perinatal* OR "peri natal*" OR puerperium OR postnatal* OR "post natal*" OR postpartum OR "postpartum" OR peripartum OR "peri partum" OR "in utero" OR fetomaternal* OR "feto maternal*" OR "maternal fetal" OR fetus* OR foetus* OR fetal* OR foetal* OR neonat* OR breastfeed* OR "breast feeding" OR "breast fed" OR breastmilk OR "breast milk" OR delivery OR birth)) **AND** (DE "children" OR DE "preschool children" OR DE "school children" OR DE "infants" OR DE "neonates" OR DE "neonates" OR TI (infant OR infants OR infancy OR newborn* OR "new born" OR neonat* OR child* OR baby OR babies) OR AB (infant OR infants OR infancy OR newborn* OR "new born" OR neonat* OR child* OR baby OR babies)) **AND** (DE "vertical transmission" OR DE "maternal transmission" OR TI (transmit* OR transmission* OR infection* OR infected) OR AB (transmit* OR transmission*))) **OR** ((DE "HIV infections" OR DE "HIV-1 infections" OR DE "HIV-2 infections" OR TI("HIV infection*" OR HIV) OR AB("HIV infection*")) **AND** (DE "vertical transmission" OR DE "maternal transmission" OR (Vertical N3 (transmission OR infection)) OR "mother to child" OR "parent to child" OR (perinatal N3 (transmission OR infection)) OR (perinatally N3 acquired) OR MTCT OR PMTCT))) **AND** LA "English" **NOT** ((DE "Animals" OR DE "Laboratory Animals") NOT DE "Hominidae") **NOT** ((TI "cross sectional" OR AB "cross sectional" OR ZT "editorial" OR ZT "letter" OR DE "case reports" OR TI "case report" OR TI "case reports" OR TI "case series") NOT (ZU "systematic reviews" OR ZU "meta-analysis"))
**Cochrane CENTRAL (Wiley)**	Limit - language: English<br><br>ID Search Hits<br>#1 MeSH descriptor: [HIV Infections] explode all trees 17667<br>#2 MeSH descriptor: [HIV] explode all trees 4211<br>#3 MeSH descriptor: [Anti-Retroviral Agents] explode all trees 6112<br>#4 MeSH descriptor: [Anti-HIV Agents] explode all trees 5128<br>#5 MeSH descriptor: [Antiretroviral Therapy, Highly Active] explode all trees 1626<br>#6 (HIV NEXT infection*) OR "human immunodeficiency virus" OR "human immunedeficiency virus" OR "human immune deficiency virus" OR "human immunodeficiency virus" OR HIV OR HIVI OR HIV2 OR "acquired immunodeficiency syndrome" OR "acquired immunedeficiency syndrome" OR "acquired immune deficiency syndrome" OR "acquired immunodeficiency syndrome" OR antiretroviral* OR "anti retroviral" 34501

	#7 MeSH descriptor: [Pregnancy] explode all trees 33699<br>#8 MeSH descriptor: [Pregnancy Complications, Infectious] explode all<br>trees 1616<br>#9 MeSH descriptor: [Pregnant Women] explode all trees 988<br>#10 MeSH descriptor: [Delivery, Obstetric) explode all trees 7441<br>#11 MeSH descriptor: [Peripartum Period] explode all trees 47<br>#12 MeSH descriptor: [Postpartum Period) explode all trees 2754<br>#13 MeSH descriptor: [Breast Feeding] explode all trees 2878<br>#14 MeSH descriptor: [Infectious Disease Transmission, Vertical] explode<br>all trees 842<br>#15 PMTCT OR MTCT OR "mother to child" OR "parent to child" OR vertical* OR intrauterine OR "intra uterine" OR intrapartum OR "intra partum" OR pregnant OR pregnancy OR prenatal* OR (pre NEXT natal*) OR<br>antenatal* OR (ante NEXT natal*) OR perinatal* OR (peri NEXT natal*) OR<br>puerperium OR postnatal* OR (post NEXT natal*) OR postpartum OR "post<br>partum" OR peripartum OR "peri partum" OR "in utero" OR fetomaternal* OR<br>(feto NEXT maternal*) OR "maternal fetal" OR fetus* OR foetus* OR fetal* OR<br>foetal* OR neonat* OR breastfeed* OR "breast feeding" OR "breast fed" OR<br>breastmilk OR "breast milk" OR delivery OR birth 186277<br>#16 MeSH descriptor: [Infant] explode all trees 45750<br>#17 MeSH descriptor: [Child) explode all trees 81197<br>#18 infant OR infants OR infancy OR newborn* OR "new born" OR neonat* OR child* OR baby OR babies 265502<br>#19 transmit* OR transmission* OR infection* OR infected 167225<br>#20 (#1 OR #2 OR #3 OR #4 OR #5 OR #6) AND (#7 OR #8 OR #9 OR #10 OR<br>#11 OR #12 OR #13 OR #14 OR #15) AND (#16 OR #17 OR #18) AND (#14 OR<br>#19) 2943<br>#21 (HIV NEXT infection*) OR HIV 33237<br>#22 (vertical NEAR/3 transmission) OR "mother to child" OR "parent to child" OR MTCT OR PMTCT OR (perinatal NEAR/3 transmission) OR (perinatal NEAR/3 infection*) OR (perinatally NEAR/3 acquired) 1845<br>#23 (#1 OR #21) AND #22 1327<br>#24 #20 OR #23 with Publication Year from 2018 to 2024, in Trials 773
**Global Index Medicus (World Health Organization)**	(tw:(hiv OR "human immunodeficiency virus"))<br>AND<br>(tw:((pregnan* OR childbirth OR breast* OR perinatal* OR prenatal* OR utero<br>OR vertical* OR "mother to child")))<br>AND<br>(tw:(transmit* OR transmission))<br>AND<br>La:("en")<br>AND<br>Year_cluster:[2018 to 2024]

2.4. Variables extracted in systematic review

The following variables were extracted from all included studies:
Study details:

- Author name(s)
- Study title
- Journal
- Publication year
- Geographic region(s) covered (i.e. country or countries)
- Dates of data collection
- Study population(s)
- Total population size

Vertical transmission details:

- Total N of pregnant women or breastfeeding mothers living with HIV
- Total N of infants born to mothers living with HIV, who were tested for HIV
- N of infants who tested HIV-positive
- Events of vertical HIV transmission
- Timing of transmission (perinatal or during breastfeeding)
- Age of child at testing
- Maternal prophylaxis

When available:

- Maternal ART regimens
- Timing of maternal ART initiation
- Infant treatment and prophylaxis
- Maternal viral load or viral suppression information
    a. Timing of viral load test
    b. In viral load suppression, threshold for viral load suppression
- Maternal CD4 count information at baseline
- Infant feeding patterns (breastfed or formula fed, including duration in months)

2.5. Perinatal transmission probability definition

Perinatal transmission was defined as transmission that occurs before six weeks (1.5 months) after birth. We included studies with both breastfeeding and formula-feeding populations. To model perinatal VT probability, we extracted the number of infections identified before six weeks postpartum and the number of HIV exposed infants.

$$PVT = \frac{HPI_{1.5\,months}}{HEI}$$

Equation 2.2

Perinatal VT (*PVT*) probability was then calculated as the ratio of HIV positive infants ($HPI_{1.5\,months}$) to HIV exposed infants (*HEI*) as shown in Equation 2.2.

2.6. Monthly breastfeeding transmission probability definition

Breastfeeding transmission was calculated as a monthly transmission probability and considered any vertical transmission that occurred in breastfeeding populations for infants after six weeks of age. The monthly breastfeeding transmission probability (*BFVT*) was then calculated according to Equation 2.3.

$$BFVT = \frac{HPI_{BF_{End}} - HPI_{1.5\,months}}{(HEI - HPI_{1.5\,months}) * (BF_{End} - 1.5)}$$

Equation 2.3

The numerator represents the number of infections that occurred between the end of breastfeeding and the end of the perinatal period as the difference between the number of HIV positive infants at the end of breastfeeding ($HPI_{BF_{End}}$) and at 1.5 months ($HPI_{1.5\,months}$). The denominator represents the number of HIV exposed (*HEI*) but uninfected infants during this period (minus $HPI_{1.5\,months}$) and the number of months between the perinatal period (1.5) and the end of breastfeeding (BF_{End}).

3. Meta-regression model estimates

3.1 Regression tables for all models

Table 3.1.1. Regression table for models one, two, three, and four used to estimate VT probabilities compatible with Spectrum-AIM

Model one: VT probability among women not receiving PVT				
**Covariate**	**Estimate (logit)**	**95% confidence interval**	**Estimate (odds ratio)**	**95% confidence interval**
**Intercept**	-1.61	(-1.82, -1.41)	0.20	(0.16, 0.24)
**CD4 midpoint** **(per 100 cells increase, centered on CD4 = 500 mm^3)**	-0.23	(-0.28, -0.18)	0.80	(0.75, 0.84)
**Perinatal transmission** **(Reference)**	0.00	(Reference)	1.00	(Reference)
**Breastfeeding transmission**	-3.23	(-3.84, -2.61)	0.04	(0.02, 0.07)
**Interaction between CD4 midpoint and breastfeeding transmission**	0.16	(-0.22, 0.54)	1.18	(0.80, 1.72)

Model two: VT probability among maternal seroconversion or short-course PVT					
**Covariate**		**Estimate (logit)**	**95% confidence interval**	**Estimate (odds)**	**95% confidence interval**
**Transmission timing**	**Category**				
**Perinatal**	**Infection**	-1.51	(-1.92, -1.09)	0.221	(0.147, 0.337)
	**SDNVP**	-2.40	(-2.76, -2.06)	0.091	(0.063, 0.128)
	**Dual ARV**	-3.44	(-3.85, -3.01)	0.032	(0.021, 0.049)
	**Option A**	-3.43	(-3.73, -3.13)	0.032	(0.024, 0.044)
	**Option B**	-4.02	(-4.27, -3.77)	0.018	(0.014, 0.023)
**Breastfeeding (monthly)**	**Infection**	-0.93	(-1.34, -0.52)	0.395	(0.263, 0.592)
	**SDNVP, <350**	-4.71	(-6.43, -3.07)	0.009	(0.002, 0.047)
	**SDNVP, >350**	-5.64	(-7.40, -3.92)	0.004	(0.001, 0.020)
	**Dual ARV**	-6.21	(-7.49, -4.89)	0.002	(0.001, 0.007)
	**Option A**	-6.18	(-7.37, -5.07)	0.002	(0.001, 0.006)
	**Option B**	-6.58	(-7.32, -5.81)	0.001	(0.001, 0.003)

Model three: Perinatal transmission probability among women receiving ART by timing of initiation				
**Covariate**	**Estimate (logit)**	**95% confidence interval**	**Estimate (odds ratio)**	**95% confidence interval**
**Intercept**	-4.55	(-4.79, -4.32)	0.011	(0.008, 0.013)
**Weeks on ART before delivery** **(centered on 20 weeks)**	-0.06	(-0.07, -0.04)	0.944	(0.930, 0.958)
**Late ART initiation (<4 weeks before delivery)**	0.68	(-0.05, 1.45)	1.974	(0.952, 4.265)

Model four: Monthly breastfeeding transmission among women receiving lifelong ART				
**Covariate**	**Estimate (logit)**	**95% confidence interval**	**Estimate (odds ratio)**	**95% confidence interval**
**Intercept** **(on ART preconception)**	-8.70	(-10.13, -7.40)	0.000	(0.000, 0.001)
**ART started during pregnancy**	2.06	(0.63, 3.56)	7.813	(1.878, 35.245)

Table 3.1.2. Regression table for model three with fixed effects for ART regimen class

Covariate	Estimate (logit)	95% confidence interval	Estimate (odds ratio)	95% confidence interval
**Intercept**	-4.45	(-4.71, -4.19)	0.012	(0.009, 0.015)
**Weeks on ART before delivery (centered on 20 weeks)**	-0.06	(-0.07, -0.04)	0.946	(0.932, 0.961)
**Late ART initiation (<4 weeks before delivery)**	0.72	(-0.05, 1.49)	2.050	(0.951, 4.418)
**ART class**				
**NNRTI (reference)**	0.00	(Reference)	1.000	(Reference)
**INSTI**	-1.01	(-1.95, -0.07)	0.364	(0.142, 0.935)
**PI**	-0.12	(-0.56, 0.32)	0.885	(0.570, 1.374)
**Miscellaneous regimens**	-0.04	(-0.67, 0.59)	0.957	(0.510, 1.795)

Table 4.1.3. Regression table for model three with fixed effects for ART regimen class and region

Covariate	Estimate (logit)	95% confidence interval	Estimate (odds ratio)	95% confidence interval
**Intercept**	-4.31	(-357, -4.05)	0.013	(0.010, 0.017)
**Weeks on ART before delivery (centered on 20 weeks)**	-0.06	(-0.07, -0.04)	0.946	(0.932, 0.961)
**Late ART initiation (<4 weeks before delivery)**	1.05	(0.31, 1.79)	2.865	(1.370, 5.992)
**ART class**				
**NNRTI (reference)**	0.00	(Reference)	1.000	(Reference)
**INSTI**	-0.74	(-1.75, 0.26)	0.475	(0.174, 1.300)
**PI**	0.07	(-0.37, 0.51)	1.076	(0.694, 1.670)
**Miscellaneous regimens**	0.22	(-0.29, 0.72)	1.242	(0.748, 2.062)
**Region**				
**Sub-Saharan Africa (reference)**	0.00	(Reference)	1.000	(Reference)
**Non Sub-Saharan Africa**	-0.58	(-0.95, -0.21)	0.560	(0.386, 0.813)
**Mixed regions**	-0.18	(-1.07, 0.72)	0.838	(0.343, 2.048)

Table 3.1.4. Regression table for viral load suppression model by ART regimen class and timing of ART initiation

Covariate	Estimate (logit)	95% confidence interval	Estimate (odds ratio)	95% confidence interval
**Intercept**	2.26	(1.42, 3.04)	9.59	(4.16, 21.01)
**ART class**				
**NNRTI (reference)**	0.00	(Reference)	1.00	(Reference)
**INSTI**	0.86	(-0.72, 2.51)	2.37	(0.48, 12.28)
**PI**	-0.92	(-1.73, -0.10)	0.40	(0.18, 0.91)
**Miscellaneous regimens**	-0.06	(-0.89, 0.76)	0.94	(0.41, 2.14)
**Later ART initiation (After 1st trimester)**	-0.98	(-1.58, -0.38)	0.37	(0.21, 0.68)
**Interaction with INSTI**	-0.18	(-1.99, 1.64)	0.84	(0.14, 5.14)
**Interaction with PI**	0.05	(-0.83, 0.94)	1.05	(0.44, 2.56)
**Interaction with miscellaneous regimens**	-0.16	(-2.51, 2.19)	0.85	(0.08, 8.98)

3.2 Study-level and pooled estimates of vertical transmission probability

Figures 3.2.1 through 3.2.15 presented extracted systematic review data as forest plots, stratified according to the VT probability categories defined by Spectrum-AIM. Data are summarised as forest plots for each stratum.

Data from studies newly identified in the 2024 systematic review are shown in red; studies whereas studies that were in the previous systematic reviews and used in this review are in black, and studies used to calculate the former VT probabilities but were excluded in this analysis are shown in grey.

Studies are summarised as weighted average of the studies used to produce the former VT used in Spectrum-AIM (‘Weighted average: former VT’) and estimates from models two and four fit to the pre-2024 systematic review VT studies (‘Modelled: former VT’). When using the data used to estimate the former VT probabilities, there was insufficient data to fit model one and model three. For each category, we give reasons for excluding studies that had been used in the former VT probabilities but are not used in this analysis.

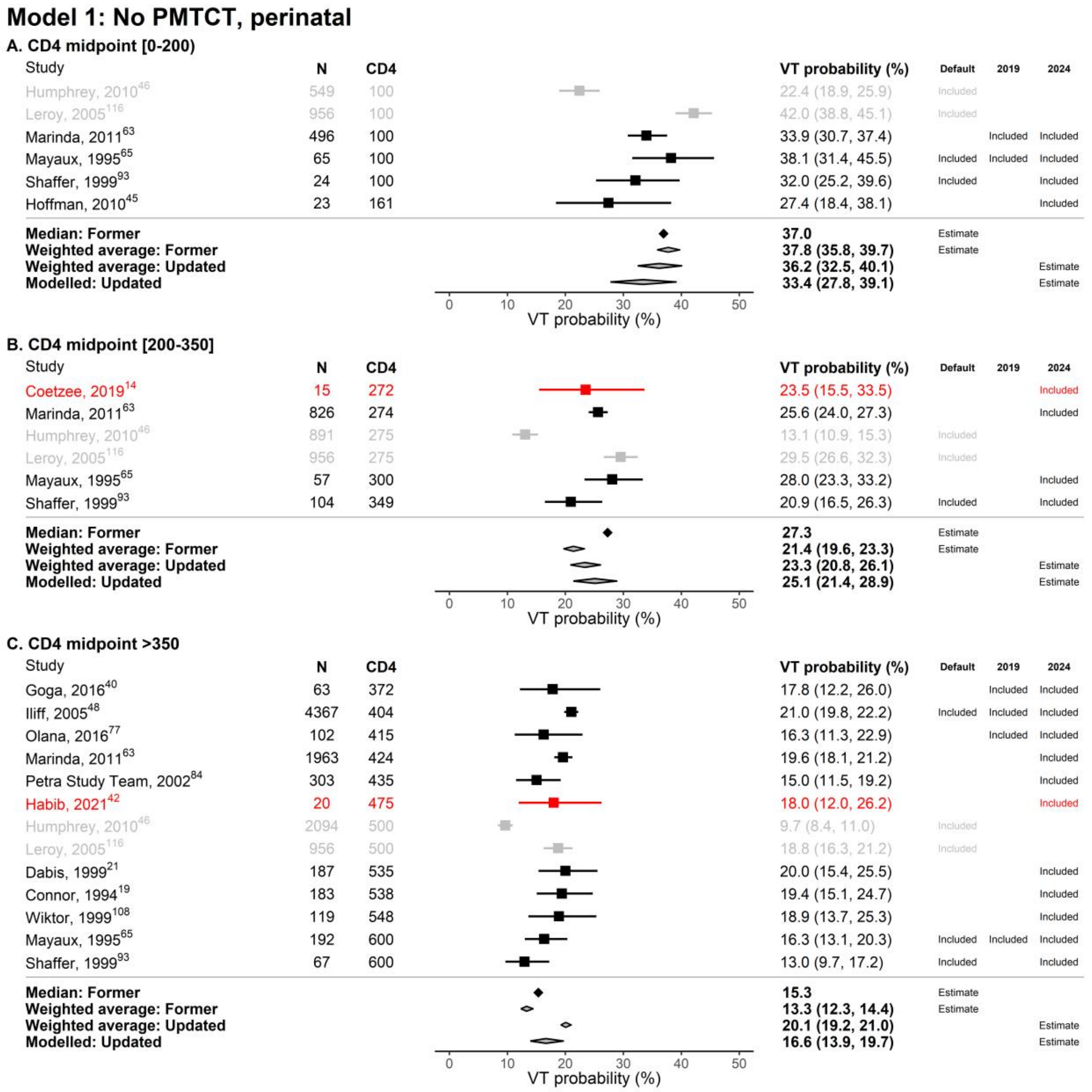

Figure 3.2.1 Pooled estimates of perinatal VT among women not receiving PVT. Results are stratified by the Spectrum-AIM defined CD4 ranges. The following pooled estimates are presented: median of studies included in the former VT probabilities ('Median: former VT'), the weighted average of studies included in the former VT probabilities ('Weighted average: former VT'), the weighted average of studies included in this analysis ('Weighted average: Updated'), and the results of the model one ('Modelled: Updated'). Studies added in the 2024 review are shown in red and studies excluded from the meta-regression are shown in grey. Humphrey 2010 was excluded as it was not a peer-reviewed study and Leroy 2005 was excluded as it was a pooled analysis using data from other included studies.

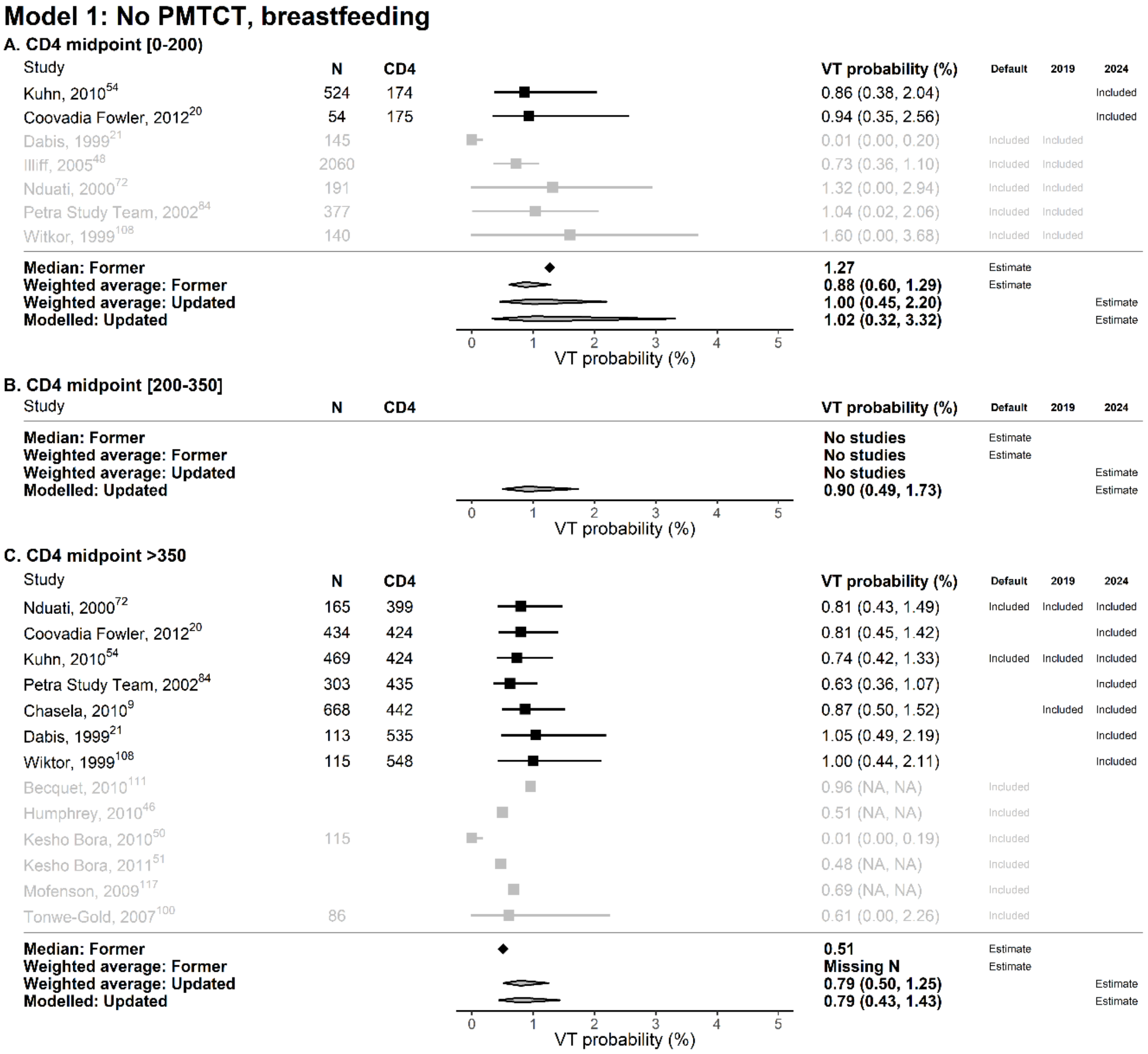

Figure 3.2.2 Pooled estimates of breastfeeding VT among women not receiving PVT.

Results are stratified by the Spectrum-AIM defined CD4 ranges. The following pooled estimates are presented: median of studies included in the former VT probabilities ('Median: former VT'), the weighted average of studies included in the former VT probabilities ('Weighted average: former VT'), the weighted average of studies included in this analysis ('Weighted average: Updated'), and the results of the model one ('Modelled: Updated'). Studies excluded from the meta-regression are shown in grey. Kesho Bora 2010, Kesho Bora 2011, and Tonwe-Gold 2007 were excluded as mothers received AZT. Becquet 2010, Humphrey 2010, and Mofenson 2009 were excluded as they were not peer-reviewed studies. Iliff 2005 was excluded as it was a pooled analysis using data from other included studies.

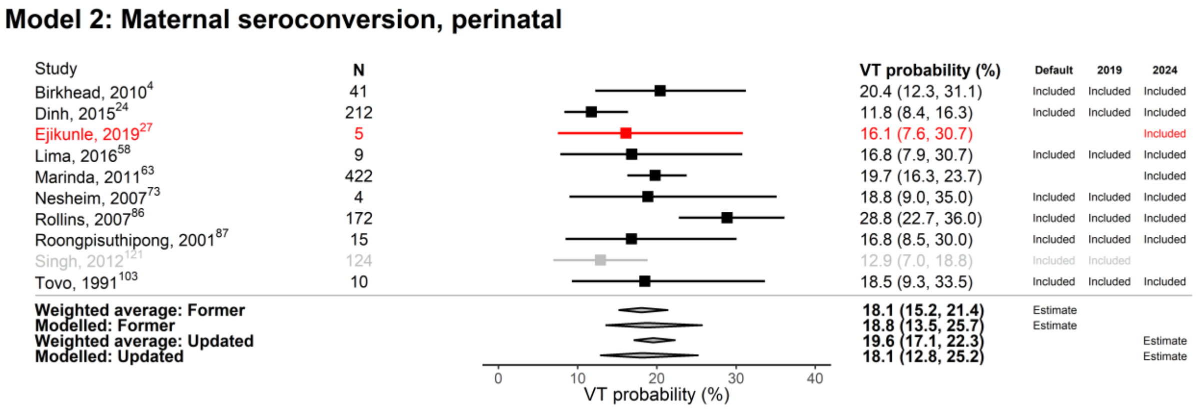

Figure 3.2.3 Pooled estimates of VT among women who seroconverted during pregnancy. The following pooled estimates are presented: the weighted average of studies included in the former VT probabilities ('Weighted average: former VT'), estimates from model two fit to the pre-2024 systematic review studies ('Modelled: former VT'), the weighted average of studies included in this analysis ('Weighted average: Updated'), and the results of the model two ('Modelled: Updated'). Studies added in the 2024 review are shown in red and studies excluded from the meta-regression are shown in grey. Singh 2012 was excluded as it was not a peer-reviewed study.

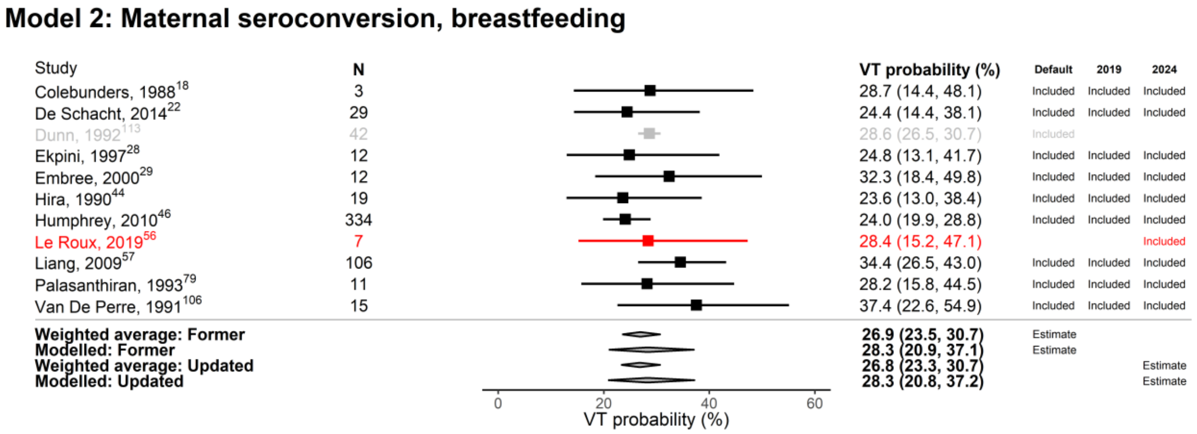

Figure 3.2.4 Pooled estimates of VT among women who seroconverted during breastfeeding. The following pooled estimates are presented: the weighted average of studies included in the former VT probabilities ('Weighted average: former VT'), estimates from model two fit to the pre-2024 systematic review studies ('Modelled: former VT'), the weighted average of studies included in this analysis ('Weighted average: Updated'), and the results of the model two ('Modelled: Updated'). Studies added in the 2024 review are shown in red and studies excluded from the meta-regression are shown in grey. Singh 2012 was excluded as it was not a peer-reviewed study. Dunn 1992 was excluded as it was a pooled analysis using data from other included studies.

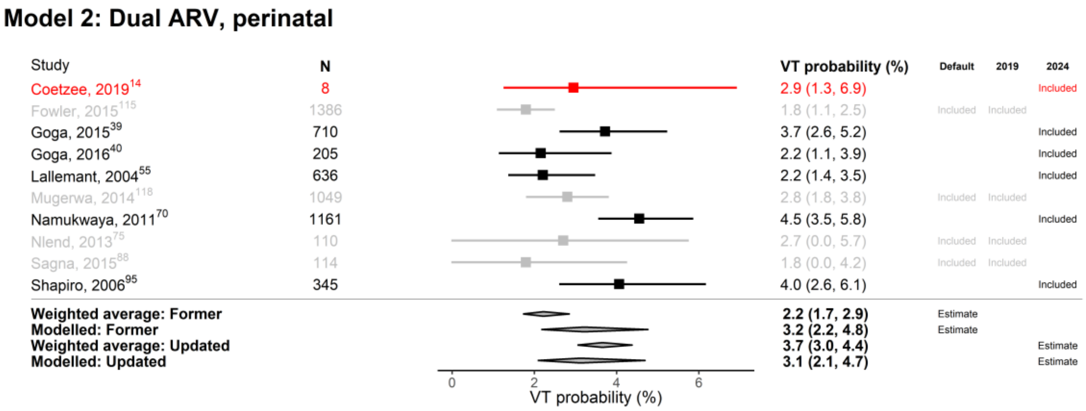

Figure 3.2.5 Pooled estimates of perinatal VT among women receiving dual ARV. The following pooled estimates are presented: the weighted average of studies included in the former VT probabilities ('Weighted

average: former VT’), estimates from model two fit to the pre-2024 systematic review studies (‘Modelled: former VT’), the weighted average of studies included in this analysis (‘Weighted average: Updated’), and the results of the model two (‘Modelled: Updated‘). Studies added in the 2024 review are shown in red and studies excluded from the meta-regression are shown in grey. Fowler 2015 and Mugerwa 2014 were excluded as they were not peer-reviewed studies and Nlend 2013 and Sagna 2015 were excluded as they excluded WLHIV with CD4 <350.

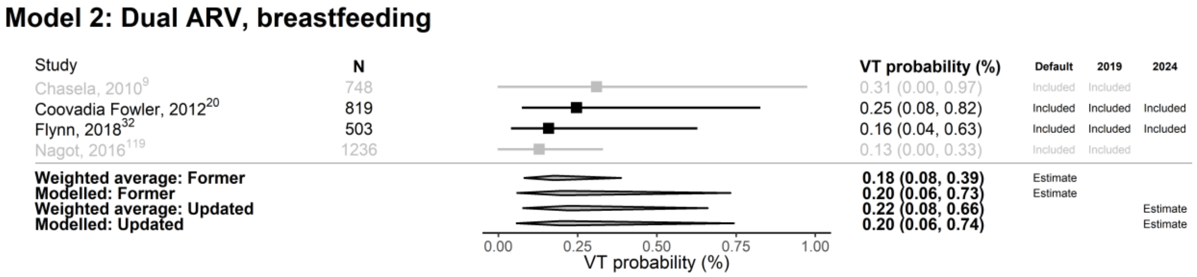

Figure 3.2.6 Pooled estimates of breastfeeding VT among women receiving dual ARV. The following pooled estimates are presented: the weighted average of studies included in the former VT probabilities (‘Weighted average: former VT’), estimates from model two fit to the pre-2024 systematic review studies (‘Modelled: former VT’), the weighted average of studies included in this analysis (‘Weighted average: Updated’), and the results of the model two (‘Modelled: Updated‘). Studies excluded from the meta-regression are shown in grey. Chasela 2010 and Nagot 2016 were excluded as they excluded WLHIV with CD4 < 350.

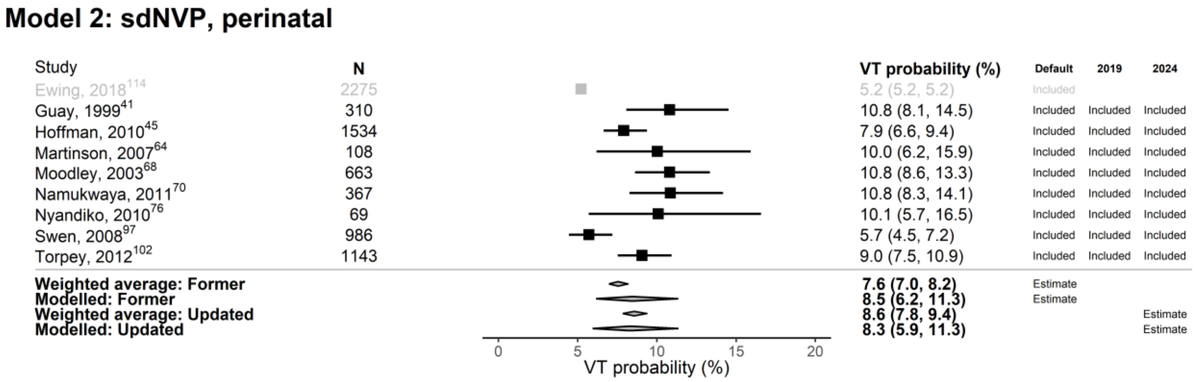

Figure 3.2.7 Pooled estimates of perinatal VT among women receiving single dose nevirapine. The following pooled estimates are presented: the weighted average of studies included in the former VT probabilities (‘Weighted average: former VT’), estimates from model two fit to the pre-2024 systematic review studies (‘Modelled: former VT’), the weighted average of studies included in this analysis (‘Weighted average: Updated’), and the results of the model two (‘Modelled: Updated‘). Studies excluded from the meta-regression are shown in grey. Ewing 2018 was excluded as it was not a peer-reviewed study.

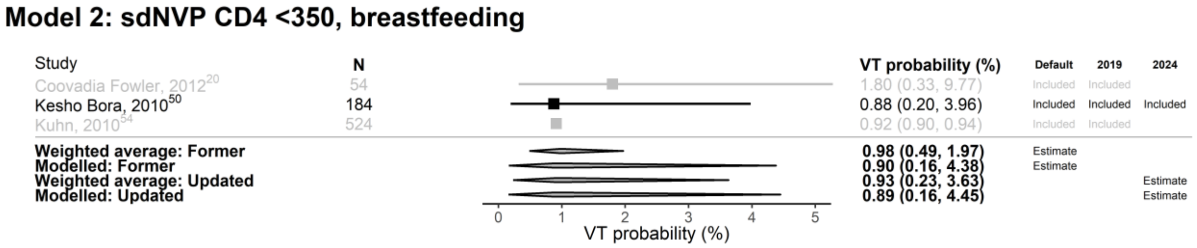

Figure 3.2.8 Pooled estimates of breastfeeding VT among women with CD4 < 350 receiving single dose nevirapine. The following pooled estimates are presented: the weighted average of studies included in the former VT probabilities (‘Weighted average: former VT’), estimates from model two fit to the pre-2024 systematic review studies (‘Modelled: former VT’), the weighted average of studies included in this analysis (‘Weighted average: Updated’), and the results of the model two (‘Modelled: Updated‘). Studies excluded from the meta-regression are shown in grey. Kuhn 2010 was excluded as WLHIV were receiving ART.

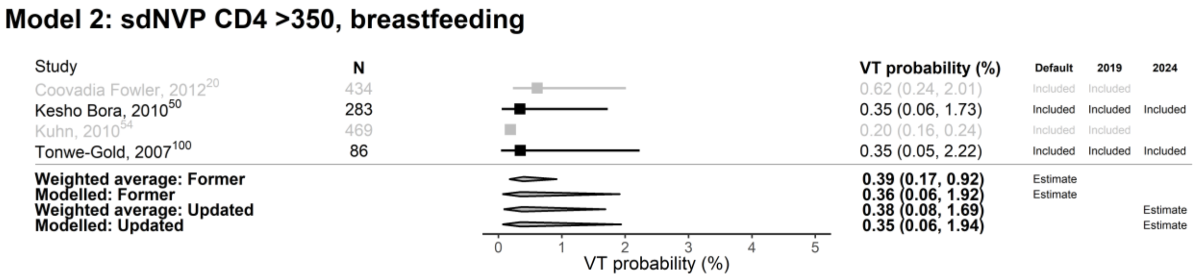

Figure 3.2.9 Pooled estimates of breastfeeding VT among women with CD4 >350 receiving single dose nevirapine. The following pooled estimates are presented: the weighted average of studies included in the former VT probabilities ('Weighted average: former VT'), estimates from model two fit to the pre-2024 systematic review studies ('Modelled: former VT'), the weighted average of studies included in this analysis ('Weighted average: Updated'), and the results of the model two ('Modelled: Updated'). Studies excluded from the meta-regression are shown in grey. Kuhn 2010 was excluded as WLHIV were receiving ART.

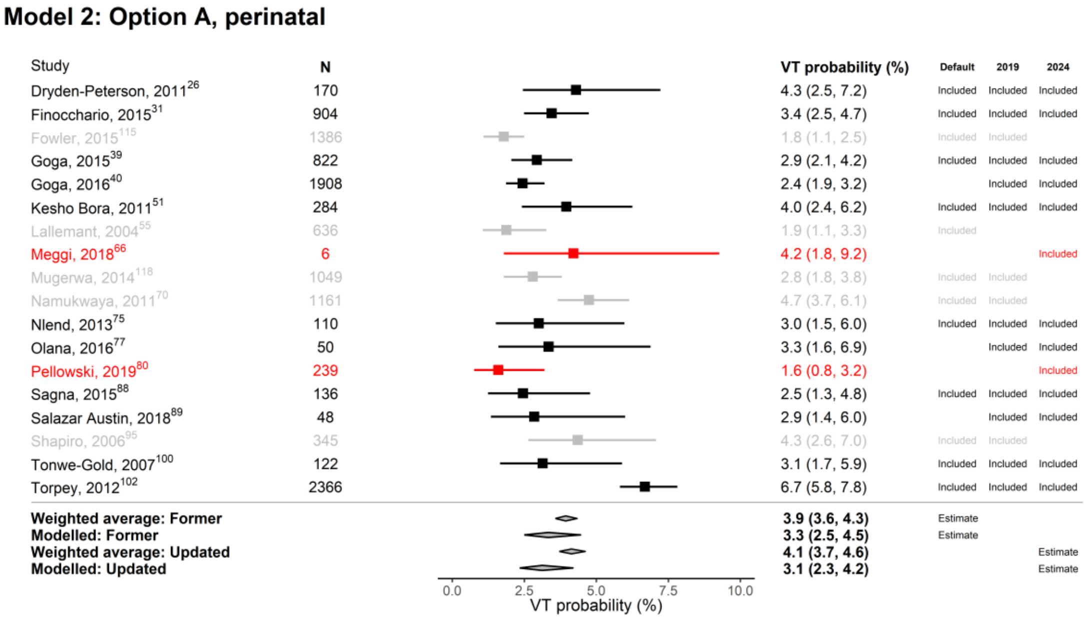

Figure 3.2.10 Pooled estimates of perinatal VT among women receiving Option A. The following pooled estimates are presented: the weighted average of studies included in the former VT probabilities ('Weighted average: former VT'), estimates from model two fit to the pre-2024 systematic review studies ('Modelled: former VT'), the weighted average of studies included in this analysis ('Weighted average: Updated'), and the results of the model two ('Modelled: Updated'). Studies added in the 2024 review are shown in red and studies excluded from the meta-regression are shown in grey. Fowler 2015 and Mugerwa 2014 were excluded as they were not peer-reviewed studies, all other studies that were excluded had women who initiated AZT at 28 weeks rather than 14 weeks.

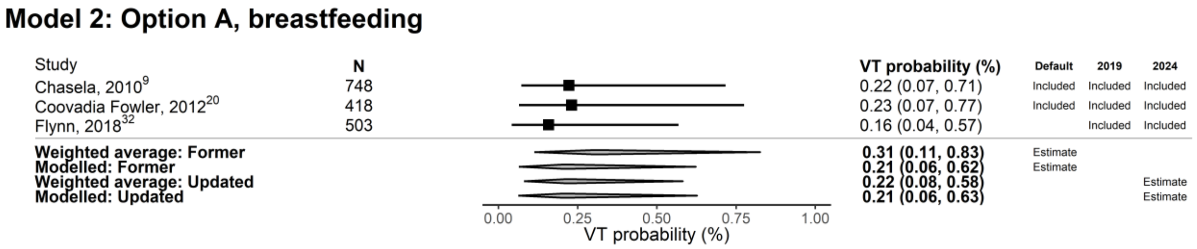

Figure 3.2.11 Pooled estimates of breastfeeding VT among women receiving Option A. The following pooled estimates are presented: the weighted average of studies included in the former VT probabilities ('Weighted average: former VT'), estimates from model two fit to the pre-2024 systematic review studies ('Modelled: former VT'), the weighted average of studies included in this analysis ('Weighted average: Updated'), and the results of the model two ('Modelled: Updated').

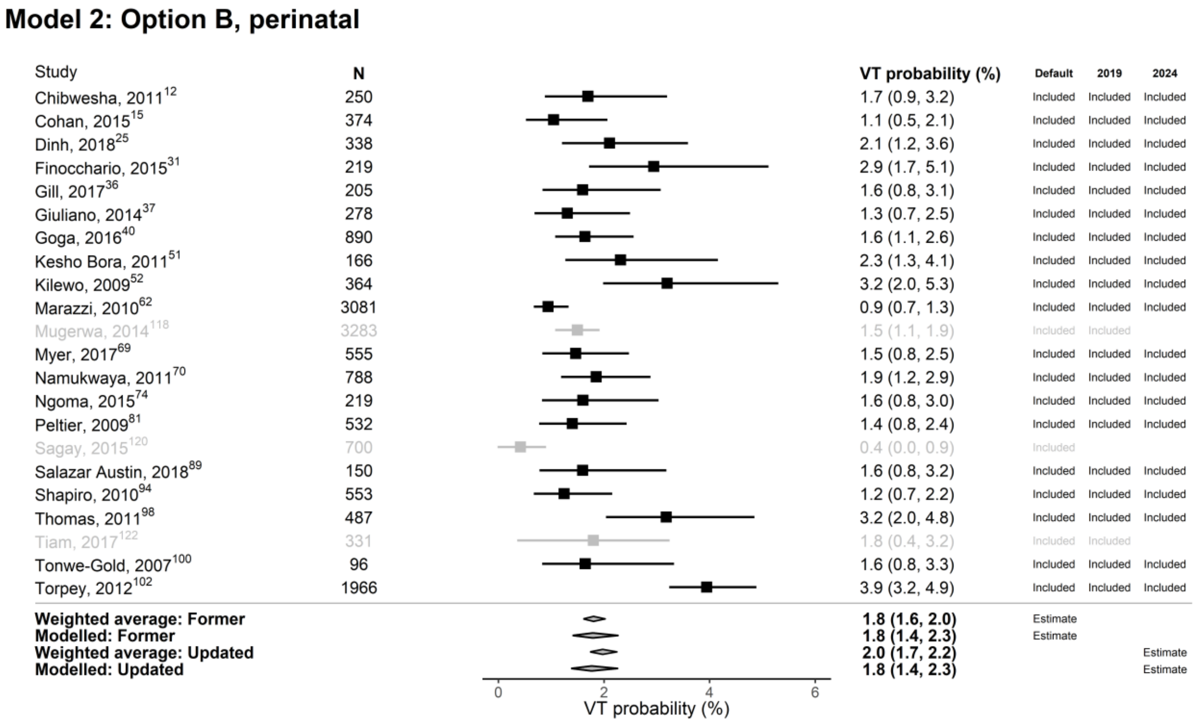

Figure 3.2.12 Pooled estimates of perinatal VT among women receiving Option B. The following pooled estimates are presented: the weighted average of studies included in the former VT probabilities ('Weighted average: former VT'), estimates from model two fit to the pre-2024 systematic review studies ('Modelled: former VT'), the weighted average of studies included in this analysis ('Weighted average: Updated'), and the results of the model two ('Modelled: Updated'). Studies excluded from the meta-regression are shown in grey. Mugerwa 2014 and Tiam 2017 were excluded as they were not peer-reviewed studies. Sagay 2015 was excluded as we could not determine the timing of the HIV test.

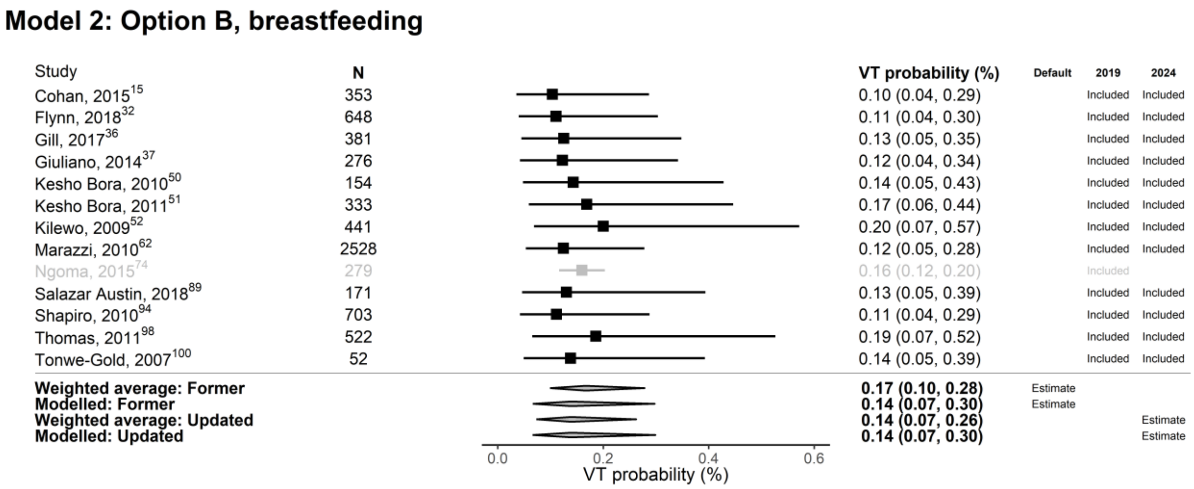

Figure 3.2.13 Pooled estimates of breastfeeding VT among women receiving Option B. The following pooled estimates are presented: the weighted average of studies included in the former VT probabilities ('Weighted average: former VT'), estimates from model two fit to the pre-2024 systematic review studies ('Modelled: former VT'), the weighted average of studies included in this analysis ('Weighted average: Updated'), and the results of the model two ('Modelled: Updated'). Studies excluded from the meta-regression are shown in grey. Ngoma 2015 was excluded as it did not restrict to women with CD4 >350.

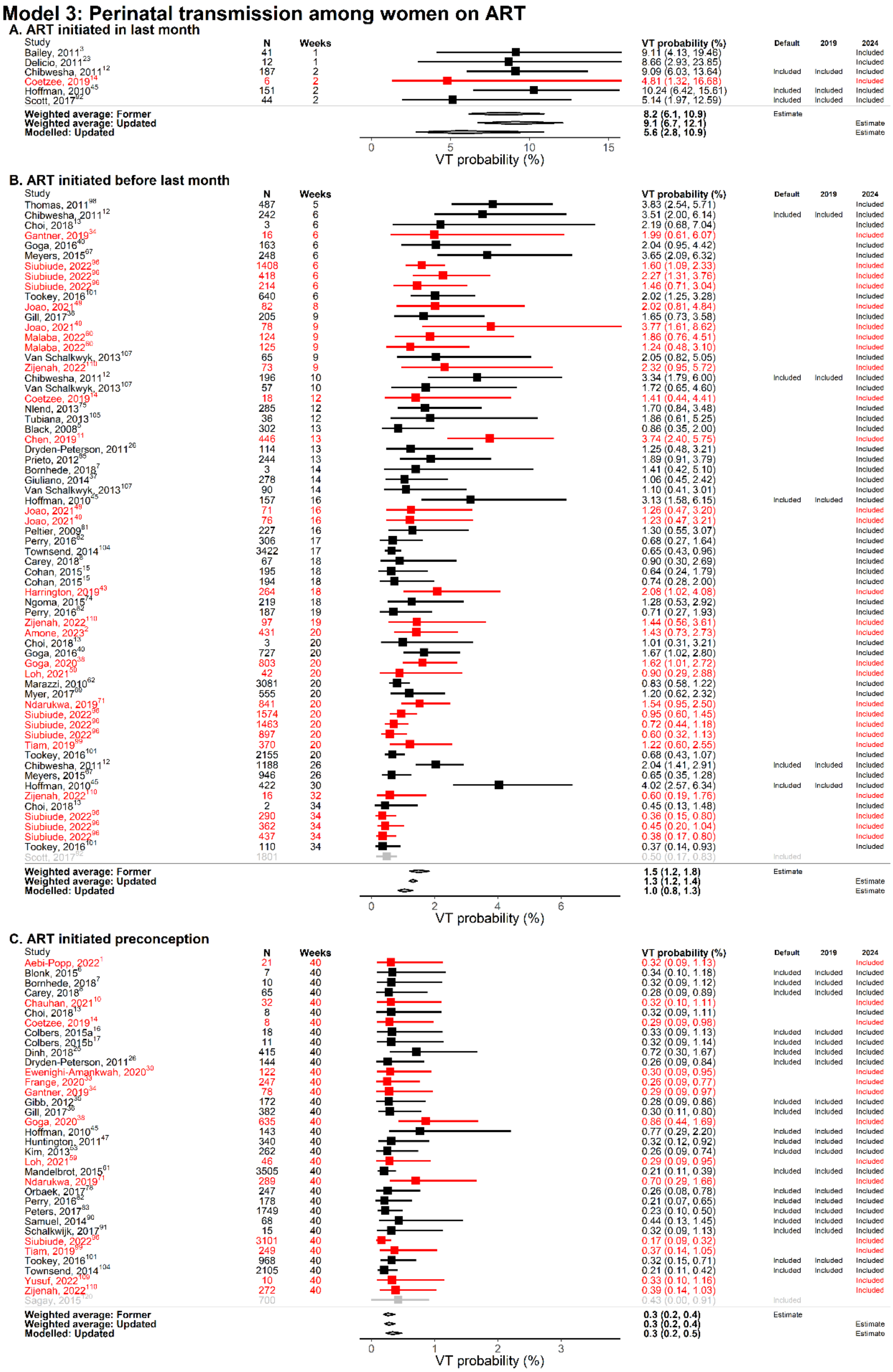

Figure 3.2.14 Pooled estimates of perinatal VT among women receiving ART. The following pooled estimates are presented: the weighted average of studies included in the former VT probabilities ('Weighted average: former VT'), the weighted average of studies included in this analysis ('Weighted average: Updated'), and the results of the model three ('Modelled: Updated'). Studies added in the 2024 review are shown in red and studies excluded from the meta-regression are shown in grey. Scott 2017 was excluded as we could not determine whether ART initiation occurred before or during pregnancy and Sagay 2015 was excluded as we could not determine paediatric test timing.

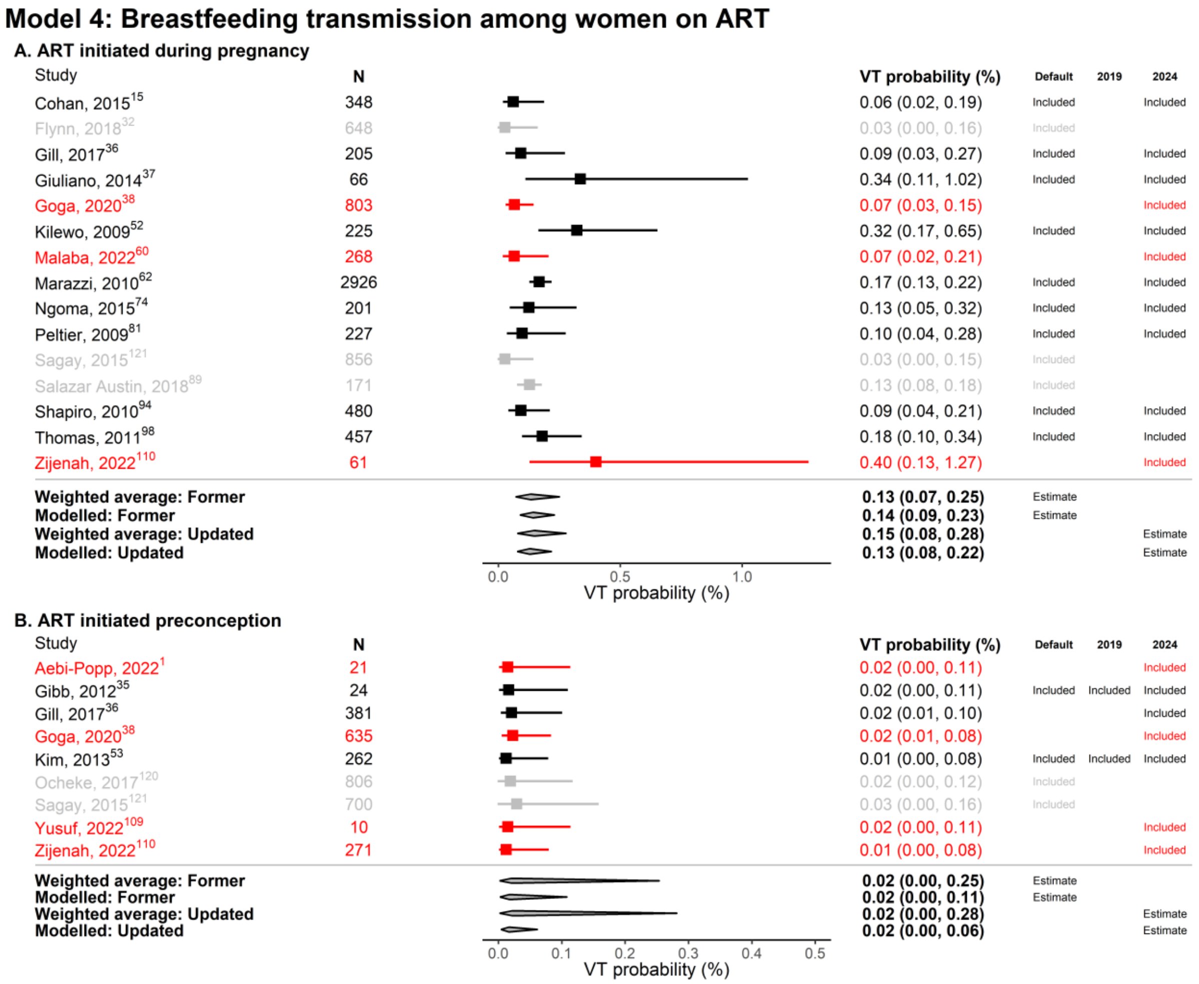

Figure 3.2.15 Pooled estimates of breastfeeding VT among women receiving ART. The following pooled estimates are presented: the weighted average of studies included in the former VT probabilities ('Weighted average: former VT'), estimates from model four fit to the pre-2024 systematic review studies ('Modelled: former VT'), the weighted average of studies included in this analysis ('Weighted average: Updated'), and the results of the model four ('Modelled: Updated').Studies added in the 2024 review are shown in red and studies excluded from the meta-regression are shown in grey. Flynn 2018, Ocheke 2017, and Salazar Austin 2018 were excluded as we could not determine whether ART initiation occurred before or during pregnancy and Sagay 2015 was excluded as we could not determine paediatric test timing.

4. Sensitivity analyses on meta-regression model assumptions

4.1. Model one: VT probability from women not receiving PVT

Model one included data from 16 studies that reported data about transmissions among women who did not receive PVT stratified by CD4 count. This including two new studies published since the 2018 review (Supplementary material 3.1). For the continuous CD4 covariate in model 1, we summarized the CD4 distribution among the study population by the approximate midpoint CD4. Here we assess (1) the sensitivity of model estimates to how the CD4 midpoint was defined from each study and (2) the sensitivity of estimates at different CD4 midpoints.

4.1.1. Sensitivity to CD4 midpoint calculation

Data included in model one reported CD4 among the study population in one of two formats: (1) women with CD4 in a given range (e.g. 200 – 350 mm^3) or (2) median CD4 of women who did not receive PVT care at baseline. If a study reported CD4 as a range, we took the median of the upper and lower bound (e.g. a range of 200-350 was extracted as 275). If a study reported CD4 using the second method (median CD4), the median was extracted.

Studies that stratified participates by CD4 range categories often reported vertical transmission probability data stratified by CD4 range, meaning that one study often reported multiple observations. Among studies that reported perinatal transmission among women not receiving PVT, four studies reported transmission by CD4 range. Three of the four studies reported transmission among three CD4 ranges. Nine studies reported perinatal transmission among women not on PVT that were not stratified by CD4 range, but did report a median CD4 among the study population. Among studies that reported breastfeeding transmission among women not receiving PVT, two studies reported transmission by CD4 range. One of the two studies reported transmission among multiple CD4 ranges. Four studies reported breastfeeding transmission among women not on PVT that were not stratified by CD4 range, but did report a median CD4 among the study population. Studies used in model one and method of CD4 reporting are listed in Table 4.1.1.1.

Table 4.1.1.1. Studies used in model one and method of CD4 reporting

Study	Location	Study years	Transmission type	CD4	VT	N	Method of CD4 reporting
**Hoffman, 2010**	South Africa	2004 - 2008	Perinatal	161	0.17	23	Median
**Coetzee, 2019**	South Africa	2010 - 2010	Perinatal	272	0.13	15	Median
**Goga, 2016**	South Africa	2011 - 2013	Perinatal	372	0.13	63	Median
**Olana, 2016**	Ethiopia	2006 - 2014	Perinatal	415	0.13	102	Median
**Petra Study Team, 2002**	Tanzania, South Africa, Uganda	2000 - 2000	Perinatal	435	0.13	303	Median
**Habib, 2021**	Iran	2015 - 2017	Perinatal	475	0.20	20	Median
**Dabis, 1999**	Ivory Coast,	1995 - 1998	Perinatal	535	0.29	145	Median

	Burkina Faso						
**Connor, 1994**	USA and France	1991 - 1993	Perinatal	538	0.22	183	Median
**Wiktor, 1999**	Ivory Coast	1996 - 1998	Perinatal	548	0.22	119	Median
**Marinda, 2011**	Zimbabwe	1997 - 2000	Perinatal	99.5	0.36	496	CD4 range
**Mayaux, 1995**	France	1986 - 1994	Perinatal	99.5	0.43	65	CD4 range
**Shaffer, 1999**	Thailand	1996 - 1997	Perinatal	99.5	0.38	24	CD4 range
**Marinda, 2011**	Zimbabwe	1997 - 2000	Perinatal	274.5	0.24	826	CD4 range
**Mayaux, 1995**	France	1986 - 1994	Perinatal	300	0.26	57	CD4 range
**Shaffer, 1999**	Thailand	1996 - 1997	Perinatal	349.5	0.18	104	CD4 range
**Iliff, 2005**	Zimbabwe	1997 - 2000	Perinatal	404	0.21	4367	CD4 range
**Marinda, 2011**	Zimbabwe	1997 - 2000	Perinatal	424.5	0.20	1963	CD4 range
**Mayaux, 1995**	France	1986 - 1994	Perinatal	600	0.17	192	CD4 range
**Shaffer, 1999**	Thailand	1996 - 1997	Perinatal	600	0.13	67	CD4 range
**Petra Study Team, 2002**	Tanzania, South Africa, Uganda	1996 - 2000	BF (monthly)	435	0.01	303	Median
**Chasela, 2010**	Malawi	2004 - 2010	BF (monthly)	442	0.01	668	Median
**Dabis, 1999**	Ivory Coast, Burkina Faso	1995 - 1998	BF (monthly)	535	0.01	113	Median
**Wiktor, 1999**	Ivory Coast	1996 - 1998	BF (monthly)	548	0.02	115	Median
**Kuhn, 2010**	Zambia		BF (monthly)	174.5	0.01	524	CD4 range
**Coovadia Fowler, 2012**	South Africa, Tanzania, Uganda, Zimbabwe	2008 - 2011	BF (monthly)	424.5	0.01	434	CD4 range
**Kuhn, 2010**	Zambia		BF (monthly)	424.5	0.00	469	CD4 range

As these two formats (CD4 range and median CD4) are not directly comparable, we conducted a subgroup analysis to assess the sensitivity of model one results to the difference in method of CD4 reporting. The subgroups we analysed were (1) studies that reported a CD4 range ("CD4 range" model) and (2) studies that reported a CD4 median ("median CD4" model). We re-fit model one to these different subgroups using Equation 4.1. Model one's fit to all data as presented in the main text is also copied below ("all data" model).

$$\text{logit}(VT_{BF,CD4,S,Obs}) = \beta_{0,BF=0} + \beta_{1,BF=1} + \beta_2 * CD4_{Midpoint} + \beta_{3,BF=1} * CD4_{Midpoint} + \mu_S + \mu_{Obs}$$

Equation 4.1

The following description of Equation 4.1 is copied from the main text: "*Model one included fixed effects for perinatal (β_0) and monthly breastfeeding VT (β_1), CD4 midpoint of the study population (β_2, per 100 mm^3, centred at 500 mm^3), and the interaction between CD4 midpoint and breastfeeding timing (β_3). CD4 midpoint was extracted as the median CD4 of WLHIV not receiving ARVs or the midpoint of relevant CD4 range if studies reported VT by CD4 categories… Random effects were included for study and observation (μ_0 and μ_1, respectively).*"

Table 4.1.1.2. Sensitivity analysis is of model 1 results to CD4 midpoint assumption for data used in model one

Covariate	Estimates of the "all data" model (logit) (n = 12,021)		Estimates of the "CD4 range" model (logit) (n = 9642)		Estimates of the "median CD4" model (logit) (n = 2379)	
**Intercept**	-1.61	(-1.81, -1.41)	-1.57	(-1.65, -1.48)	-1.47	(-1.63, -1.30)
**CD4 midpoint** (per 100 cells increase, centered on CD4 = 500 mm^3)	-0.23	(-0.28, -0.17)	-0.24	(-0.29, -0.18)	0.33	(0.10, 0.56)
**Perinatal transmission** (Reference)	0.00	(Reference)	0.00	(Reference)	0.00	(Reference)
**Breastfeeding transmission**	-3.22	(-3.84, -2.60)	-4.05	(-5.33, -2.77)	-2.88	(-3.58, -2.19)
**Interaction between CD4 midpoint and breastfeeding transmission**	0.17	(-0.21, 0.54)	-0.08	(-0.59, 0.43)	0.20	(-0.92, 1.32)

The covariates of the "all data" model one and "CD4 range" model one fit had overlapping confidence intervals (Table 4.1.1.2). The "all data" model one and "median CD4" model one fit were significantly different from each other. While the "all data" model one had a negative estimate for the effect of CD4 midpoint on VT probability (-0.2 (-0.3 - -0.2)), the "median CD4" model had a positive estimate for the effect of CD4 midpoint on VT probability (0.4 (0.1-0.7)).

The difference in effect of CD4 midpoint on VT probability among women not receiving PVT was driven by three studies that reported both high CD4 medians and transmission rates (Dabis 1999, Connor 1994, and Wiktor 1999, Table 4.1.1.1). Excluding these three studies resulted in a negative CD4 midpoint fixed effect. These studies collected data in the early stage of the HIV epidemic (1991 to 1998 across the three studies), reported high transmission rates and CD4 medians. This may may reflect high incidence among women of childbearing age. In acute HIV infection, both CD4 and viral load are high, and so women with high CD4 can have higher risk of vertical transmission than women no longer in the acute infection stage.[4] Dabis 1999 describes data from the placebo group of the DITRAME trial which had high average CD4 and viral load; among women who transmitted the mean CD4 was 358 and the mean viral load was approximately 55,000.[5] Similar data was not available Connor 1994 and Wiktor 1999.

The observations from these three studies (Dabis, Connor, and Wiktor) represent 31% of the available data in the "median CD4" model and are the only observations with CD4 midpoints

above 500. The results of the "median CD4" model are confounded by studies that reflect an earlier HIV epidemic, where transmission probability may be high among high CD4 values due to high incidence rates. When all data is used, the effect of these studies is diluted as there is more data about transmission among women with high CD4 values. The meta-regression framework then imposes larger study and observation level random effects on observations from Dabis 1999, Connor 1994, and Wiktor 1999. Although subgroup analysis shows that model one results are sensitive to the format of CD4 midpoint reporting, the difference in transmission may be confounded by other study characteristics (specifically study year and epidemic stage) rather than due to reporting a CD4 median itself.

4.1.2 Sensitivity to CD4 midpoint used to produce estimates for Spectrum

For women not receiving PVT, Spectrum-AIM stratifies transmission probabilities by CD4 range. These are CD4 <200, 200-350, and >350. To produce model based estimates of VT probabilities compatible with Spectrum-AIM's stratification, we used CD4 midpoints of 100, 275, and 500 ("Main text value" in Table 4.1.2). We assessed the sensitivity of our estimates to this choice by considering alternate uses of model one that could be used to approximate the Spectrum-AIM CD4 categories. Alternate approaches included: the mean of all transmission probabilities for CD4 midpoints within the Spectrum-AIM ranges ("Mean across range" in Table 4.1.2), the lowest CD4 of the Spectrum-AIM ranges ("Lowest CD4" in Table 4.1.2), and the highest CD4 of the Spectrum-AIM range ("Highest CD4" in Table 4.1.2). For the >350 category, the highest CD4 we considered was 650 mm^3.

Table 4.1.2 Sensitivity of model one VT estimates to CD4 midpoint chosen to produce Spectrum-AIM compatible estimates

	CD4 range	Main text value		Mean across range		Lowest CD4		Highest CD4	
**Perinatal**	<200	33.4	(27.8, 39.1)	33.1	(26.0, 41.8)	38.8	(32.0, 45.7)	28.6	(24.0, 32.7)
	200-350	25.1	(21.4, 28.9)	25.1	(20.0, 30.8)	28.6	(24.0, 32.6)	22.1	(18.8, 25.4)
	>350	16.6	(13.9, 19.7)	16.8	(11.4, 23.2)	22.1	(18.8, 25.2)	12.5	(9.9, 15.7)
**BF (monthly)**	<200	1.0	(0.3, 3.3)	1.1	(0.4 3.5)	1.2	(0.3, 4.8)	1.0	(0.5, 2.2)
	200-350	0.9	(0.5, 1.7)	0.9	(0.5, 1.8)	1.0	(0.5, 2.2)	0.9	(0.5, 1.5)
	>350	0.8	(0.4, 1.4)	0.8	(0.4, 1.5)	0.9	(0.5, 1.5)	0.7	(0.3, 1.9)

Perinatal transmission rates were within a -4.7% to 5.6% absolute difference range of the main text values estimated using the CD4 midpoints of 100, 275, and 500. Breastfeeding transmission rates were within a -0.1% to 0.1% absolute difference range of the main text values. The largest difference was among perinatal transmission with CD4 >350 (Table 4.1.2). The midpoint of 500 produced a transmission probability of 16.7% (13.8–20.0%). The highest CD4 in that range (CD4 = 650) had a transmission rate of 12.5% (9.9%–15.7%) while the lowest CD4 in that range (CD4 = 351) had a transmission rate of 22.1% (18.8–25.2%). If CD4 testing data shows that most women have high CD4 values (> 500), the VT probability used in Spectrum may be overestimating VT. However, given high PVT coverage, most women who are exposed to the VT probabilities for women not receiving PVT will have been those who were not retained on care. Treatment interruption is associated with rapid decline in CD4 count and increase in viral load after interruption, indicating that VT probability in this group is unlikely to be underestimated.[6]

4.2. Model three: perinatal transmission probability from women receiving ART by timing of initiation

Model three included data from 57 studies (Supplementary material 3.3). Here we assess the sensitivity of model three's fit to the calculation used to extract a time on ART midpoint from each study.

4.2.1 Sensitivity to time on ART data extraction

Publications reported time on ART before delivery in one of four ways: (1) median or mean number of weeks on ART before delivery, (2) median or mean gestational week of ART initiation, (3) a range of gestational weeks when women initiated ART (either reported as a trimester or through exclusion criteria), and (4) women who were on ART pre-conception.

To maximize the number of studies used, we used all reporting types and attributed a number of "weeks on ART midpoint" for studies where women used lifelong ART during pregnancy. The minimum value accepted was 0.5, indicating that the women initiated ART one week before delivery and the maximum value accepted was 40, indicating that the women initiated ART preconception. We preferentially extracted median (or mean) weeks on ART before delivery or median (mean) gestational week of ART initiation. As described in Supplementary Material 2, for countries that reported time on ART using the third method (a range of weeks), we contacted authors to request a median number of weeks on ART before delivery for the cohort. For the remaining studies where median time on ART was not available and the only information was a range of weeks when women initiated ART, we used the median of this range to determine the weeks on ART midpoint. The method used to derive the weeks on ART is shown for each study used in model three in Table 4.2.1.1.

Table 4.2.1.1. Data used to estimate effect of time on ART on VT estimates

Study	Location	Study years	Weeks on ART midpoint	VT events	N	Method to derive number of weeks on ART[A]
**Bailey, 2011**	Europe	2000 - 2009	1	5	41	3
**Delicio, 2011**	Brazil	2000 - 2009	1	2	12	3
**Chibwesha, 2011**	Zambia	2007 - 2010	2	17	187	3
**Coetzee, 2019[B]**	South Africa	2010 - 2010	2	0	6	2
**Hoffman, 2010**	South Africa	2004 - 2008	2	14	151	3
**Scott, 2017**	USA	2002 - 2009	2	2	44	3
**Tubiana, 2013**	France	2007 - 2010	3.2	1	36	1
**Thomas, 2011**	Kenya	2003 - 2009	5	20	487	3
**Choi, 2018**	Korea	2005 - 2017	6	0	3	3
**Gantner, 2019**	France	2008 - 2014	6	0	16	3
**Goga, 2016**	South Africa	2011 - 2013	6	2	163	3
**Meyers, 2015**	China	2010 - 2012	6	11	248	3
**Siubiude, 2017**	France	2000 - 2023	6	36	2152	3
**Tookey, 2016**	UK	2003 - 2013	6	13	640	3
**Malaba, 2018 [B]**	South Africa And Uganda	2022 - 2018	9	3	135	2
**Malaba, 2018 [B]**	South Africa And Uganda	2022 - 2018	9	0	133	2
**Van Schalkwyk, 2013[B]**	South Africa	2008 - 2010	9	2	65	2

**Zijenah, 2022 [B]**	Zimbabwe	2017 - 2018	9	2	73	2
**Gill, 2017**	Rwanda	2013 - 2014	9.6	3	205	1
**Van Schalkwyk, 2013[B]**	South Africa	2008 - 2010	10	1	57	2
**Joao, 2021**	Argentina, Brazil, South Africa, Tanzania, Thailand, USA	2013 - 2018	11.5	7	393	3
**Nlend, 2013**	Cameroon	2012 - 2008	12	5	285	1
**Black, 2008**	South Africa	2004 - 2007	13	1	302	2
**Chen, 2019**	China	2007 - 2015	13	19	446	3
**Prieto, 2012**	Spain	2000 - 2007	13	5	244	3
**Dryden-Peterson, 2011**	Botswana	2009 - 2010	13.1	1	114	2
**Bornhede, 2018**	Sweden	2014 - 2017	14	0	3	3
**Giuliano, 2014**	Malawi	2008 - 2009	14	2	278	2
**Van Schalkwyk, 2013[B]**	South Africa	2008 - 2010	14.6	0	90	2
**Townsend, 2014**	UK and Ireland	2007 - 2011	17.3	21	3422	2
**Perry, 2016**	UK	2007 - 2012	17.9	1	306	2
**Carey, 2018**	UK	2008 - 2014	18	0	67	2
**Harrington, 2019**	Malawi	2015 - 2016	18	7	264	2
**Ngoma, 2015**	Zambia	2008 - 2009	18	3	219	3
**Cohan, 2015**	Uganda	2009 - 2013	18.8	1	374	2
**Perry, 2016**	UK	2007 - 2012	19.6	1	187	2
**Zijenah, 2022[B]**	Zimbabwe	2017 - 2018	19	2	97	2
**Amone, 2016**	Uganda	2017 - 2023	20	7	431	3
**Choi, 2018**	Korea	2005 - 2017	20	0	3	3
**Goga, 2016**	South Africa	2011 - 2013	20	14	727	3
**Loh, 2021**	Singapore	2008 - 2015	20	0	42	3
**Myer, 2017**	South Africa	2013 - 2014	20	7	555	2
**Ndarukwa, 2019**	Zimbabwe	2014 - 2016	20	13	841	3
**Siubiude, 2017**	France	2000 - 2023	20	31	4147	3
**Tiam, 2019**	Lesotho	2014 - 2016	20	5	370	3
**Tookey, 2016**	UK	2003 - 2013	20	13	2155	3
**Marazzi, 2010**	Malawi and Mozambique	2005 - 2009	20.1	25	3081	2
**Chibwesha, 2011**	Zambia	2007 - 2010	22	42	1626	3
**Hoffman, 2010**	South Africa	2004 - 2008	22	28	579	3
**Van Schalkwyk, 2013[B]**	South Africa	2008 - 2010	24	1	127	3
**Meyers, 2015**	China	2010 - 2012	26	5	946	3
**Coetzee, 2019[B]**	South Africa	2010 - 2010	28	0	18	2
**Zijenah, 2022[B]**	Zimbabwe	2017 - 2018	32	0	16	2
**Choi, 2018**	Korea	2005 - 2017	34	0	2	3
**Siubiude, 2017**	France	2000 - 2023	34	6	1149	3
**Tookey, 2016**	UK	2003 - 2013	34	0	110	3
**Aebi-Popp, 2022**	Switzerland	2019 - 2021	40	0	17	4
**Blonk, 2015**	Europe	2010 - 2014	40	0	7	4
**Bornhede, 2018**	Sweden	2014 - 2017	40	0	10	4
**Carey, 2018**	UK	2008 - 2014	40	0	65	4
**Chauhan, 2021**	India	2016 - 2018	40	0	32	4
**Choi, 2018**	Korea	2005 - 2017	40	0	8	4
**Coetzee, 2019[B]**	South Africa	2010 - 2010	40	0	8	4
**Colbers, 2015a**	Europe		40	0	18	4
**Colbers, 2015**	Europe	2009 - 2014	40	0	11	4
**Dinh, 2018**	Zimbabwe	2013 - 2013	40	5	415	4

**Dryden-Peterson, 2011**	Botswana	2009 - 2010	40	0	144	4
**Ewenighi-Amankwah, 2020**	Nigeria		40	0	122	4
**Frange, 2020**	France	2010 - 2018	40	0	247	4
**Gantner, 2019**	France	2008 - 2014	40	0	78	4
**Gibb, 2012**	Uganda, Zimbabwe	2003 - 2009	40	0	172	4
**Gill, 2017**	Rwanda	2014 - 2016	40	1	382	4
**Goga, 2020**	South Africa	2012 - 2014	40	7	635	4
**Hoffman, 2010**	South Africa	2004 - 2008	40	1	143	4
**Huntington, 2011**	UK	1996 - 2009	40	1	340	4
**Kim, 2013**	Malawi	2009 - 2011	40	0	262	4
**Loh, 2021**	Singapore	2008 - 2015	40	0	46	4
**Mandelbrot, 2015**	France	2000 - 2011	40	6	3505	4
**Ndarukwa, 2019**	Zimbabwe	2014 - 2016	40	4	289	4
**Orbaek, 2017**	Denmark	2002 - 2014	40	0	247	4
**Perry, 2016**	UK	2007 - 2012	40	0	178	4
**Peters, 2017**	UK	2012 - 2014	40	3	1749	4
**Sagay, 2015**	Nigeria	2010 - 2012	40	3	700	4
**Samuel, 2014**	UK	2004 - 2010	40	1	68	4
**Schalkwijk, 2017**	Europe	-	40	0	15	4
**Siubiude, 2017**	France	2000 - 2023	40	9	6606	4
**Tiam, 2019**	Lesotho	2014 - 2016	40	1	249	4
**Tookey, 2016**	UK	2003 - 2013	40	4	968	4
**Townsend, 2014**	UK and Ireland	2007 - 2011	40	4	2105	4
**Yusuf, 2022**	USA	-	40	0	1	4
**Zijenah, 2022[B]**	Zimbabwe	2017 - 2018	40	1	272	4

[A] (1) Reported median or mean weeks on ART before delivery
(2) Reported median or mean gestational week of ART initiation
(3) Reported a range of gestational weeks during which women initiated ART
(4) Reported women who were on ART pre-conception

[B] Median gestational weeks at ART initiation was not reported in text, however corresponding author provided this information upon request.

As these two formats are not directly comparable, we conducted a subgroup analysis to assess the sensitivity of model three results to the difference in method for extracting the "weeks on ART midpoint". The subgroups we analysed were (1) studies that reported a median number of weeks on ART before delivery, studies that reported ART was initiated in the last four weeks of pregnancy, and transmission among women on ART preconception (methods one, two, and four above, "Median weeks reported" below) and (2) studies that reported a range of gestational weeks when women initiated ART or transmission among women on ART preconception (methods three and four above, "Range of weeks reported" below). We re-fit model three to these different subgroups using Equation 4.2. Model three's fit to all data as presented in the main text is also copied below ("All data" model).

$$\text{logit}(VT_{BF=0,Weeks,late,S,Obs}) = \beta_0 + \beta_1 * T_{weeks} + \beta_2 * late\ initation + \mu_S + \mu_{Obs}$$

Equation 4.2

The following description of Equation 4.2 is copied from the main text: "*Model three included a fixed effect (β_1) for weeks on ART before delivery (T_{Weeks}, centred on ART initiated 20 weeks before delivery; specified as 40 weeks for women already on ART at conception), and a fixed effect for ART initiation less than four weeks before delivery (β_2, 'late initiation'). The 'late initiation' effect accounted for increased risk of unsuppressed VL at delivery when ART*

is initiated late in pregnancy. T_{Weeks} was extracted as the median weeks on ART before delivery, where available. For studies that reported ART initiation as a range of gestational weeks, we extracted the range midpoint. We contacted corresponding authors of studies that reported a range of gestational weeks when ART was initiated to request the median gestational week ART was initiated... Random effects were included for study and observation (μ_0 and μ_1, respectively)."

Table 4.2.1.2. Sensitivity analysis is of model three results to time on ART midpoint assumption

Covariate	Estimates of the "All data" model (logit) (n = 45,220)		Estimates of the "Median weeks reported" model (logit) (n = 34,831)		Estimates of the "Range of weeks reported" model (logit) (n = 26,311)	
**Intercept**	-4.55	(-4.79, -4.32)	-4.83	(-5.14, -4.54)	-4.38	(-4.65, -4.11)
**Weeks on ART before delivery (centered on 20 weeks)**	-0.06	(-0.07, -0.04)	-0.05	(-0.07, -0.03)	-0.06	(-0.08, -0.05)
**Late ART initiation (<4 weeks before delivery)**	0.68	(-0.05, 1.45)	1.51	(0.77, 2.30)	0.57	(-0.19, 1.38)

The "all data" model three and "range of weeks reported" model three fit were not significantly different from each other (Table 4.2.1.2). The "all data" model three and "median weeks reported" model three fit were significantly different from each other. The late ART initiation covariate was significantly positive in the "median weeks reported" model, whereas in the "all data" model it was positive, but not significant.

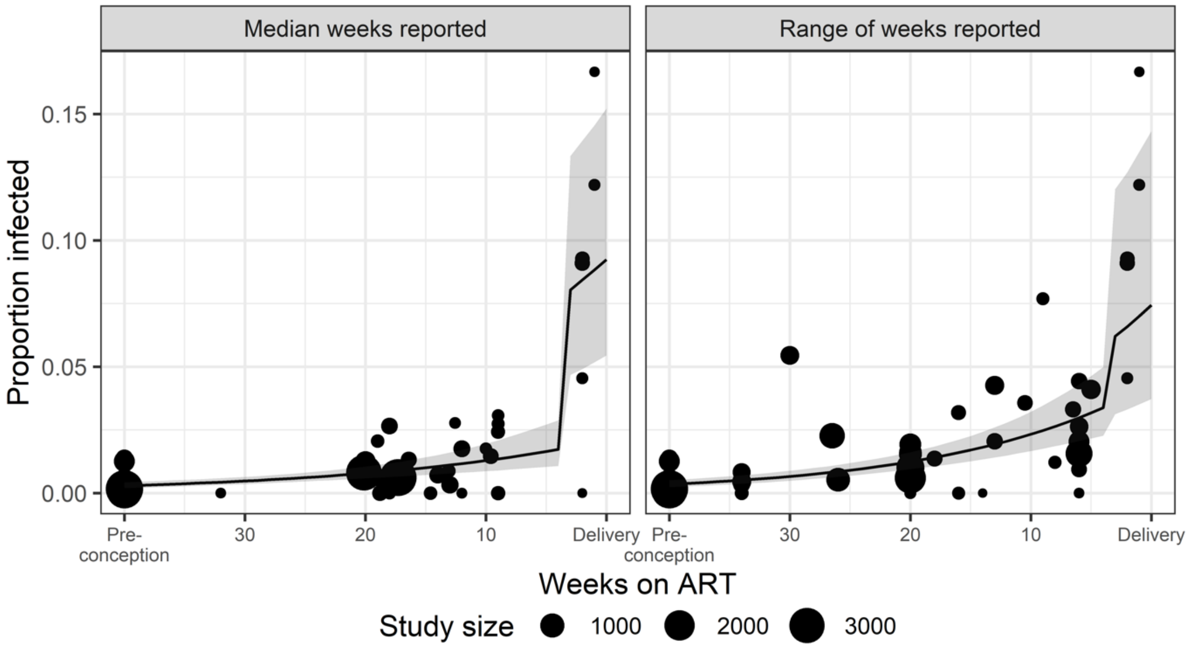

Figure 4.2.1. Effect of definition of weeks on ART midpoint on model three estimates of perinatal VT probability

The "all data" and "range of weeks reported" models included more studies where women initiated ART in the final ten weeks of pregnancy. These studies had VT probabilities ranging from 0% to approximately 7.5%, making it so that the studies with late ART initiation weren't significantly higher than what was captured in the "weeks on ART" covariate.

4.2.2 Sensitivity to assumption of 40 gestational weeks at delivery

For studies where we inferred the time on ART from the reported timing of ART initiation, we assumed that the average gestational age at delivery was 40 weeks ("full-term"). As not all women will deliver at full-term, we assessed the sensitivity of our results to this assumption. Margulis et al 2023[7] published estimates of gestational age at birth from U.S. birth data from the Center for Disease Control. Margulis et al reported a median gestational age at birth of 39 weeks. Similar data at the required granularity wasn't available for other regions, however, estimates of gestational age at birth from a cohort of South African women living with HIV were slightly lower, but similar (mean: 37.6 (range of 26-42)).[8]

For each study where we inferred the number of weeks on ART given delivery at full-term, we re-estimated the number of weeks on ART from the probability distribution of gestational age at delivery published by Margulis et al. Using the new dataset, we re-fit model 3 (Table 4.2.2.1). Model 3 fit to data that assumed full-term gestation and those that used the probability distributions published by Margulis et al produced similar results, with overlapping confidence intervals.

Table 4.2.2.1. Sensitivity analysis is of model three results to gestational age at delivery assumption

Covariate	Estimates of the full-term model (logit)		Estimates of the weighted gestational age model (logit)	
**Intercept**	-4.55	(-4.79, -4.32)	-4.63	(-4.87, -4.40)
**Weeks on ART before delivery (centered on 20 weeks)**	-0.06	(-0.07, -0.04)	-0.05	(-0.07, -0.04)
**Late ART initiation (<4 weeks before delivery)**	0.68	(-0.05, 1.45)	0.75	(0.11, 1.42)

The estimates of VT probability for model three fit to the weighted gestational age model were lower for women who initiate ART <4 weeks before delivery (Table 4.2.2.2). This was despite a higher odds ratio for the coefficient for late ART initiation (<4 weeks before delivery) in the weighted gestational age model was significantly greater than one (OR 2.12 (1.12-4.14)). Given that only 1% of WLHIV globally fall into this transmission category, Spectrum-AIM's estimates of vertical transmission aren't sensitive to these results. More work should be done to characterize gestational age at delivery among WLHIV in diverse global settings to improve these estimates.

Table 4.2.2.2. Sensitivity of model three estimates of VT probability to gestational age at delivery assumption

VT probability	Estimates of the full-term model (logit)		Estimates of the weighted gestational age model (logit)	
Option B+, on ART <4 weeks	5.6	(2.8 - 10.9)	4.8	(2.9 – 9.4)
Option B+, on ART 5-39 weeks	1.0	(0.8 - 1.3)	1.0	(0.8 - 1.2)
Option B+, on ART pre-conception	0.33	(0.23 - 0.48)	0.36	(0.19 - 0.55)

4.3 Geographic region as a confounder of ART class's effect on VT probability

Because 9/12 studies that reported perinatal transmission among women receiving an INSTI-based regimen occurred in high-income countries, we assessed geographic region as a confounder of ART class's effect on VT probability. To do so, we fit a modified version of model three, with fixed effects on ART class and geographic region. Geographic region was coded as: Sub-Saharan Africa (SSA, reference), non SSA, or multiple regions.

Table 4.3. Geographic region as a potential confounder for ART class's effect on VT probability

	No geographic region fixed effect		Geographic region fixed effect	
**Covariate**	Estimate (logit)	95% confidence interval	Estimate (logit)	95% confidence interval
**Intercept**	-4.45	(-4.71, -4.19)	-4.31	(-4.57, -4.05)
**Weeks on ART before delivery (centered on 20 weeks)**	-0.06	(-0.07, -0.04)	-0.06	(-0.07, -0.04)
**Late ART initiation (<4 weeks before delivery)**	0.72	(-0.05, 1.49)	1.05	(0.31, 1.79)
**ART class**				
**NNRTI (reference)**	0.00	(Reference)	0.00	(Reference)
**INSTI**	-1.01	(-1.95, -0.07)	-0.74	(-1.75, 0.26)
**PI**	-0.12	(-0.56, 0.32)	0.07	(-0.37, 0.51)
**Miscellaneous regimens**	-0.04	(-0.67, 0.59)	0.22	(-0.29, 0.72)
**Geographic region**	Not included			
**SSA**			0.00	(Reference)
**Non-SSA**			-0.58	(-0.95, -0.21)
**Multiple regions**			-0.18	(-1.07, 0.72)

In the model that included geographic region, the non-SSA region had the lowest VT probability when a NNRTI-based regimen was initiated 20 weeks before delivery, although the regions didn't differ significantly (Table 4.3). While in the model without geographic region specified INSTI-based regimens had significantly lower VT when ART was started 20 weeks before delivery, including geographic region in the mode made this effect not significant. This suggests that the effects of ART regimen class on perinatal VT are confounded by the study geographic region.

5. Implications of estimated VT probabilities for Spectrum-AIM's estimates of paediatric HIV infections

We used our estimates of VT probability in Spectrum-AIM model to assess the change in the number of perinatal and breastfeeding infections compared to those calculated using the former VT probabilities. For women not receiving any treatment, we used model one to estimated VT probability at the following CD4 midpoints: 100, 275, and 500. These were used to align with the Spectrum-AIM CD4 categories of <200, 200-350, and >350. For women receiving lifelong ART, used model three to estimate perinatal VT for weeks on ART of 2, 20, and 40 weeks to represent the Spectrum-AIM's perinatal transmission categories of on ART of <4 weeks, for 4-39 weeks before delivery, and preconception. For breastfeeding transmission probabilities, we used model four to estimate a monthly breastfeeding VT probability for women who initiated ART preconception and women who initiated ART during pregnancy. Probabilities of VT for women who seroconverted during pregnancy or breastfeeding and women who received short-course PVT were estimated using model two. The values used in this analysis are listed in Table 1 and Table 2 of the main text.

Using the 2024 published publicly available Spectrum-AIM files for Malawi, Rwanda, Democratic Republic of the Congo, and Burkina Faso, we calculated the percent change in the number of perinatal infections (Equation 5.1), breastfeeding infections (Equation 5.2), and total paediatric infections (Equation 5.3) in the years 2000, 2010, 2015, and 2023. These countries were chosen as they represent a country in Southern, Eastern, Central, and Western Africa respectively, and these years represent a variety of PVT strategies and coverages. These results do not necessarily reflect the potential results of applying these changes to other or all countries.

$$Percent\ change_{Perinatal} = \frac{(Infections_{Perinatal,MR} - Infections_{Perinatal,Former})}{Infections_{Perinatal,Former}} * 100$$

Equation 5.1

In Equation 5.1, $Infections_{Perinatal,MR}$ represented the number of perinatal infections that resulted from using the VT probabilities estimated in the meta-regression analysis, $Infections_{Perinatal,Former}$ represented the number of perinatal infections that resulted from using the former Spectrum-AIM VT probabilities, and $Percent\ change_{Perinatal}$ represented the percent change in perinatal infections.

$$Percent\ change_{BF} = \frac{(Infections_{BF,MR} - Infections_{BF,Default})}{Infections_{BF,Former}} * 100$$

Equation 5.2

In Equation 5.2, $Infections_{BF,MR}$ represented the number of breastfeeding infections that resulted from using the VT probabilities estimated in the meta-regression analysis, $Infections_{BF,Former}$ represented the number of breastfeeding infections that resulted from using the former Spectrum-AIM VT probabilities, and $Percent\ change_{BF}$ represented the percent change in breastfeeding infections.

$$Percent\ change_{Total} = \frac{(Infections_{Total,MR} - Infections_{Total,Former})}{Infections_{Total,Former}} * 100$$

Equation 5.3

In Equation 5.3, $Infections_{Total,MR}$ represented the number of vertical infections that resulted from using the VT probabilities estimated in the meta-regression analysis, $Infections_{Total,Former}$ represented the number of vertical infections that resulted from using the former Spectrum-AIM VT probabilities, and $Percent\ change_{Total}$ represented the percent change in vertical infections.

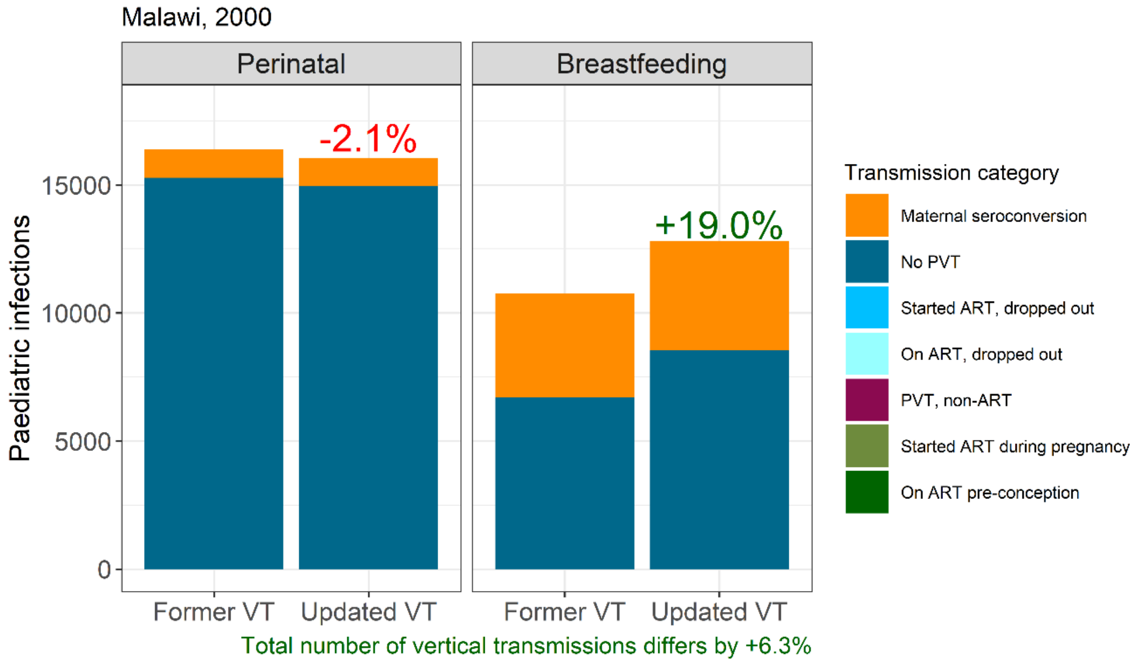

Figure 5.1. Change in vertical infections due to estimated vertical transmission probabilities by infection timing, Malawi 2000

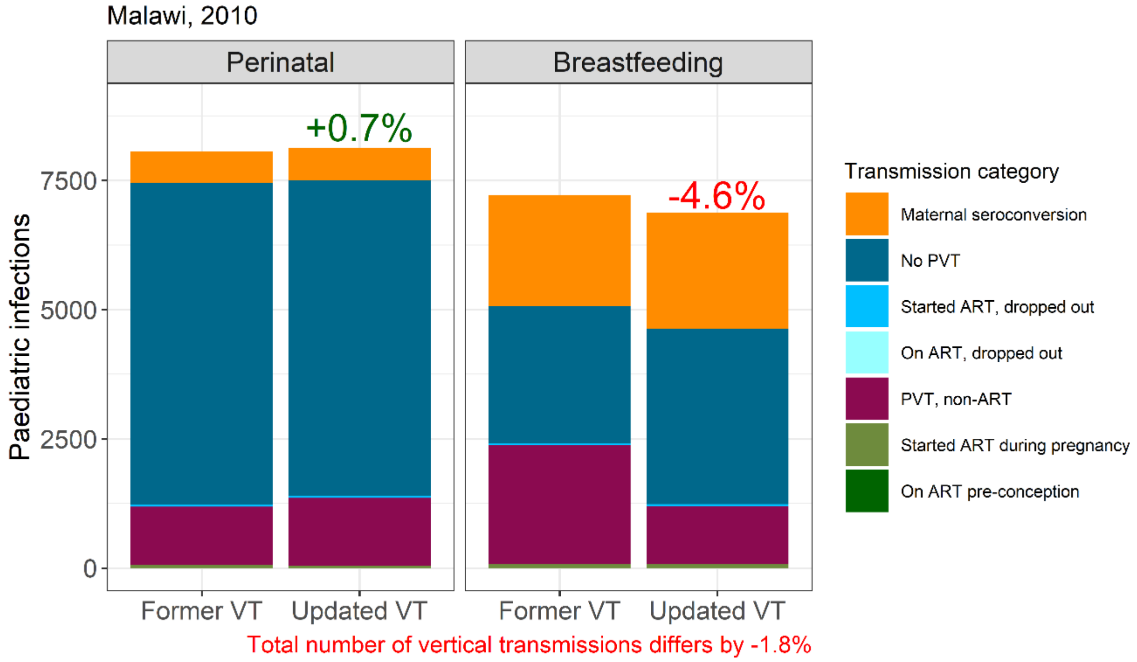

Figure 5.2. Change in vertical infections due to estimated vertical transmission probabilities by infection timing, Malawi 2010

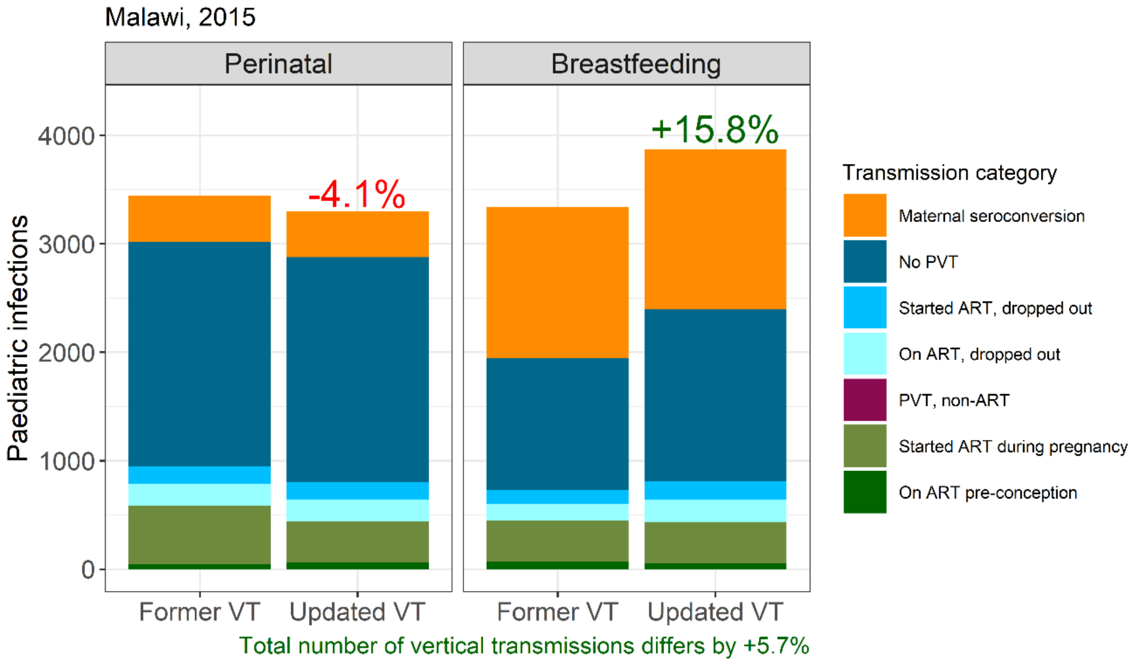

Figure 5.3. Change in vertical infections due to estimated vertical transmission probabilities by infection timing, Malawi 2015

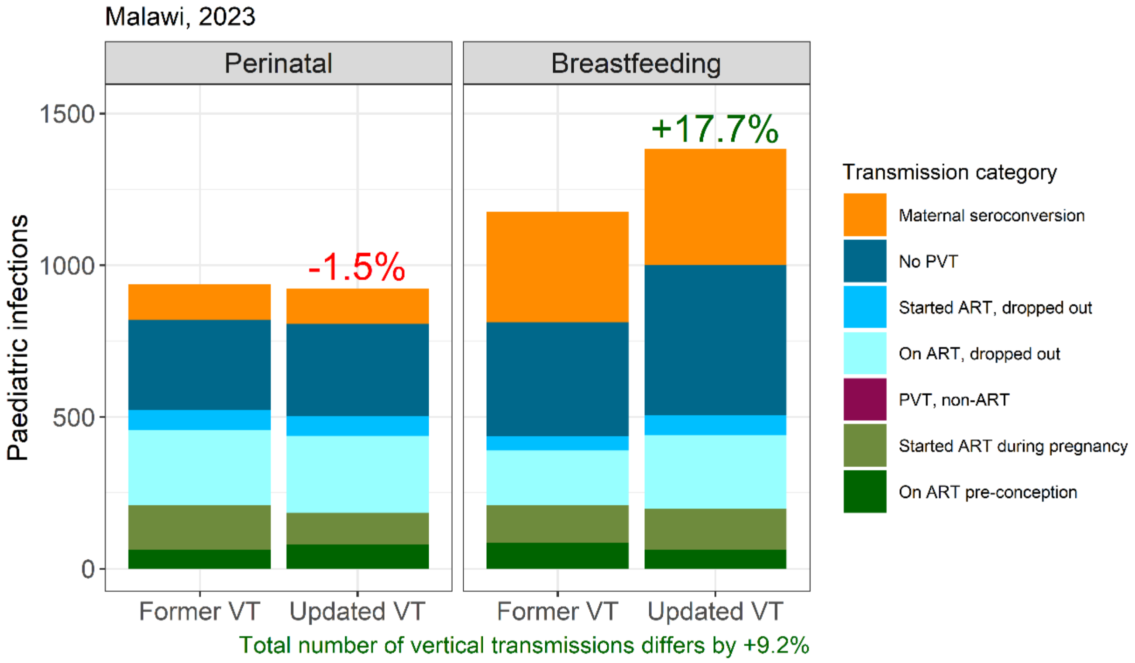

Figure 5.4. Change in vertical infections due to estimated vertical transmission probabilities by infection timing, Malawi 2023

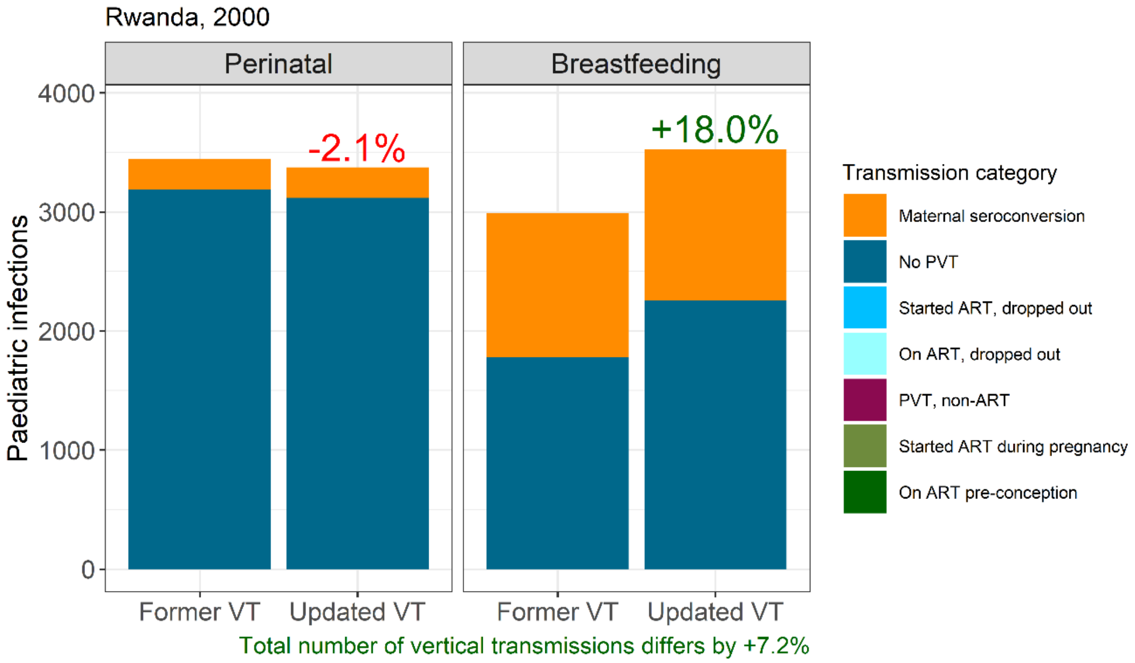

Figure 5.5. Change in vertical infections due to estimated vertical transmission probabilities by infection timing, Rwanda 2000

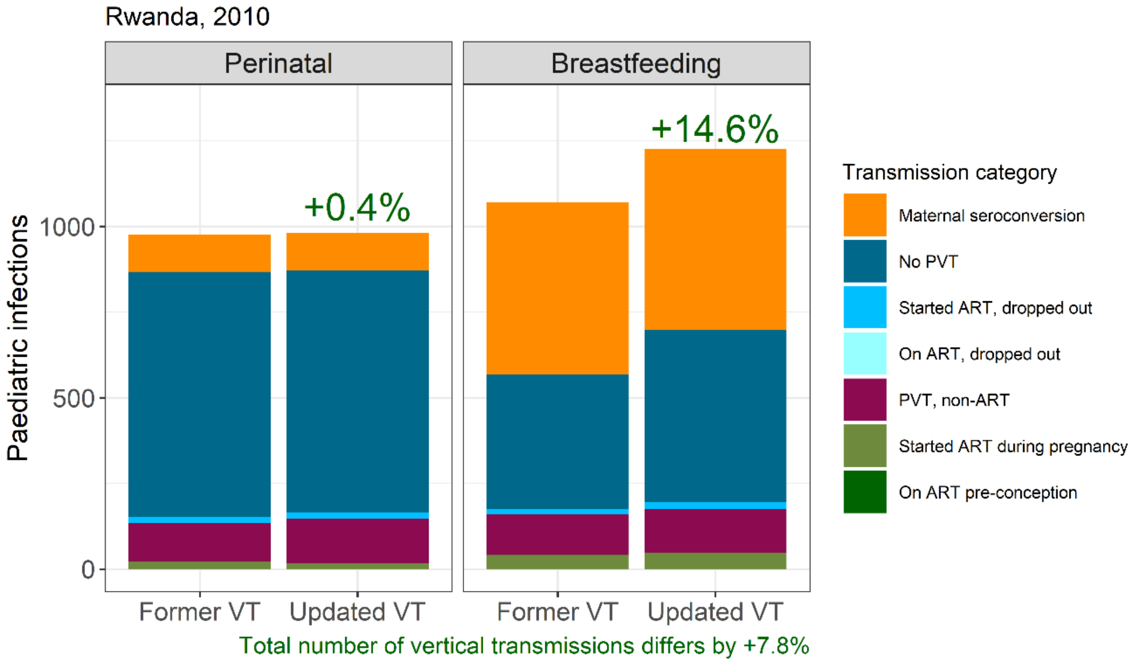

Figure 5.5. Change in vertical infections due to estimated vertical transmission probabilities by infection timing, Rwanda 2010

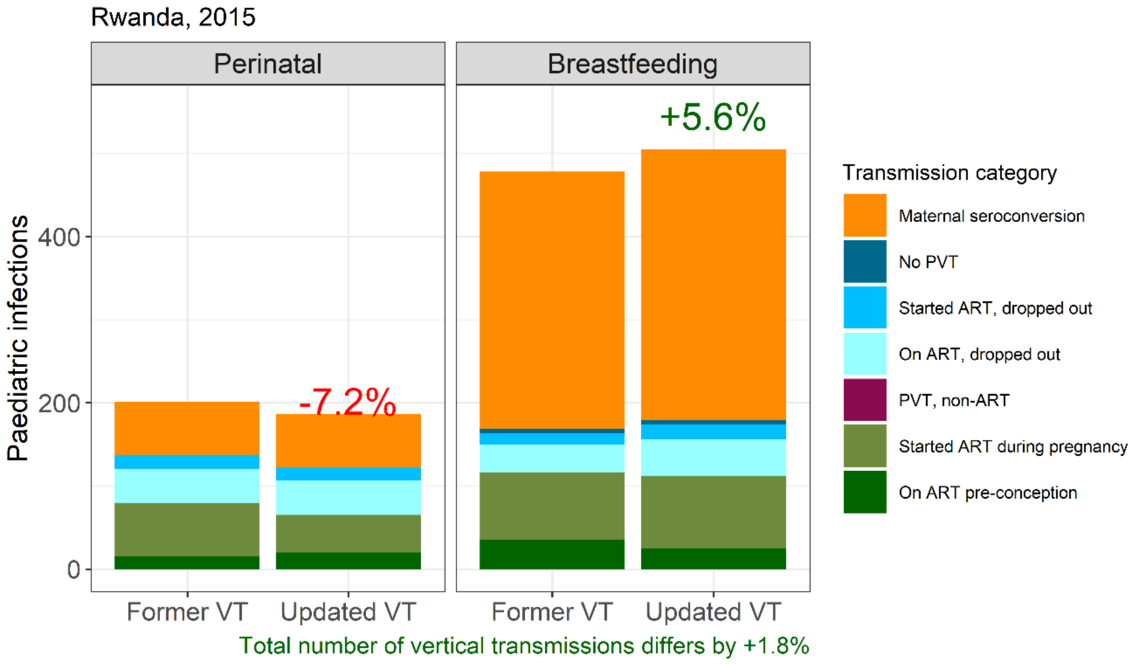

Figure 5.7. Change in vertical infections due to estimated vertical transmission probabilities by infection timing, Rwanda 2015

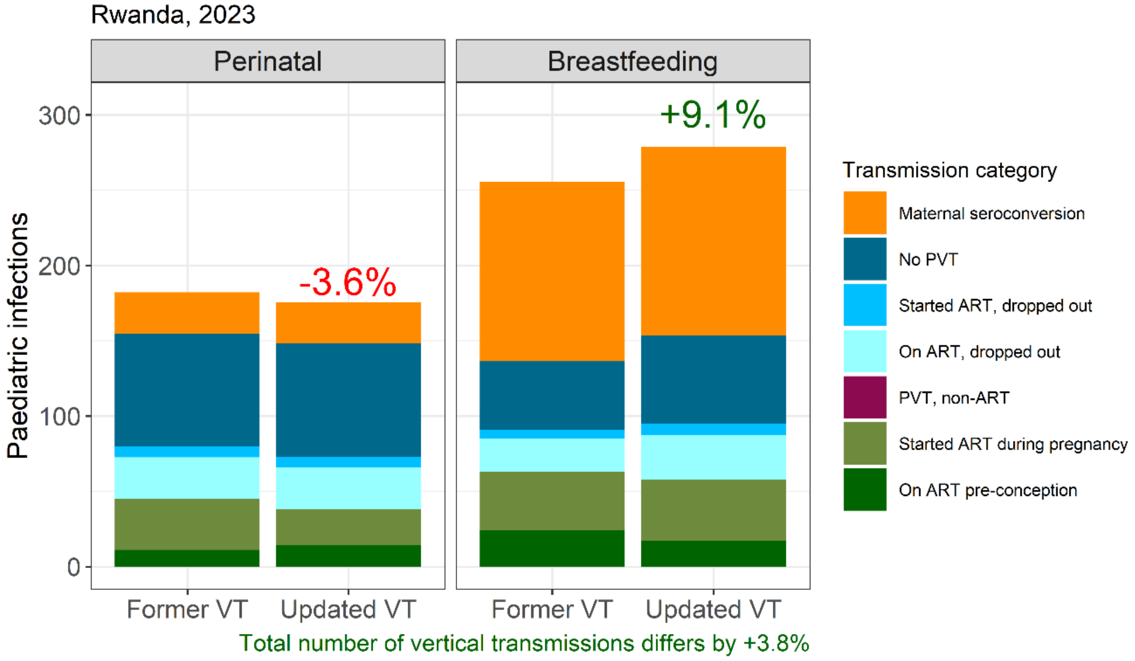

Figure 5.8. Change in vertical infections due to estimated vertical transmission probabilities by infection timing, Rwanda 2023

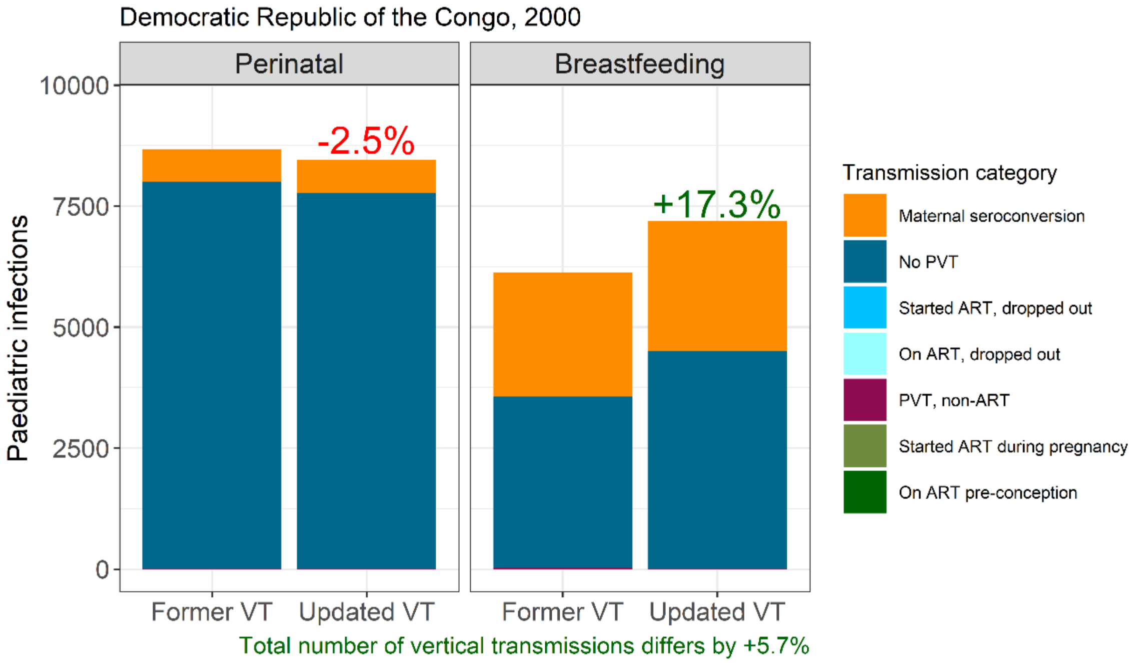

Figure 5.9. Change in vertical infections due to estimated vertical transmission probabilities by infection timing, Democratic Republic of the Congo 2000

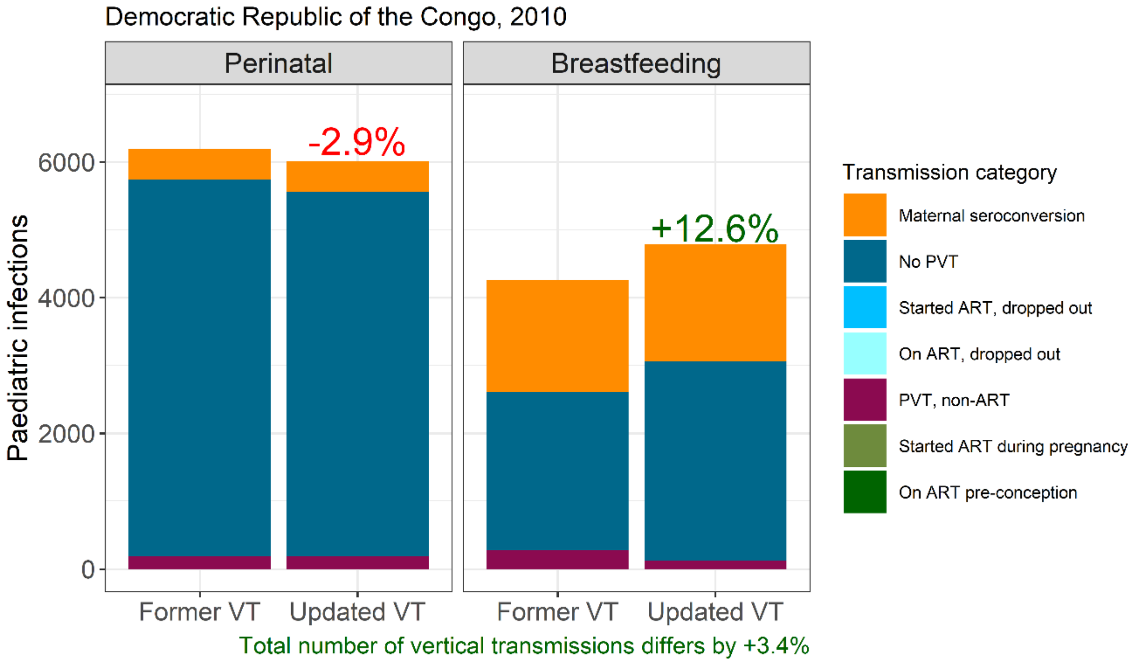

Figure 5.10. Change in vertical infections due to estimated vertical transmission probabilities by infection timing, Democratic Republic of the Congo 2010

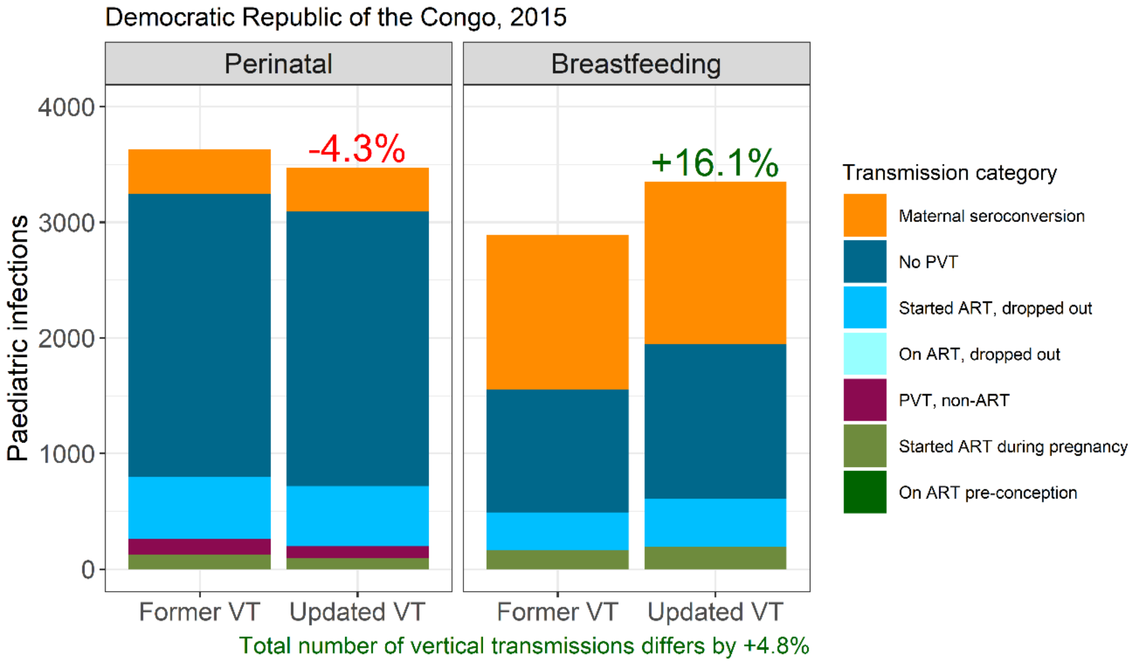

Figure 5.11. Change in vertical infections due to estimated vertical transmission probabilities by infection timing, Democratic Republic of the Congo 2015

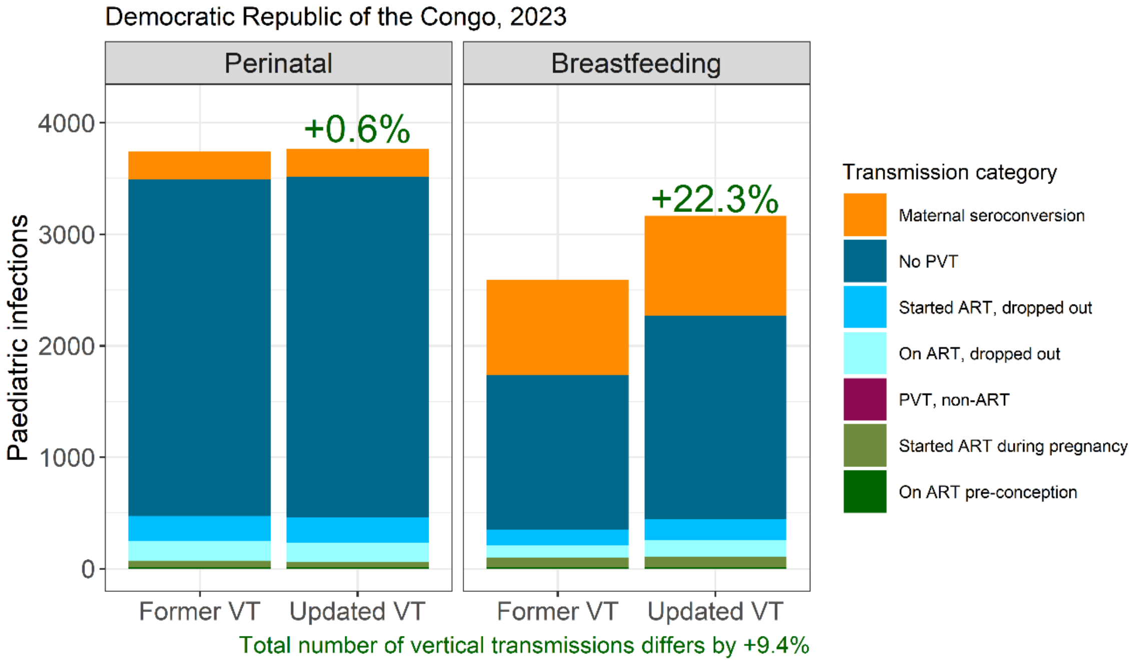

Figure 5.12. Change in vertical infections due to estimated vertical transmission probabilities by infection timing, Democratic Republic of the Congo 2023

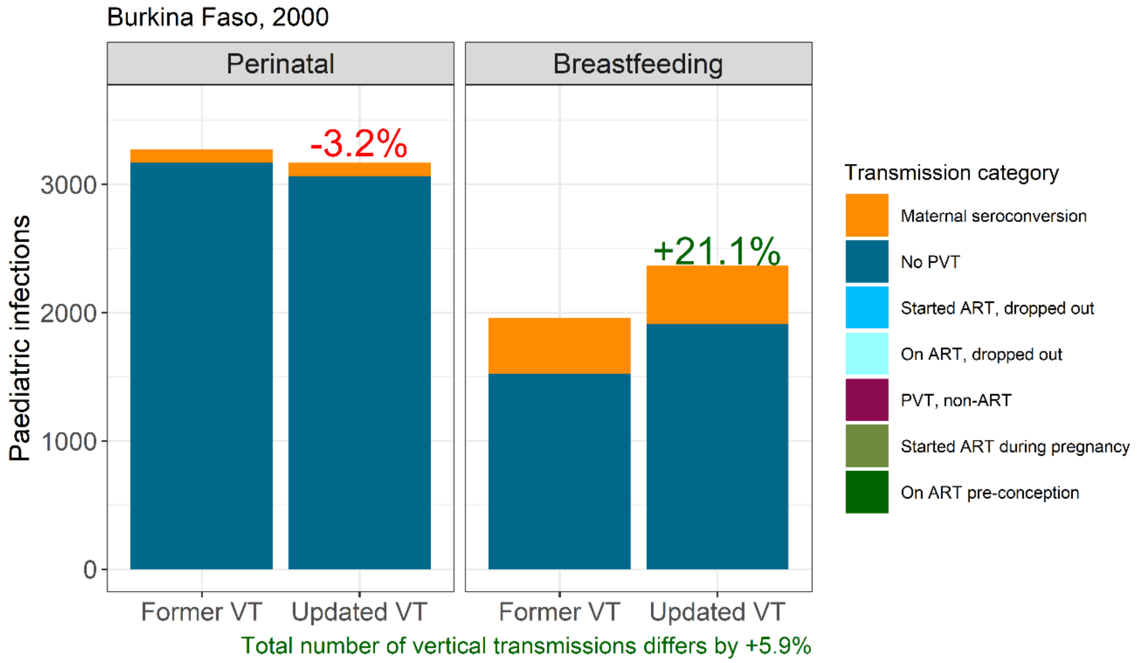

Figure 5.13. Change in vertical infections due to estimated vertical transmission probabilities by infection timing, Burkina Faso 2000

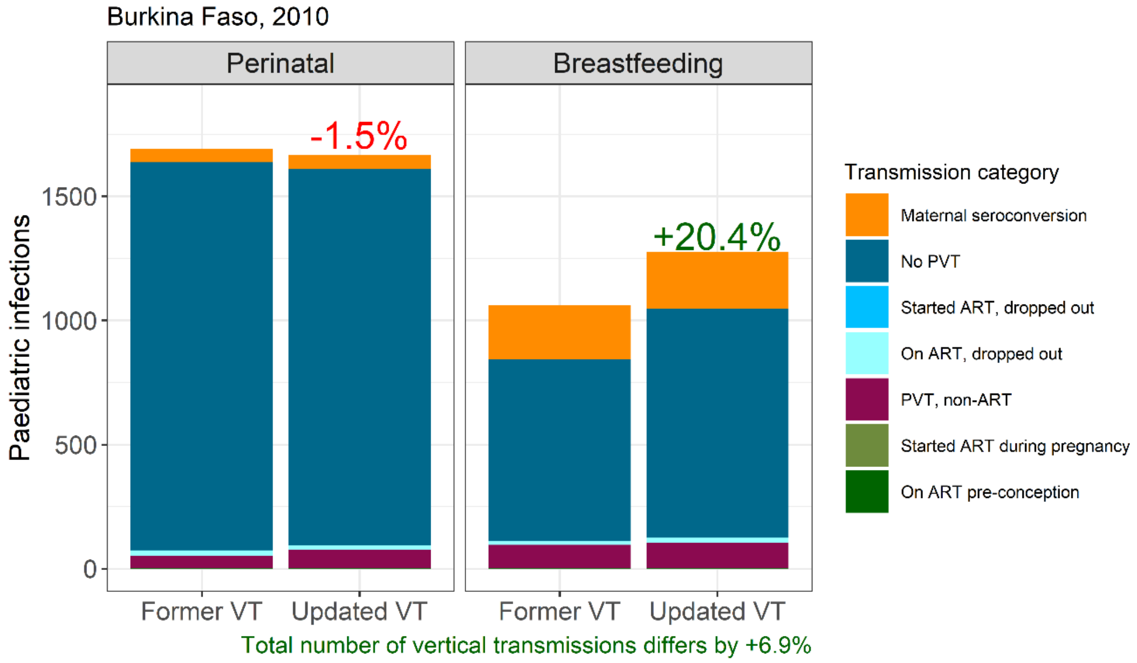

Figure 5.14. Change in vertical infections due to estimated vertical transmission probabilities by infection timing, Burkina Faso 2010

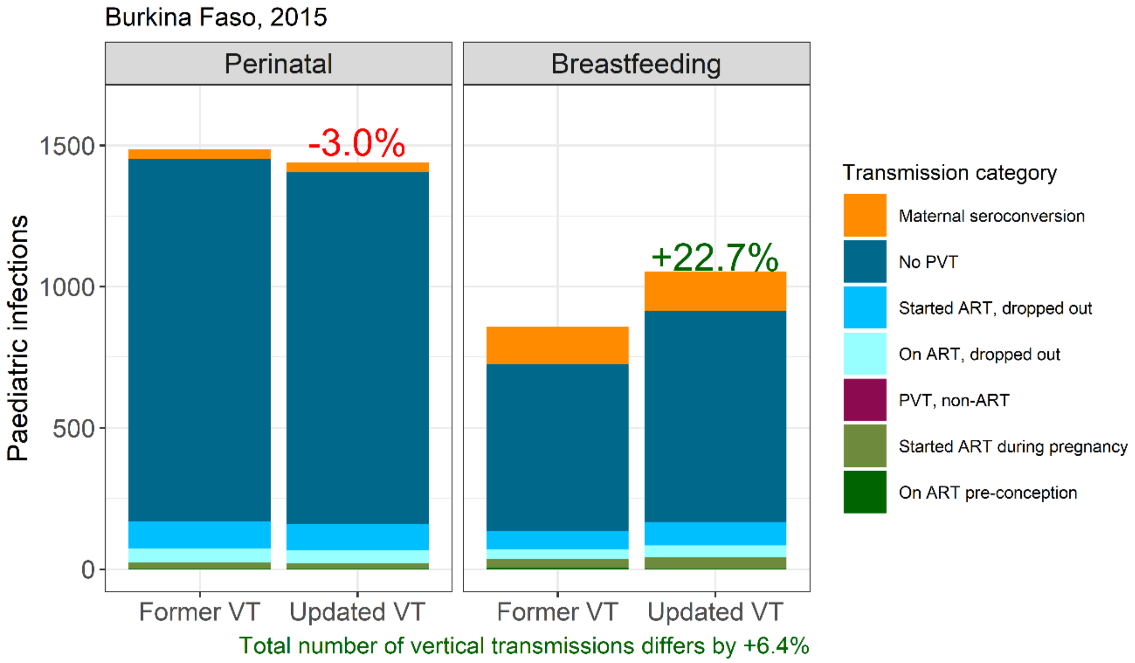

Figure 5.15. Change in vertical infections due to estimated vertical transmission probabilities by infection timing, Burkina Faso 2015

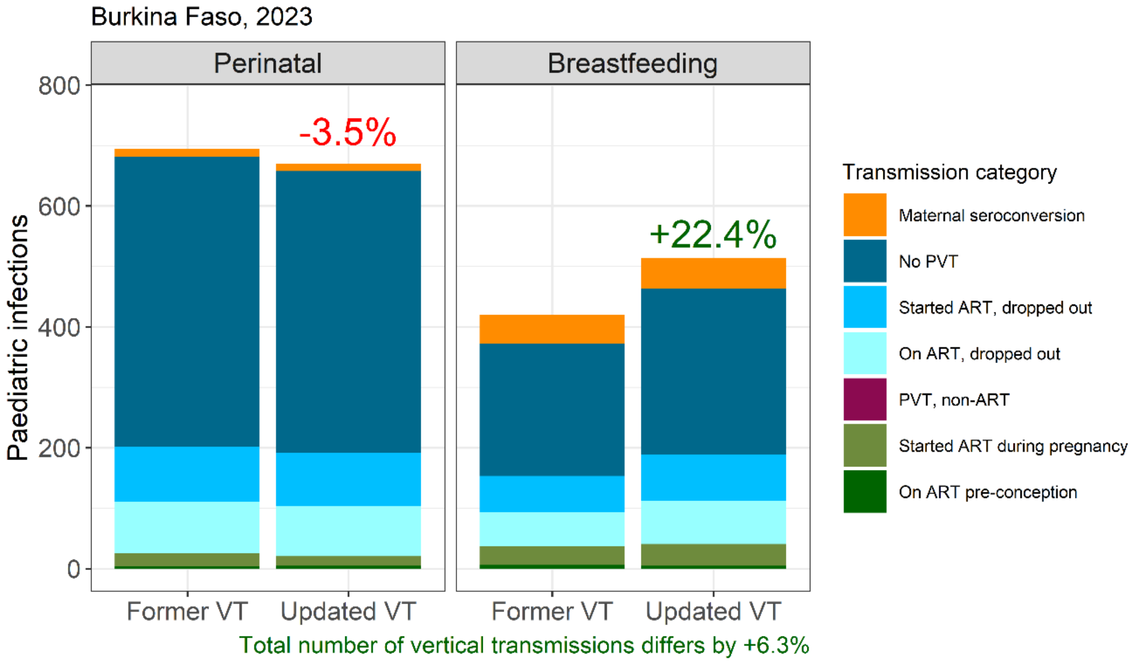

Figure 5.15. Change in vertical infections due to estimated vertical transmission probabilities by infection timing, Burkina Faso 2023

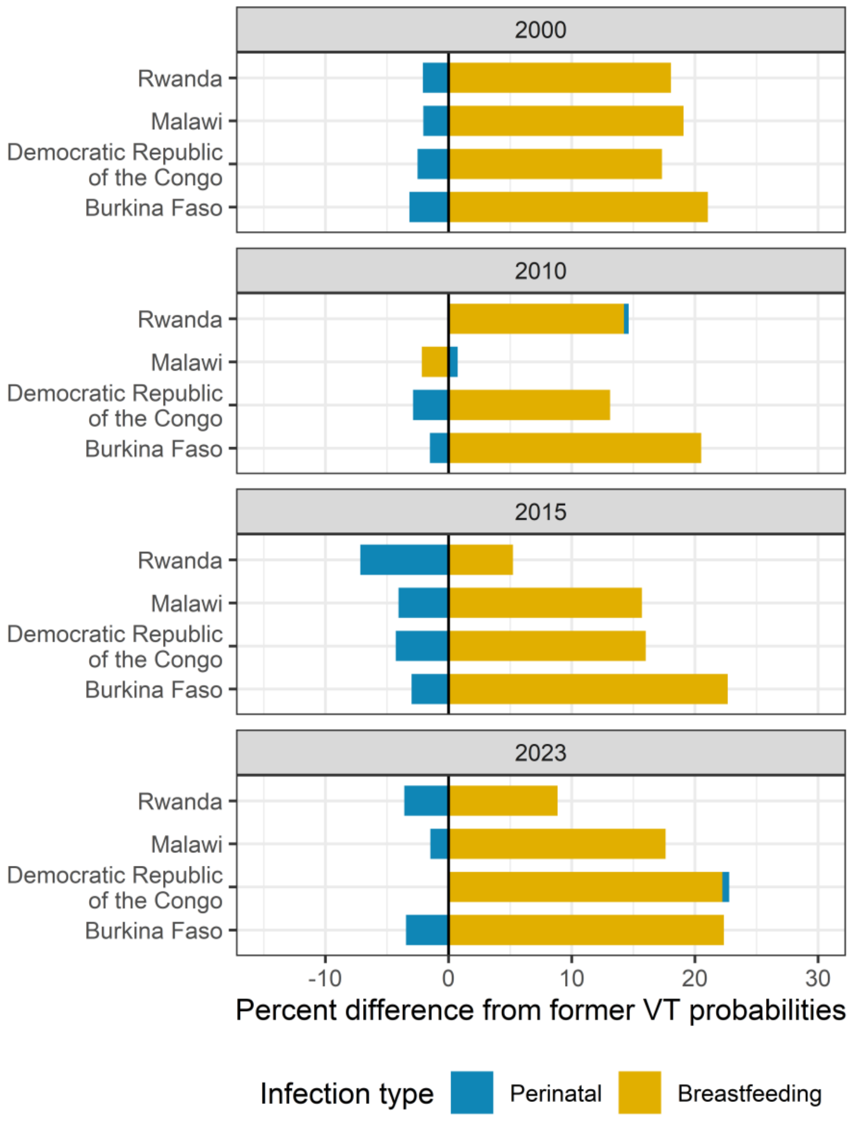

Figure 5.17. Summary of percent difference in the number of infections by infection type (perinatal or breastfeeding) between the former and updated vertical transmission probabilities for Rwanda, Malawi, Democratic Republic of the Congo, and Burkina Faso for the years 2000, 2010, 2015, and 2023.

6. Data included in meta-regression analysis

Table 6 summarises the studies that contributed data to the four meta-regression analyses on vertical transmission and to the analysis of viral load suppression at delivery. The table summarises key study characteristics (year, location, transmission timing[s] observed, preventive regimens studied, and sample size), annotates which model(s) for which each study contributed data, and whether the data were newly identified in the updated 2019-2024 search. All extracted data is also available in the supplementary file https://github.com/mwalte10/hiv_vt_mr/blob/main/data/public_data.csv.

Table 6. Study characteristics of studies included in models one through four and viral load suppression at delivery model

[1]Indicates whether the mother received PVT. If not, infection types are split into “Existing” or WLHIV who had HIV before the current pregnancy and “Infection” for mothers who seroconverted during pregnancy or breastfeeding.

[2]Yes indicates the paper was identified in the 2024 systematic review that searched for literature published between 2018-2024. No indicates the paper is from a previous review.

ID	Study	Study years	Location	Maternal PVT regimen or infection type[1]	Number of HIV exposed infants	Transmission timing	Model 1: No PVT	Model 2: Maternal seroconversion and short-course PVT	Model 3: Perinatal transmission from women on ART	Model 4: Breastfeeding transmission from women on ART	Viral suppression at delivery model	Added in 2024 systematic review[2]
1	**Aebi-Popp, 2022**	2019-2021	Switzerland	On ART	21	Perinatal			Included			Yes
					21	Breastfeeding				Included		
2	**Amone, 2023**	2016-2017	Uganda	Started ART	431	Perinatal			Included			No
3	**Bailey, 2011**	2000-2009	Europe	Started ART	1,760	Perinatal			Included			No
4	**Birkhead, 2010**	2002-2006	United States	Infection	41	Perinatal		Included				No
5	**Black, 2008**	2004-2007	South Africa	Started ART	302	Perinatal			Included			No
6	**Blonk, 2015**	2010-2014	Europe	On ART	7	Perinatal			Included		Included	No
7	**Bornhede, 2018**	2014-2017	Sweden	Started ART	3	Perinatal			Included		Included	No
				On ART	10				Included		Included	
8	**Carey, 2018**	2008-2014	United Kingdom	Started ART	67	Perinatal			Included			No
				On ART	65				Included			
9	**Chasela, 2010**	2006-2008	Malawi	Existing	668	Breastfeeding	Included					No
				Option A	748			Included				
10	**Chauhan, 2021**	2016-2018	India	On ART	32	Perinatal			Included			Yes
11	**Chen, 2019**	2007-2015	China	Started ART	446	Perinatal			Included			Yes
12	**Chibwesha, 2011**	2007-2010	Zambia	Option B	250	Perinatal		Included				No
				Started ART	1,813				Included			
13	**Choi, 2018**	2005-2017	Korea	Started ART	8	Perinatal			Included		Included	No
				On ART	8				Included		Included	
14	**Coetzee, 2019**	2010	South Africa	Existing	15	Perinatal	Included					Yes
				Dual ARV	8			Included				
				Started ART	44				Included			
				On ART	25				Included			

ID	Study	Study years	Location	Maternal PVT regimen or infection type[1]	Number of HIV exposed infants	Transmission timing	Model 1: No PVT	Model 2: Maternal seroconversion and short-course PVT	Model 3: Perinatal transmission from women on ART	Model 4: Breastfeeding transmission from women on ART	Viral suppression at delivery model	Added in 2024 systematic review[2]
15	**Cohan, 2015**	2009-2013	Uganda	Option B	353	Breastfeeding		Included				No
				Option B	374	Perinatal		Included				No
				Started ART	353	Breastfeeding				Included		No
				Started ART	389	Perinatal			Included			No
16	**Colbers, 2015**	Not reported	Europe	On ART	18	Perinatal			Included		Included	No
17	**Colbers, 2015**	Not reported	Europe	On ART	11	Perinatal			Included			No
18	**Colebunders, 1988**	1986	Democratic Republic of the Congo	Infection	3	Breastfeeding		Included				No
19	**Connor, 1994**	1991-1993	United States, France	Existing	183	Breastfeeding	Included					No
20	**Coovadia, 2013**	2008-2011	South Africa, Tanzania, Uganda, Zimbabwe	Existing	434	Breastfeeding	Included					No
				Dual ARV	819			Included				
				Option A	418			Included				
				Single dose Nevirapine, CD4 >350	434			Included				
				Single dose Nevirapine, CD4 <350	54			Included				
21	**Dabis, 1999**	1995-1998	Ivory Coast, Burkina Faso	Existing	113	Breastfeeding	Included					No
					145	Perinatal	Included					
22	**De Schacht, 2014**	2008-2011	Mozambique	Infection	29	Breastfeeding		Included				No
23	**Delicio, 2011**	2000-2009	Brazil	Started ART	12	Perinatal			Included			No
24	**Dinh, 2015**	2011-2012	South Africa	Infection	212	Perinatal		Included				No
25	**Dinh, 2018**	2013	Zimbabwe	Option B	338	Perinatal		Included				No
				On ART	415				Included			
26	**Dryden-Peterson, 2011**	2009-2010	Botswana	Option A	170	Perinatal		Included				No
				Started ART	114				Included			
				On ART	144				Included			
27	**Ejikunle, 2019**	2015-2016	Nigeria	Infection	5	Perinatal		Included				Yes

ID	Study	Study years	Location	Maternal PVT regimen or infection type[1]	Number of HIV exposed infants	Transmission timing	Model 1: No PVT	Model 2: Maternal seroconversion and short-course PVT	Model 3: Perinatal transmission from women on ART	Model 4: Breastfeeding transmission from women on ART	Viral suppression at delivery model	Added in 2024 systematic review[2]
**28**	**Ekpini, 1997**	1990-1994	Ivory Coast	Infection	12	Breastfeeding		Included				No
**29**	**Embree, 2000**	1986-1997	Kenya	Infection	12	Breastfeeding		Included				No
**30**	**Ewenighi-Amankwah, 2020**	Not reported	Nigeria	On ART	122	Perinatal			Included			Yes
**31**	**Finocchario-Kessler, 2015**	2010-2012	Kenya	Dual ARV	904	Perinatal		Included				No
				Option A	904			Included				
				Option B	219			Included				
**32**	**Flynn, 2018**	2011-2014	Malawi, South Africa, Zimbabwe, Uganda, Zambia, Tanzania, India	Dual ARV	503	Breastfeeding		Included				No
				Option A	503			Included				
				Option B	648			Included				
**33**	**Frange, 2020**	2010-2018	France	On ART	247	Perinatal			Included		Included	Yes
**34**	**Ganter, 2019**	2008-2014	France	Started ART	16	Perinatal			Included			Yes
				On ART	78				Included			
**35**	**Gibb, 2012**	2003-2009	Uganda, Zimbabwe	On ART	172	Perinatal			Included			No
					172	Breastfeeding				Included		
**36**	**Gill, 2017**	2013-2014	Rwanda	Option B	381	Breastfeeding		Included				No
				Started ART	205					Included		
				On ART	381					Included		
				Option B	205	Perinatal		Included				
				Started ART	205				Included			
				On ART	381				Included			
**37**	**Giuliano, 2014**	2008-2009	Malawi	Option B	276	Breastfeeding		Included				No
				Started ART	276					Included		
				Option B	278	Perinatal		Included				
				Started ART	278				Included			
	**Goga, 2015**	2010	South Africa	Dual ARV	1532	Perinatal		Included				No

ID	Study	Study years	Location	Maternal PVT regimen or infection type[1]	Number of HIV exposed infants	Transmission timing	Model 1: No PVT	Model 2: Maternal seroconversion and short-course PVT	Model 3: Perinatal transmission from women on ART	Model 4: Breastfeeding transmission from women on ART	Viral suppression at delivery model	Added in 2024 systematic review[2]
38				Option A	1532			Included				
39	**Goga, 2016**	2011-2013	South Africa	Dual ARV	2113	Perinatal		Included				No
				Existing	63		Included					
				Option A	2113			Included				
				Option B	890			Included				
				Started ART	890				Included			
40	**Goga, 2020**	2012-2014	South Africa	On ART	635	Perinatal			Included			Yes
41	**Guay, 1999**	1997-1999	Uganda	Single dose Nevirapine	310	Perinatal		Included				No
42	**Habib, 2021**	2015-2017	Iran	Existing	20	Perinatal	Included					Yes
43	**Harrington, 2019**	2015-2016	Malawi	Started ART	264	Perinatal			Included			Yes
44	**Hira, 1990**	1985-1986	Zambia	Infection	19	Breastfeeding		Included				No
45	**Hoffman, 2010**	2004-2008	South Africa	Existing	23	Perinatal	Included					No
				Single dose Nevirapine	1534			Included				
				Started ART	730				Included			
				On ART	143				Included			
46	**Humphrey, 2010**	1997-2000	Zimbabwe	Infection	334	Breastfeeding		Included				No
47	**Huntington, 2011**	1996-2009	United Kingdom	On ART	340	Perinatal			Included			No
48	**Iliff, 2005**	1997-2000	Zimbabwe	Existing	4367	Perinatal	Included					No
49	**João, 2012**	2013-2018	Argentina, Brazil, South Africa, Tanzania, Thailand, United States	Started ART	307	Perinatal			Included			Yes
50	**Kesho Bora, 2010**	2005-2008	Burkina Faso, Kenya, South Africa	Option B	154	Breastfeeding		Included				No
				Single dose Nevirapine, CD4 >350	283			Included				
				Single dose Nevirapine, CD4 <350	184			Included				

ID	Study	Study years	Location	Maternal PVT regimen or infection type[1]	Number of HIV exposed infants	Transmission timing	Model 1: No PVT	Model 2: Maternal seroconversion and short-course PVT	Model 3: Perinatal transmission from women on ART	Model 4: Breastfeeding transmission from women on ART	Viral suppression at delivery model	Added in 2024 systematic review[2]
**51**	**Kesho Bora, 2011**	2005-2008	Burkina Faso, Kenya, South Africa	Option A	284	Perinatal		Included				No
				Option B	333	Breastfeeding		Included				
					166	Perinatal		Included				
**52**	**Kilweo, 2009**	2004-2006	Tanzania	Option B	441	Breastfeeding		Included				No
				Started ART	423				Included			
				Option B	364	Perinatal		Included				
**53**	**Kim, 2013**	2009-2011	Malawi	On ART	262	Perinatal			Included			No
					262	Breastfeeding				Included		No
**54**	**Kuhn, 2010**	Not reported	Zambia	Existing	993	Breastfeeding	Included					No
**55**	**Lallemant, 2004**	2001-2003	Thailand	Dual ARV	636	Perinatal		Included				No
				Option A	508			Included				
**56**	**Le Roux, 2019**	Not reported	South Africa	Infection	7	Breastfeeding		Included				Yes
**57**	**Liang, 2009**	2007	China	Infection	106	Perinatal		Included				No
**58**	**Lima, 2016**	2008-2013	Brazil	Infection	9	Breastfeeding		Included				No
**59**	**Loh, 2021**	2008-2015	Singapore	Started ART	42	Perinatal			Included			Yes
				On ART	46				Included			
**60**	**Malaba, 2022**	2018	South Africa, Uganda	Started ART	268	Perinatal			Included			Yes
					268	Breastfeeding				Included		
**61**	**Mandelbrot, 2015**	2000-2011	France	Started ART	4267	Perinatal			Included		Included	No
				On ART	3505				Included		Included	
**62**	**Marazzi, 2010**	2005-2009	Malawi, Mozambique	Option B	2528	Breastfeeding		Included				No
				Started ART	2926					Included		
				Option B	3081	Perinatal		Included				
				Started ART	3081				Included			
**63**	**Marinda, 2011**	1997-2000	Zimbabwe	Existing	3285	Perinatal	Included					No
				Infection	422			Included				

ID	Study	Study years	Location	Maternal PVT regimen or infection type[1]	Number of HIV exposed infants	Transmission timing	Model 1: No PVT	Model 2: Maternal seroconversion and short-course PVT	Model 3: Perinatal transmission from women on ART	Model 4: Breastfeeding transmission from women on ART	Viral suppression at delivery model	Added in 2024 systematic review[2]
64	**Martinson, 2007**	2003-2005	South Africa	Single dose Nevirapine	108	Perinatal		Included				No
65	**Mayaux, 1995**	1986-1994	France	Existing	236	Perinatal	Included					No
66	**Meggi, 2018**	2014-2016	Mozambique	Option A	6	Perinatal		Included				Yes
67	**Meyers, 2015**	2010-2013	China	Started ART	1994	Perinatal			Included			No
68	**Moodley, 2003**	1999-2000	South Africa	Single dose Nevirapine	663	Perinatal		Included				No
69	**Myer, 2017**	2013-2014	South Africa	Option B	555	Perinatal		Included			Included	No
				Started ART	555				Included		Included	
70	**Namukwaya, 2011**	2007-2009	Uganda	Dual ARV	1161	Perinatal		Included				No
				Option A	1161			Included				
				Single dose Nevirapine	367			Included				
71	**Ndarukwa, 2019**	2014-2016	Zimbabwe	Started ART	841	Perinatal			Included			Yes
				On ART	289				Included			
72	**Nduati, 2000**	1992-1997	Kenya	Existing	165	Breastfeeding	Included					No
73	**Nesheim, 2007**	2001-2005	United States	Infection	4	Perinatal		Included				No
74	**Ngoma, 2015**	2008-2009	Zambia	Option B	219	Perinatal		Included				No
				Started ART	219				Included			
75	**Nlend, 2013**	2008-2012	Cameroon	Option A	110	Perinatal		Included				No
				Started ART	285				Included			
76	**Nyandiko, 2010**	2002-2007	Kenya	Single dose Nevirapine	69	Perinatal		Included				No
77	**Olana, 2016**	2006-2014	Ethiopia	Existing	102	Perinatal	Included					No
				Dual ARV	50			Included				
				Option A	50			Included				
78	**Ørbæk, 2017**	2002-2014	Denmark	On ART	247	Perinatal			Included			No
79	**Palasanthiran, 1993**	1980-1989	Australia	Infection	11	Breastfeeding		Included				No
80	**Pellowski, 2019**	2012-2015	South African	Option A	239	Perinatal		Included				Yes
	**Peltier, 2009**	2005-2007	Rwanda	Option B	532	Perinatal		Included				No

ID	Study	Study years	Location	Maternal PVT regimen or infection type[1]	Number of HIV exposed infants	Transmission timing	Model 1: No PVT	Model 2: Maternal seroconversion and short-course PVT	Model 3: Perinatal transmission from women on ART	Model 4: Breastfeeding transmission from women on ART	Viral suppression at delivery model	Added in 2024 systematic review[2]
81				Started ART	227				Included			
					227	Breastfeeding				Included		
82	Perry, 2016	2007-2012	United Kingdom	On ART	178	Perinatal			Included			No
				Started ART	493				Included			
83	Peters, 2017	2012-2014	United Kingdom	On ART	1749	Perinatal			Included			No
84	PETRA Study Team, 2002	1996-2000	Tanzania, South Africa, Uganda	Existing	303	Perinatal	Included					No
					303	Breastfeeding	Included					
85	Prieto, 2012	2000-2007	Spain	Existing	68	Perinatal	Included					No
				Started ART	244				Included			
86	Rollins, 2007	2004-2005	South Africa	Infection	172	Perinatal		Included				No
87	Roongpisuthipong, 2001	1992-1994	Thailand	Infection	15	Perinatal		Included				No
88	Sagna, 2015	2009-2013	Burkina Faso	Option A	136	Perinatal			Included			No
89	Salazar-Austin, 2018	2011-2014	South Africa	Dual ARV	48	Perinatal			Included			No
				Option A	48				Included			
				Option B	150				Included			
				Option B	171	Breastfeeding			Included			
90	Samuel, 2014	2004-2010	United Kingdom	On ART	68	Perinatal			Included		Included	No
91	Schalkwijk, 2017	Not reported	Europe	On ART	15	Perinatal			Included		Included	No
92	Scott, 2017	2002-2009	United States	Started ART	44	Perinatal			Included			No
93	Shaffer, 1999	1996-1997	Thailand	Existing	195	Perinatal	Included					No
94	Shapiro, 2006	2005-2006	Botswana	Dual ARV	345	Perinatal		Included				No
				Option A	345			Included				
95	Shapiro, 2010	2006-2008	Botswana	Option B	553	Perinatal		Included				No
				Option B	703	Breastfeeding		Included				
				Started ART	480					Included		
	Sibiude, 2023	2000-2017	France	Started ART	7448	Perinatal			Included		Included	No

ID	Study	Study years	Location	Maternal PVT regimen or infection type[1]	Number of HIV exposed infants	Transmission timing	Model 1: No PVT	Model 2: Maternal seroconversion and short-course PVT	Model 3: Perinatal transmission from women on ART	Model 4: Breastfeeding transmission from women on ART	Viral suppression at delivery model	Added in 2024 systematic review[2]
96				On ART	6606				Included		Included	
97	**SWEN Study Team, 2008**	2001-2007	Ethiopia, Uganda, India	Single dose Nevirapine	986	Perinatal		Included				No
98	**Thomas, 2011**	2003-2009	Kenya	Option B	487	Perinatal		Included				No
				Started ART	487				Included			
				Option B	522	Breastfeeding		Included				
				Started ART	457					Included		
99	**Tiam, 2019**	2014-2016	Lesotho	Started ART	370	Perinatal			Included			Yes
				On ART	249				Included			
100	**Tonwe-Gold, 2007**	2003-2005	Ivory Coast	Option A	122	Perinatal		Included				No
				Option B	52	Breastfeeding		Included				
				Single dose Nevirapine, CD4 >350	86			Included				
101	**Tookey, 2016**	2003-2013	United Kingdom	Started ART	2905	Perinatal			Included		Included	No
				On ART	968				Included		Included	
102	**Torpey, 2012**	2007-2010	Zambia	Option A	2366	Perinatal		Included				No
				Single dose Nevirapine	1143			Included				
103	**Tovo, 1991**	1980-1989	Italy	Infection	10	Perinatal		Included				No
104	**Townsend, 2014**	2000-2011	United Kingdon, Ireland	Existing	54	Perinatal	Included					No
				Started ART	3422				Included			
				On ART	2105				Included			
105	**Tubiana, 2013**	2007-2010	France	Started ART	36	Perinatal			Included		Included	No
106	**Van de Perre, 1991**	1988	Rwanda	Infection	15	Breastfeeding		Included				No
107	**Van Schalkwyk, 2013**	2008-2010	South Africa	Started ART	127	Perinatal			Included			No
108	**Wiktor, 1999**	1996-1998	Ivory Coast	Existing	119	Perinatal	Included					No
					115	Breastfeeding	Included					
	**Yusuf, 2022**	Not reported	United States	On ART	10	Perinatal			Included		Included	Yes

ID	Study	Study years	Location	Maternal PVT regimen or infection type[1]	Number of HIV exposed infants	Transmission timing	Model 1: No PVT	Model 2: Maternal seroconversion and short-course PVT	Model 3: Perinatal transmission from women on ART	Model 4: Breastfeeding transmission from women on ART	Viral suppression at delivery model	Added in 2024 systematic review[2]
109					9	Breastfeeding				Included		
110	Zijenah, 2022	2017-2018	Zimbabwe	Started ART	179	Perinatal			Included		Included	Yes
				On ART	272				Included		Included	
				Started ART	61	Breastfeeding				Included		

6.1 Study references

1. Aebi-Popp K, Kahlert CR, Crisinel PA, *et al.* Transfer of antiretroviral drugs into breastmilk: a prospective study from the Swiss Mother and Child HIV Cohort Study. *J Antimicrob Chemother* 2022; **77**: 3436–42.

2. Amone A, Gabagaya G, Wavamunno P, *et al.* Enhanced Peer-Group strategies to support prevention of Mother-to-Child HIV transmission leads to increased retention in care in Uganda: A Randomized controlled trial. *medRxiv* 2023. DOI:10.1101/2023.04.15.23288495.

3. Bailey H, European Collaborative Study in EuroCoord, Townsend C, Cortina-Borja M, Thorne C. Insufficient antiretroviral therapy in pregnancy: missed opportunities for prevention of mother-to-child transmission of HIV in Europe. *Antivir Ther* 2011; **16**: 895–903.

4. Birkhead GS, Pulver WP, Warren BL, Hackel S, Rodríguez D, Smith L. Acquiring human immunodeficiency virus during pregnancy and mother-to-child transmission in New York: 2002-2006. *Obstet Gynecol* 2010; **115**: 1247–55.

5. Black V, Hoffman RM, Sugar CA, *et al.* Safety and efficacy of initiating highly active antiretroviral therapy in an integrated antenatal and HIV clinic in Johannesburg, South Africa. *J Acquir Immune Defic Syndr* 2008; **49**: 276–81.

6. Blonk MI, Colbers APH, Hidalgo-Tenorio C, *et al.* Raltegravir in HIV-1–Infected pregnant women: pharmacokinetics, safety, and efficacy. *Clinical Infectious Diseases* 2015; **61**: 809–16.

7. Bornhede R, Soeria-Atmadja S, Westling K, Pettersson K, Navér L. Dolutegravir in pregnancy-effects on HIV-positive women and their infants. *Eur J Clin Microbiol Infect Dis* 2018; **37**: 495–500.

8. Carey L, Desouza C, Moorcroft A, Elgalib A. Pregnancy outcomes of women with HIV in a district general hospital in the UK. *J Obstet Gynaecol* 2018; **38**: 777–80.

9. Chasela CS, Hudgens MG, Jamieson DJ, *et al.* Maternal or infant antiretroviral drugs to reduce HIV-1 transmission. *N Engl J Med* 2010; **362**: 2271–81.

10. Chauhan N, Desai M, Shah S, Shah A, Gadhavi R. Treatment outcome of different antiretroviral drug regimens in HIV-positive pregnant women. *Perspectives in Clinical Research* 2021; **12**: 40–7.

11. Chen JC, Zhang Y, Rongkavilit C, *et al.* Growth of HIV-exposed infants in southwest China: a comparative study. *Glob Pediatr Health* 2019; **6**: 2333794X19854964.

12. Chibwesha CJ, Giganti MJ, Putta N, *et al.* Optimal time on HAART for prevention of mother-to-child transmission of HIV. *J Acquir Immune Defic Syndr* 2011; **58**: 224–8.

13. Choi H, Kim MH, Lee SJ, *et al.* Pregnancy rates and outcomes of HIV-infected women in Korea. *J Korean Med Sci* 2018; **33**: e296.

14. Coetzee M, Delport SD. Peripartum HIV infection in very low birth weight infants fed 'raw' mother's own milk. *South Afr J HIV Med* 2019; **20**: 912.

15. Cohan D, Natureeba P, Koss CA, *et al.* Efficacy and safety of lopinavir/ritonavir- versus efavirenz-based antiretroviral therapy in HIV-infected pregnant Ugandan women. *AIDS* 2015; **29**: 183–91.

16. Colbers A, Best B, Schalkwijk S, *et al.* Maraviroc pharmacokinetics in HIV-1–infected pregnant women. *Clin Infect Dis* 2015; **61**: 1582–9.

17. Colbers A, Moltó J, Ivanovic J, *et al.* Pharmacokinetics of total and unbound darunavir in HIV-1-infected pregnant women. *J Antimicrob Chemother* 2015; **70**: 534–42.

18. Colebunders R. Breastfeeding and transmission of HIV. *The Lancet* 1988; : 1487.

19. Connor EM, Sperling RS, Gelber R, *et al.* Reduction of maternal-infant transmission of human immunodeficiency virus type 1 with zidovudine treatment. *N Engl J Med* 1994; **331**: 1173–80.

20. Coovadia HM, Brown ER, Fowler MG, *et al.* Efficacy and safety of an extended nevirapine regimen in infant children of breastfeeding mothers with HIV-1 infection for prevention of postnatal HIV-1 transmission (HPTN 046): a randomised, double-blind, placebo-controlled trial. *Lancet* 2012; **379**: 221–8.

21. Dabis F, Msellati P, Meda N, *et al.* 6-month efficacy, tolerance, and acceptability of a short regimen of oral zidovudine to reduce vertical transmission of HIV in breastfed children in Côte d'Ivoire and Burkina Faso: a double-blind placebo-controlled multicentre trial. *The Lancet* 1999; **353**: 786–92.

22. De Schacht C, Mabunda N, Ferreira OC, *et al.* High HIV incidence in the postpartum period sustains vertical transmission in settings with generalized epidemics: a cohort study in Southern Mozambique. *J Int AIDS Soc* 2014; **17**: 18808.

23. Delicio AM, Milanez H, Amaral E, *et al.* Mother-to-child transmission of human immunodeficiency virus in a ten years period. *Reproductive Health* 2011; **8**: 35.

24. Dinh T-H, Delaney KP, Goga A, *et al.* Impact of maternal HIV seroconversion during pregnancy on early mother to child transmission of hiv (MTCT) measured at 4-8 weeks postpartum in South Africa 2011-2012: a national population-based evaluation. *PLOS ONE* 2015; **10**: e0125525.

25. Dinh T-H, Mushavi A, Shiraishi RW, *et al.* Impact of timing of antiretroviral treatment and birth weight on mother-to-child human immunodeficiency virus transmission: findings from an 18-month prospective cohort of a nationally representative sample of mother-infant pairs during the transition from Option A to Option B+ in Zimbabwe. *Clin Infect Dis* 2018; **66**: 576–85.

26. Dryden-Peterson S, Jayeoba O, Hughes MD, *et al.* Highly active antiretroviral therapy versus zidovudine for prevention of mother-to-child transmission in a programmatic setting, Botswana. *J Acquir Immune Defic Syndr* 2011; **58**: 353–7.

27. Ejikunle SD, Mbachu II, Okeudo C, Dike E, Ejikem E. Incident HIV infection and perinatal transmission rates among HIV negative pregnant women who retested in labor in a tertiary health centre, South East Nigeria. *Niger J Clin Pract* 2019; **22**: 1341–8.

28. Ekpini ER, Wiktor SZ, Satten GA, *et al.* Late postnatal mother-to-child transmission of HIV-1 in Abidjan, Côte d'Ivoire. *Lancet* 1997; **349**: 1054–9.

29. Embree JE, Njenga S, Datta P, *et al.* Risk factors for postnatal mother-child transmission of HIV-1. *AIDS* 2000; **14**: 2535–41.

30. Ewenighi-Amankwah CO, Onyenekwe CC, Udemba O, Muogbo P, Rong L. A mother-to-child transmission study in Nigeria: the impact of maternal HIV infection and HAART on plasma immunoglobulins, cytokine profiles and infant outcome. *Virlogica Sinica* 2020; **35**: 468–77.

31. Finocchario-Kessler S, Clark KF, Khamadi S, *et al.* Progress toward eliminating mother to child transmission of HIV in Kenya: review of treatment guideline uptake and pediatric transmission at four government hospitals between 2010 and 2012. *AIDS and Behavior* 2015; **20**: 2302–611.

32. Flynn PM, Taha TE, Cababasay M, *et al.* Prevention of HIV-1 transmission through breastfeeding: efficacy and safety of maternal antiretroviral therapy versus infant nevirapine prophylaxis for duration of breastfeeding in HIV-1-infected women with high CD4 cell count (IMPAACT PROMISE): a randomized, open-label, clinical trial. *J Acquir Immune Defic Syndr* 2018; **77**: 383–92.

33. Frange P, Tubiana R, Sibiude J, *et al.* Rilpivirine in HIV-1-positive women initiating pregnancy: to switch or not to switch? *J Antimicrob Chemother* 2020; **75**: 1324–31.

34. Gantner P, Sylla B, Morand-Joubert L, *et al.* "Real life" use of raltegravir during pregnancy in France: The Coferal-IMEA048 cohort study. *PLoS ONE* 2019; **14**. DOI:10.1371/journal.pone.0216010.

35. Gibb DM, Kizito H, Russell EC, *et al.* Pregnancy and infant outcomes among HIV-infected women taking long-term ART with and without tenofovir in the DART trial. *PLoS Med* 2012; **9**: e1001217.

36. Gill MM, Hoffman HJ, Ndatimana D, *et al.* 24-month HIV-free survival among infants born to HIV-positive women enrolled in Option B+ program in Kigali, Rwanda. *Medicine (Baltimore)* 2017; **96**: e9445.

37. Giuliano M, Andreotti M, Liotta G, *et al.* Maternal antiretroviral therapy for the prevention of mother-to-child transmission of HIV in Malawi: maternal and infant outcomes two years after delivery. *PLOS ONE* 2013; **8**: e68950.

38. Goga AE, Lombard C, Jackson D, *et al.* Impact of breastfeeding, maternal antiretroviral treatment and health service factors on 18-month vertical transmission of HIV and HIV-free survival: results from a nationally representative HIV-exposed infant cohort, South Africa. *J Epidemiol Community Health* 2020; **74**: 1069–77.

39. Goga AE, Dinh T-H, Jackson DJ, *et al.* First population-level effectiveness evaluation of a national programme to prevent HIV transmission from mother to child, South Africa. *J Epidemiol Community Health* 2015; **69**: 240–8.

40. Goga AE, Dinh T, Jackson DJ, *et al.* Population–level effectiveness of PMTCT Option A on early mother–to–child (MTCT) transmission of HIV in South Africa: implications for eliminating MTCT. *J Glob Health* 2016; **6**: 020405.

41. Guay LA, Musoke P, Fleming T, *et al.* Intrapartum and neonatal single-dose nevirapine compared with zidovudine for prevention of mother-to-child transmission of HIV-1 in Kampala, Uganda: HIVNET 012 randomised trial. *Lancet* 1999; **354**: 795–802.

42. Habib Z, Bokharaei-Salim F, Kiani SJ, *et al.* Non detection of HIV-1 proviral DNA in PBMCS of the neonates born to Iranian HIV-infected mothers in PMTCT program. *Archives of Pediatric Infectious Diseases* 2021; **9**. DOI:10.5812/pedinfect.105098.

43. Harrington BJ, DiPrete BL, Jumbe AN, *et al.* Safety and efficacy of Option B+ ART in Malawi: few severe maternal toxicity events or infant HIV infections among pregnant women initiating tenofovir/lamivudine/efavirenz. *Trop Med Int Health* 2019; **24**: 1221–8.

44. Hira, Ug M, C M, *et al.* Apparent vertical transmission of human immunodeficiency virus type 1 by breast-feeding in Zambia. *The Journal of pediatrics* 1990; **117**. DOI:10.1016/s0022-3476(05)81084-4.

45. Hoffman R, Black V, Technau K, *et al.* Effects of highly active antiretroviral therapy duration and regimen on risk for mother-to-child transmission of HIV in Johannesburg, South Africa. *J Acquir Immune Defic Syndr* 2010; **54**: 35–41.

46. Humphrey JH, Marinda E, Mutasa K, *et al.* Mother to child transmission of HIV among Zimbabwean women who seroconverted postnatally: prospective cohort study. *BMJ* 2010; **341**: c6580.

47. Huntington SE, Bansi LK, Thorne C, *et al.* Treatment switches during pregnancy among HIV-positive women on antiretroviral therapy at conception. *AIDS* 2011; **25**: 1647–55.

48. Iliff PJ, Piwoz EG, Tavengwa NV, *et al.* Early exclusive breastfeeding reduces the risk of postnatal HIV-1 transmission and increases HIV-free survival. *AIDS* 2005; **19**: 699–708.

49. João EC, Morrison RL, Shapiro DE, *et al.* Raltegravir versus efavirenz in antiretroviral-naive pregnant women living with HIV (NICHD P1081): an open-label, randomised, controlled, phase 4 trial. *Lancet HIV* 2020; **7**: e322–31.

50. Kesho Bora Study Group. Eighteen-month follow-up of HIV-1-infected mothers and their children enrolled in the Kesho Bora study observational cohorts. *J Acquir Immune Defic Syndr* 2010; **54**: 533–41.

51. Kesho Bora Study Group, de Vincenzi I. Triple antiretroviral compared with zidovudine and single-dose nevirapine prophylaxis during pregnancy and breastfeeding for prevention of mother-to-child transmission of HIV-1 (Kesho Bora study): a randomised controlled trial. *Lancet Infect Dis* 2011; **11**: 171–80.

52. Kilewo C, Karlsson K, Ngarina M, *et al.* Prevention of mother-to-child transmission of HIV-1 through breastfeeding by treating mothers with triple antiretroviral therapy in Dar es Salaam, Tanzania: the Mitra Plus study. *J Acquir Immune Defic Syndr* 2009; **52**: 406–16.

53. Kim MH, Ahmed S, Preidis GA, *et al.* Low rates of mother-to-child HIV Transmission in aroutine programmatic setting in Lilongwe, Malawi. *PLOS ONE* 2013; **8**: e64979.

54. Kuhn L, Aldrovandi GM, Sinkala M, Kankasa C, Mwiya M, Thea DM. Potential impact of new WHO criteria for antiretroviral treatment for prevention of mother-to- child HIV transmission. *AIDS* 2010; **24**: 1374–7.

55. Lallemant M, Perinatal HIV Prevention Trial (Thailand) Investigators. Single-dose perinatal nevirapine plus standard zidovudine to prevent mother-to-child transmission of HIV-1 in Thailand. *New England Journal of Medicine* 2004; **351**. DOI:10.1056/NEJMoa033500.

56. le Roux SM, Abrams EJ, Nguyen KK, Myer L. HIV incidence during breastfeeding and mother-to-child transmission in Cape Town, South Africa. *AIDS* 2019; **33**: 1399–401.

57. Liang K, Gui X, Zhang Y-Z, Zhuang K, Meyers K, Ho DD. A case series of 104 women infected with HIV-1 via blood transfusion postnatally: high rate of HIV-1 transmission to infants through breast-feeding. *J Infect Dis* 2009; **200**: 682–6.

58. Lima YAR, Cardoso LPV, Reis MN da G, Stefani MMA. Incident and long-term HIV-1 infection among pregnant women in Brazil: Transmitted drug resistance and mother-to-child transmission. *J Med Virol* 2016; **88**: 1936–43.

59. Loh M, Thoon KC, Mathur M, Kathirvel R. Management of HIV-positive pregnant women: a Singapore experience. *Singapore Med J* 2021; **62**: 599–603.

60. Malaba TR, Nakatudde I, Kintu K, *et al.* 72 weeks post-partum follow-up of dolutegravir versus efavirenz initiated in late pregnancy (DolPHIN-2): an open-label, randomised controlled study. *Lancet HIV* 2022; **9**: e534–43.

61. Mandelbrot L, Tubiana R, Le Chenadec J, *et al.* No perinatal HIV-1 transmission from women with effective antiretroviral therapy starting before conception. *Clinical Infectious Diseases* 2015; **61**: 1715–25.

62. Marazzi MC, Liotta G, Nielsen-Saines K, *et al.* Extended antenatal antiretroviral use correlates with improved infant outcomes throughout the first year of life. *AIDS* 2010; **24**: 2819.

63. Marinda ET, Moulton LH, Humphrey JH, *et al.* In utero and intra-partum HIV-1 transmission and acute HIV-1 infection during pregnancy: using the BED capture enzyme-immunoassay as a surrogate marker for acute infection. *Int J Epidemiol* 2011; **40**: 945–54.

64. Martinson NA, Ekouevi DK, Dabis F, *et al.* Transmission rates in consecutive pregnancies exposed to single-dose nevirapine in Soweto, South Africa and Abidjan, Côte d'Ivoire. *J Acquir Immune Defic Syndr* 2007; **45**: 206–9.

65. Mayaux MJ, Blanche S, Rouzioux C, *et al.* Maternal factors associated with perinatal HIV-1 transmission: the French Cohort Study: 7 years of follow-up observation. The French Pediatric HIV Infection Study Group. *J Acquir Immune Defic Syndr Hum Retrovirol* 1995; **8**: 188–94.

66. Meggi B, Vojnov L, Mabunda N, *et al.* Performance of point-of-care birth HIV testing in primary health care clinics: An observational cohort study. *PLoS One* 2018; **13**: e0198344.

67. Meyers K, Qian H, Wu Y, *et al.* Early initiation of ARV during pregnancy to move towards virtual elimination of mother-to-child-transmission of HIV-1 in Yunnan, China. *PLOS ONE* 2015; **10**: e0138104.

68. Moodley D, Moodley J, Coovadia H, *et al.* A multicenter randomized controlled trial of nevirapine versus a combination of zidovudine and lamivudine to reduce intrapartum and early postpartum mother-to-child transmission of human immunodeficiency virus type 1. *J Infect Dis* 2003; **187**: 725–35.

69. Myer L, Phillips TK, McIntyre JA, *et al.* HIV viraemia and mother-to-child transmission risk after antiretroviral therapy initiation in pregnancy in Cape Town, South Africa. *HIV Med* 2017; **18**: 80–8.

70. Namukwaya Z, Mudiope P, Kekitiinwa A, *et al.* The impact of maternal highly active antiretroviral therapy and short-course combination antiretrovirals for prevention of mother-to-child transmission on early infant infection rates at the Mulago national referral hospital in Kampala, Uganda, January 2007 to May 2009. *Journal of Acquired Immune Deficiency Syndromes* 2011; **56**: 69.

71. Ndarukwa V, Zunza M. Combination antiretroviral treatment use in prevention of mother-to-child transmission programmes: 6-week HIV prevalence and relationship to time of antiretroviral treatment initiation and mixed feeding. *S Afr J Infect Dis* 2019; **34**: 117.

72. Nduati R, John G, Mbori-Ngacha D, *et al.* Effect of breastfeeding and formula feeding on transmission of HIV-1: a randomized clinical trial. *JAMA* 2000; **283**: 1167–74.

73. Nesheim S, Jamieson DJ, Danner SP, *et al.* Primary human immunodeficiency virus infection during pregnancy detected by repeat testing. *Am J Obstet Gynecol* 2007; **197**: 149.e1-5.

74. Ngoma MS, Misir A, Mutale W, *et al.* Efficacy of WHO recommendation for continued breastfeeding and maternal cART for prevention of perinatal and postnatal HIV transmission in Zambia. *J Int AIDS Soc* 2015; **18**: 19352.

75. Njom Nlend AE, Same Ekobo C, Bagfegue Ekani B, *et al.* Preventing HIV-1 transmission in breastfed infants in low resource settings: early HIV infection and late postnatal transmission in a routine prevention of mother-to-child transmission program in Yaounde, Cameroon. *J Trop Pediatr* 2013; **59**: 387–92.

76. Nyandiko WM, Otieno-Nyunya B, Musick B, *et al.* Outcomes of HIV-exposed children in western Kenya: efficacy of prevention of mother to child transmission in a resource-constrained setting. *J Acquir Immune Defic Syndr* 2010; **54**: 42–50.

77. Olana T, Bacha T, Worku W, Tadesse BT. Early infant diagnosis of HIV infection using DNA-PCR at a referral center: an 8 years retrospective analysis. *AIDS Res Ther* 2016; **13**: 29.

78. Ørbaek M, Thorsteinsson K, Helleberg M, *et al.* Assessment of mode of delivery and predictors of emergency caesarean section among women living with HIV in a matched-pair setting with women from the general population in Denmark, 2002−2014. *HIV Medicine* 2017; **18**: 736–47.

79. Palasanthiran P, Ziegler JB, Stewart GJ, *et al.* Breast-feeding during primary maternal human immunodeficiency virus infection and risk of transmission from mother to infant. *J Infect Dis* 1993; **167**: 441–4.

80. Pellowski J, Wedderburn C, Stadler JAM, *et al.* Implementation of prevention of mother-to-child transmission (PMTCT) in South Africa: outcomes from a population-based birth cohort study in Paarl, Western Cape. *BMJ Open* 2019; **9**: e033259.

81. Peltier C-A, Ndayisaba G-F, Lepage P, *et al.* Breastfeeding with maternal antiretroviral therapy or formula feeding to prevent HIV postnatal mother-to-child transmission in Rwanda. *AIDS* 2009; **23**: 2415–23.

82. Perry MEO, Taylor GP, Sabin CA, *et al.* Lopinavir and atazanavir in pregnancy: comparable infant outcomes, virological efficacies and preterm delivery rates. *HIV Med* 2016; **17**: 28–35.

83. Peters H, Francis K, Sconza R, *et al.* UK mother-to-child HIV transmission rates continue to decline: 2012-2014. *Clin Infect Dis* 2017; **64**: 527–8.

84. Petra Study Team. Efficacy of three short-course regimens of zidovudine and lamivudine in preventing early and late transmission of HIV-1 from mother to child in Tanzania, South

Africa, and Uganda (Petra study): a randomised, double-blind, placebo-controlled trial. *Lancet* 2002; **359**: 1178–86.

85. Prieto LM, Madrid Cohort of HIV-Infected Mother-Infant Pairs. Low rates of mother-to-child transmission of HIV-1 and risk factors for infection in Spain: 2000-2007. *Pediatric Infectious Disease Journal* 2012; **31**: 1053–8.

86. Rollins N, Little K, Mzolo S, Horwood C, Newell M-L. Surveillance of mother-to-child transmission prevention programmes at immunization clinics: the case for universal screening. *AIDS* 2007; **21**: 1341–7.

87. Roongpisuthipong A, Siriwasin W, Simonds RJ, *et al.* HIV seroconversion during pregnancy and risk for mother-to-infant transmission. *J Acquir Immune Defic Syndr* 2001; **26**: 348–51.

88. Sagna T, Bisseye C, Compaore TR, *et al.* Prevention of mother-to-child HIV-1 transmission in Burkina Faso: evaluation of vertical transmission by PCR, molecular characterization of subtypes and determination of antiretroviral drugs resistance. *Glob Health Action* 2015; **8**: 26065.

89. Salazar-Austin N, Hoffmann J, Cohn S, *et al.* Poor obstetric and infant outcomes in human immunodeficiency virus-infected pregnant women with tuberculosis in South Africa: The Tshepiso Study. *Clin Infect Dis* 2018; **66**: 921–9.

90. Samuel M, Bradshaw D, Perry M, *et al.* Antenatal atazanavir: a retrospective analysis of pregnancies exposed to atazanavir. *Infect Dis Obstet Gynecol* 2014; **2014**: 961375.

91. Schalkwijk S, Colbers A, Konopnicki D, *et al.* Lowered rilpivirine exposure during the third trimester of pregnancy in human immunodeficiency virus type 1-Infected women. *Clin Infect Dis* 2017; **65**: 1335–41.

92. Scott GB, Brogly SB, Muenz D, Stek AM, Read JS. Missed opportunities for prevention of mother-to-child transmission of human immunodeficiency virus. *Obstet Gynecol* 2017; **129**: 621–8.

93. Shaffer N, Chuachoowong R, Mock PA, *et al.* Short-course zidovudine for perinatal HIV-1 transmission in Bangkok, Thailand: a randomised controlled trial. *The Lancet* 1999; **353**: 773–80.

94. Shapiro RL, Hughes MD, Ogwu A, *et al.* Antiretroviral regimens in pregnancy and breast-feeding in Botswana. *N Engl J Med* 2010; **362**: 2282–94.

95. Shapiro RL, Thior I, Gilbert PB, *et al.* Maternal single-dose nevirapine versus placebo as part of an antiretroviral strategy to prevent mother-to-child HIV transmission in Botswana. *AIDS* 2006; **20**: 1281.

96. Sibiude J, Chenadec J le, Mandelbrot L, *et al.* Update of perinatal Human immunodeficiency virus type 1 transmission in France: zero transmission for 5482 mothers

on continuous antiretroviral therapy from conception and with undetectable viral load at delivery. *Clinical Infectious Diseases* 2022; **76**: e590–8.

97. Six Week Extended-Dose Nevirapine (SWEN) Study Team, Bedri A, Gudetta B, *et al.* Extended-dose nevirapine to 6 weeks of age for infants to prevent HIV transmission via breastfeeding in Ethiopia, India, and Uganda: an analysis of three randomised controlled trials. *Lancet* 2008; **372**: 300–13.

98. Thomas TK, Masaba R, Borkowf CB, *et al.* Triple-antiretroviral prophylaxis to prevent mother-to-child HIV transmission through breastfeeding—The Kisumu Breastfeeding Study, Kenya: A Clinical Trial. *PLoS Med* 2011; **8**: e1001015.

99. Tiam A, Kassaye SG, Machekano R, *et al.* Comparison of 6-week PMTCT outcomes for HIV-exposed and HIV-unexposed infants in the era of lifelong ART: Results from an observational prospective cohort study. *PLoS One* 2019; **14**: e0226339.

100. Tonwe-Gold B, Ekouevi DK, Viho I, *et al.* Antiretroviral treatment and prevention of peripartum and postnatal HIV transmission in West Africa: evaluation of a two-tiered approach. *PLoS Med* 2007; **4**: e257.

101. Tookey PA, Thorne C, van Wyk J, Norton M. Maternal and foetal outcomes among 4118 women with HIV infection treated with lopinavir/ritonavir during pregnancy: analysis of population-based surveillance data from the national study of HIV in pregnancy and childhood in the United Kingdom and Ireland. *BMC Infectious Diseases* 2016; **16**: 65.

102. Torpey K, Mandala J, Kasonde P, *et al.* Analysis of HIV early infant diagnosis data to estimate rates of perinatal HIV transmission in Zambia. *PLOS ONE* 2012; **7**: e42859.

103. Tovo PA, Palomba E, Gabiano C, Galli L, de Martino M. Human immunodeficiency virus type 1 (HIV-1) seroconversion during pregnancy does not increase the risk of perinatal transmission. *Br J Obstet Gynaecol* 1991; **98**: 940–2.

104. Townsend CL, Byrne L, Cortina-Borja M, *et al.* Earlier initiation of ART and further decline in mother-to-child HIV transmission rates, 2000-2011. *AIDS* 2014; **28**: 1049–57.

105. Tubiana R, Mandelbrot L, Le Chenadec J, *et al.* Lopinavir/ritonavir monotherapy as a nucleoside analogue-sparing strategy to prevent HIV-1 mother-to-child transmission: the ANRS 135 PRIMEVA phase 2/3 randomized trial. *Clin Infect Dis* 2013; **57**: 891–902.

106. Van de Perre P, Simonon A, Msellati P, *et al.* Postnatal transmission of human immunodeficiency virus type 1 from mother to infant. A prospective cohort study in Kigali, Rwanda. *N Engl J Med* 1991; **325**: 593–8.

107. Van Schalkwyk M, Andersson MI, Zeier MD, La Grange M, Taljaard JJ, Theron GB. The impact of revised PMTCT guidelines: a view from a public sector ARV clinic in Cape Town, South Africa. *J Acquir Immune Defic Syndr* 2013; **63**: 234–8.

108. Wiktor SZ, Ekpini E, Karon JM, *et al.* Short-course oral zidovudine for prevention of mother-to-child transmission of HIV-1 in Abidjan, Côte d'Ivoire: a randomised trial. *Lancet* 1999; **353**: 781–5.

109. Yusuf HE, Knott-Grasso MA, Anderson J, *et al.* Experience and outcomes of breastfed infants of women living with HIV in the United States: findings from a single-center breastfeeding support initiative. *J Pediatric Infect Dis Soc* 2022; **11**: 24–7.

110. Zijenah LS, Bandason T, Bara W, Chipiti MM, Katzenstein DA. Impact of Option B+ combination antiretroviral therapy on mother-to-child transmission of HIV-1, maternal and infant virologic responses to combination antiretroviral therapy, and maternal and infant mortality rates: a 24-month prospective follow-up study. *AIDS Patient Care and STDs* 2022; **36**: 145–52.

The following references were included in previous systematic reviews but were excluded from this analysis.

111. Becquet R. Duration, Pattern of Breastfeeding and Postnatal Transmission of HIV: Pooled Analysis of Individual Data from West and South African Cohorts | PLOS ONE. https://journals.plos.org/plosone/article?id=10.1371/journal.pone.0007397 (accessed July 26, 2024).

112. Department of HIV/AIDS Prevention and Care. Report from the national program for Early Infant Diagnosis of HIV. Gaborone, Botswana: Botswana Ministry of Health, 2011.

113. Dunn DT, Newell ML, Ades AE, Peckham CS. Risk of human immunodeficiency virus type 1 transmission through breastfeeding. *Lancet* 1992; 340: 585–8.

114. Ewing A. Personal communication on HIV transmission among women receiving sdNVP in Malawi. 2018.

115. Fowler MG. PROMISE: Efficacy and safety of two strategies to prevent perinatal HIV transmission. 2015.

116. Leroy V, Sakarovitch C, Cortina-Borja M, *et al.* Is there a difference in the efficacy of peripartum antiretroviral regimens in reducing mother-to-child transmission of HIV in Africa? *AIDS* 2005; 19: 1865.

117. Mofenson LM. Randomized controlled study (PEPI-Malawi) comparing control standard sdNVP infant prophylaxis to extend regimens. 2009; published online July.

118. Mugerwa. Early infection among Ugandan HIV-exposed infants whose mothers received Option B+ or Option A. 2014.

119. Nagot N, Kankasa C, Tumwine JK, *et al.* Extended pre-exposure prophylaxis with lopinavir–ritonavir versus lamivudine to prevent HIV-1 transmission through breastfeeding up to 50 weeks in infants in Africa (ANRS 12174): a randomised controlled trial. *The Lancet* 2016; 387: 566–73.

120. Ocheke AN, Samuels E, Ocheke IE, *et al.* An Audit of Perineal Trauma and Vertical Transmission Of HIV. *African Journal of Reproductive Health* 2017; **21**: 67–72.

121. Sagay AS, Ebonyi AO, Meloni ST, *et al.* Mother-to-child transmission outcomes of HIV-exposed infants followed up in Jos North-central Nigeria. *Curr HIV Res* 2015; 13: 193–200.

122. Singh A. HIV seroconversion during pregnancy and mother-to-child HIV transmission: data from the Enhanced Perinatal Surveillance Project, United States, 2005–2010 [abstract]. 2012.

123. Tiam A, Kassaye S, Machekano R, *et al.* Shifting dynamics of HIV transmission timing among infants in the era of option B+ and implications for infant testing. https://ias2017.org/Portals/1/Files/IAS2017_LO.compressed4c6a.pdf?fileticket=m3LSDs1z4QY%3d&tabid=577&portalid=1#page=260.28.

7. References

1 Stover J, Glaubius R. Methods and assumptions for estimating key HIV indicators in the UNAIDS annual estimates processs. *J Acquir Immune Defic Syndr* 2024; **95**: e5.

2 Stover J, Glaubius R, Mofenson L, *et al.* Updates to the Spectrum/AIM model for estimating key HIV indicators at national and subnational levels. *AIDS Lond Engl* 2019; **33**: S227–34.

3 Rollins N, Mahy M, Becquet R, Kuhn L, Creek T, Mofenson L. Estimates of peripartum and postnatal mother-to-child transmission probabilities of HIV for use in Spectrum and other population-based models. *Sex Transm Infect* 2012; **88**: i44–51.

4 Govender S, Otwombe K, Essien T, *et al.* CD4 counts and viral loads of newly diagnosed HIV-infected individuals: implications for treatment as prevention. *PLOS ONE* 2014; **9**: e90754.

5 Leroy V, Montcho C, Manigart O, *et al.* Maternal plasma viral load, zidovudine and mother-to-child transmission of HIV-1 in Africa: DITRAME ANRS 049a trial. *AIDS Lond Engl* 2001; **15**: 517–22.

6 Mussini C, Pinti M, Bugarini R, *et al.* Effect of treatment interruption monitored by CD4 cell count on mitochondrial DNA content in HIV-infected patients: a prospective study. *AIDS* 2005; **19**: 1627.

7 Margulis AV, Calingaert B, Kawai AT, Rivero-Ferrer E, Anthony MS. Distribution of gestational age at birth by maternal and infant characteristics in U.S. birth certificate data: Informing gestational age assumptions when clinical estimates are not available. *Pharmacoepidemiol Drug Saf* 2023; **32**: 1012–20.

8 Siqithi S, Durojaiye OC, Adeniyi OV. Effects of the timing of maternal antiretroviral therapy initiation, CD4 count, and HIV viral load on birth outcomes in the Eastern Cape province of South Africa: A secondary data analysis. *PLOS ONE* 2024; **19**: e0308374.
